# Extended similarity indices: the benefits of comparing more than two objects simultaneously. Part 1: Theory and characteristics^†^

**DOI:** 10.1186/s13321-021-00505-3

**Published:** 2021-04-23

**Authors:** Ramón Alain Miranda-Quintana, Dávid Bajusz, Anita Rácz, Károly Héberger

**Affiliations:** 1 Department of Chemistry, University of Florida, Gainesville, FL 32603 USA; 2 Medicinal Chemistry Research Group, Research Centre for Natural Sciences, Magyar tudósok krt. 2, 1117 Budapest, Hungary; 3 Plasma Chemistry Research Group, ELKH Research Centre for Natural Sciences, Magyar tudósok krt. 2, 1117 Budapest, Hungary

**Keywords:** Comparisons, Rankings, Extended similarity indices, Consistency, Molecular fingerprints, ANOVA, Sum of ranking differences

## Abstract

Quantification of the similarity of objects is a key concept in many areas of computational science. This includes cheminformatics, where molecular similarity is usually quantified based on binary fingerprints. While there is a wide selection of available molecular representations and similarity metrics, there were no previous efforts to extend the computational framework of similarity calculations to the simultaneous comparison of more than two objects (molecules) at the same time. The present study bridges this gap, by introducing a straightforward computational framework for comparing multiple objects at the same time and providing extended formulas for as many similarity metrics as possible. In the binary case (*i.e.* when comparing two molecules pairwise) these are naturally reduced to their well-known formulas. We provide a detailed analysis on the effects of various parameters on the similarity values calculated by the extended formulas. The extended similarity indices are entirely general and do not depend on the fingerprints used. Two types of variance analysis (ANOVA) help to understand the main features of the indices: (i) ANOVA of mean similarity indices; (ii) ANOVA of sum of ranking differences (SRD). Practical aspects and applications of the extended similarity indices are detailed in the accompanying paper: Miranda-Quintana *et al.* J Cheminform. 2021. 10.1186/s13321-021-00504-4. Python code for calculating the extended similarity metrics is freely available at: https://github.com/ramirandaq/MultipleComparisons.

**Supplementary Information:**

The online version contains supplementary material available at 10.1186/s13321-021-00505-3.

## Introduction

A large number of molecular representations exist, and there are several methods (similarity and distance measures) to quantify the similarity of molecular representations [1, 2]. These similarity and distance measures (coefficients) accompany the entire process of drug design: virtual screening, [3] hit-to-lead optimization, [4] QSAR modeling, [5] finding activity cliffs, [6] drug target prediction, [7] etc.

Molecular similarity has been established as the basis of ligand-based virtual screening, as well as molecular informatics (a collective term encompassing various specific applications of cheminformatics principles, such as compound library design or molecular property predictions) [8]. Information theory has also provided some metrics on similarity. However, molecular similarity plays a crucial role in quantum chemistry as well [9–14]. The merits of pairwise fingerprint comparisons have been exhausted on a large scale [15]. Todeschini *et al.* summarized many of the binary similarity coefficients that have been developed so far [1, 16].

In our earlier works we have investigated the applicability of binary similarity coefficients, proved their equivalency or superiority [17–19]. We could find better similarity coefficents than the most frequently applied Tanimoto index [2] and formulated constraints about finding the best equations for fitting data [20].

It is somewhat odd that the similarity evaluations are exclusively based on pairwise comparisons of two molecules. By analogy, multiple linear regression is not exclusively based on univariate correlations between each predictor and the response, but takes into account multiple correlations. (Two descriptors together might be significant in a model predicting the response whereas none of them correlates with it significantly alone.) Hence, it is natural to consider the extension of the standard comparative indices such that they can be used to compare more than two objects (*e.g*., molecules, fingerprints) at a time. This will provide unparalleled flexibility to the traditional algorithms that aim to quantify molecular similarity, since one will have the freedom of choosing how many molecules are to be compared simultaneously. This will in turn allow us to gain further insight regarding the relations among the compounds in a given dataset (by providing more complete measures of chemical diversity), which can then be used to shed more light on their structures and properties.

While to our knowledge, comparing multiple objects at the same time has not yet been introduced for molecular fingerprints, it is worth to note that other studies have combined multiple comparative measures in other contexts in the field. For example, in a recent study, an iterative virtual screening strategy was designed and evaluated on 25 diverse bioactivity data sets from ChEMBL, to benchmark the performance of multiple machine learning methods [7]. The study emulates the typical scenario of early drug discovery (lots of data on inactive compounds *vs*. almost no information on actives) and extends the comparisons to multitarget drug discovery setups, where activities are predicted simultaneously for more drug targets. Another example is Pareto-Optimal Embedded Modeling (POEM), a non-parametric, supervised machine learning algorithm developed to generate reliable predictive models without need for optimization. POEM’s predictive strength is obtained by combining multiple different representations of molecular structures [21].

In this study we propose a novel alternative to pairwise similarity calculations. Instead of using multiple binary comparisons to analyze a dataset, we present multiple classes of comparative measures that can be used to compare an arbitrary number of molecules at the same time. The central element of our work is to provide a general framework for comparing multiple objects at the same time, which naturally extends the range of validity of most of the similarity indices commonly used in cheminformatics and drug design. This was based on a comprehensive analysis of the binary similarity measures defined so far, which allowed us to identify their fundamental defining features (*e.g*., similarity/dissimilarity counters, coincidence thresholds), which are the key to defining fully general *n*-ary similarity indices. We performed variance analyses in order to decompose the effects of various factors: number of molecules compared simultaneously, effect of weighting, types of similarity coefficients, and length of the fingerprint. These new families of indices considerably expand the scope of the comparative analysis since they provide new dimensions to what is currently achievable with standard binary comparisons. Moreover, beyond their intrinsic theoretical interest, we anticipate that *n*-ary comparisons can have important practical applications ranging from estimating set-similarity to providing new rigorous ways to study chemical diversity and explore compound databases. In particular, we have found that calculating the introduced *n*-ary comparisons for large datasets is excessively faster than the traditional approach of calculating full pairwise similarity matrices to quantify the diversity of a compound set. After introducing the theoretical basis of the *n*-ary fingerprint comparisons here, we share our detailed results on the practical applicability of this framework in the accompanying paper [(part 2) 22]. Meanwhile, the Python code for calculating the extended similarity metrics is freely available at https://github.com/ramirandaq/MultipleComparisons.

## Theory of fingerprint comparisons

### Binary comparisons

Similarity measures/indices are generally presented as binary relations, in the sense that they assign a (real) number to a pair of molecules. These relations are based on a suitable representation of the molecules, either in terms of graphs, lexicographical tools (like the SMARTS or SMILES formats), field-based quantities (like the electron density or the molecular electrostatic potential), or the widely used molecular fingerprints. Here, we will focus on the latter, particularly on the well-known binary fingerprints, where a molecule is represented as a string of 1′s and 0′s (without restricting the scope of our approach).

It is important to point out that the word “binary” has two meanings in our context. On the level of fingerprints, it means that a fingerprint consists of a string of two possible values (0 and 1). Let us call such strings as dichotomous variables further on. On the level of comparisons, it means that two objects (molecules, fingerprints) are compared simultaneously. Since in the present work, we exclusively apply binary (dichotomous) fingerprints, our use of the word “binary” will refer to comparisons of two objects/molecules, in contrast to the simultaneous comparison of multiple objects (“*n*-ary comparisons”), which is the central concept of this study.

In the case of the binary comparison of (dichotomous) fingerprints, there are four basic quantities that we can calculate for each pair of fingerprints:

*a*: the number of coincident 1′s (number of common *on* bits).

*b*: number of 1′s present in the first fingerprint but absent in the second.

*c*: number of 1′s present in the second fingerprint but absent in the first.

*d*: the number of coincident 0′s (number of common *off* bits).

For instance, in the following example:

1 $$\begin{array}{c}A=\left(1 0 1 1 0 1 0 0\right)\\ B=\left(0 0 1 0 0 1 0 1\right)\end{array}$$

*a* = 2, *b* = 2, *c* = 1, *d* = 3.

These numbers can then be combined in many different ways in order to define multiple similarity indices (for a comprehensive list see the Additional file 1). In general, the similarity indices have the following form:

2 $$s={\sum }_{i}{G}_{i}\left(\frac{{g}_{i1}\left(a,d,b,c\right)}{{g}_{i2}\left(a,d,b,c\right)}\right)$$

where *G*_*i*_, *g*_*i1*_ and *g*_*i2*_ represent functions in general. For example, the Kulczynski (Kul) index is given by:

3 $${s}_{Kul}=\frac{1}{2}\left(\frac{a}{a+b}+\frac{a}{a+c}\right)$$

Nonetheless, there are other indices (which we will call *additive*) for which we only need the sum of *b* and *c*, namely:

4 $$s={\sum }_{i}{G}_{i}\left(\frac{{g}_{i1}\left(a,d,b+c\right)}{{g}_{i2}\left(a,d,b+c\right)}\right)$$

like the Sokal-Michener (SM) index:

5 $${s}_{SM}=\frac{a+d}{p}$$

where *p* = *a* + *b* + *c* + *d*.

Finally, within the additive indices we encounter a sub-class of indices that we will call *asymmetric*, because they depend on *a*, but not on *d*, that is:

6 $$s={\sum }_{i}{G}_{i}\left(\frac{{g}_{i1}\left(a,b+c\right)}{{g}_{i2}\left(a,b+c\right)}\right)$$

a representative example of this class would be the Jaccard-Tanimoto (JT) index (widely known as Tanimoto similarity in the cheminformatics and drug discovery communities):

7 $${s}_{JT}=\frac{a}{a+b+c}$$

### *n*-ary comparisons

In the effort to extend the expressions of the binary comparisons in order to simultaneously compare an arbitrary number *n* of molecular fingerprints (*n*-ary comparisons), the first step is to introduce the notation ${C}_{n(k)}$ to represent the number of times that we find *k* coinciding 1′s between *n* bitstrings (irrespective of the order in which we consider the fingerprints). (Notice that $0\le k\le n$.) For instance, in the binary (*n* = 2) case: ${C}_{2(2)}=a$, ${C}_{2(1)}=b+c$, ${C}_{2(0)}=d$. Obviously, with this simple notation we can only discuss additive indices for now (see Eq. 4). We defer to a future work a discussion of *n*-ary comparisons applicable to general indices Eq. (2).

The key detail that we need to notice is that, in the case of binary comparisons, pairs of fingerprints with large values of *a* and *d* will be more similar, and pairs of fingerprints with large values of *b* and *c* will be less similar. Then, it makes sense to classify *a* and *d* as *similarity counters* (in particular, *a* will be a 1-similarity counter and *d* a 0-similarity counter), and *b* and *c* as *dissimilarity counters*. Extending this notion to *n*-ary comparisons requires us to classify ${C}_{n(k)}$ as similarity and dissimilarity counters as well. We do this with the help of the following indicator:

8 $${\Delta }_{n(k)}=|2k-n|$$

It is clear that a bigger value of ${\Delta }_{n(k)}$ will imply that the given strings have more elements in common (either 1′s or 0′s). Now we must define a minimum value that determines from what point a given number of occurrences can be considered as *coincident*. We will denote this *coincidence threshold* as *γ*. There are many possible ways to define this threshold, for instance, a somehow contrived possibility would be to set $\gamma = \left\lceil {\frac{n}{2}} \right\rceil$ (where $\left\lceil x \right\rceil$ is the ceiling function). However, perhaps a better option (in the sense that it maximizes the number of similarity counters) will be to take $\gamma =n\text{mod}2$. (Roughly speaking, this choice of *γ* means that the fingerprints will be “similar” at a given position if more than half of the bits have the same value in that position.) In any case, we will take ${C}_{n(k)}$ as a similarity counter if ${\Delta }_{n(k)}>\gamma$, and as a dissimilarity counter when ${\Delta }_{n(k)}\le \gamma$. In particular, ${C}_{n(k)}$ will be a 1-similarity counter if $2k-n>\gamma$, and a 0-similarity counter if $n-2k>\gamma$. Notice that, as expected for the *n* = 2 case, ${C}_{2(2)}$ and ${C}_{2(0)}$ will be 1- and 0-similarity counters, respectively, while ${C}_{2(1)}$ will be a dissimilarity counter.

Finally, we should discuss the relative relevance that distinct types of similarity and dissimilarity counters have within a given similarity index. For instance, let us consider *n* = 4 and set $\gamma =0$. In this case, both ${C}_{4(4)}$ and ${C}_{4(3)}$ will be 1-similarity counters, but the first one indicates when we have a 100% coincidence among the compared fingerprints, while the latter indicates when we have a 75% concordance among the compared fingerprints. Therefore, it seems natural to weight these counters differently. We can do this according to the following convention: If ${C}_{n(k)}$ is a similarity counter, then it should be multiplied by a factor ${f}_{s}\left({\Delta }_{n(k)}\right)$ that is an increasing function of ${\Delta }_{n(k)}$. Contrary, if ${C}_{n(k)}$ is a dissimilarity counter, it should be multiplied by a factor ${f}_{d}\left({\Delta }_{n(k)}\right)$ that is a decreasing function of ${\Delta }_{n(k)}$. In both cases we must have: ${f}_{s}\left(n\right)={f}_{d}\left(n\text{mod}2\right)=1$. As it was the case for $\gamma$, there are many ways of choosing *f*_*s*_ and *f*_*d*_. One possibility would be to set ${f}_{s}\left({\Delta }_{n(k)}\right)={2}^{-\left(n-{\Delta }_{n(k)}\right)}$ and ${f}_{d}\left({\Delta }_{n(k)}\right)={2}^{-\left({\Delta }_{n\left(k\right)}-n\text{mod}2\right)}$. However, this might put too harsh a penalty on the different counters. For this reason, in the following we will use instead these weight functions:

9 $${f}_{s}\left({\Delta }_{n(k)}\right)=\frac{{\Delta }_{n(k)}}{n}, {f}_{d}\left({\Delta }_{n(k)}\right)=1-\frac{{\Delta }_{n(k)}-n\text{mod}2}{n}$$

At this point we have all the necessary ingredients to generalize the binary comparisons. An additive index like the one presented in Eq. (4) can now be rewritten as:

10 $${s}_{1s\_wd}={\sum }_{i}{G}_{i}\left(\frac{{g}_{i1}\left(\sum_{1-s}{f}_{s}\left({\Delta }_{n(k)}\right){C}_{n(k)},\sum_{0-s}{f}_{s}\left({\Delta }_{n(k)}\right){C}_{n(k)},\sum_{d}{f}_{d}\left({\Delta }_{n(k)}\right){C}_{n(k)}\right)}{{g}_{i2}\left(\sum_{1-s}{f}_{s}\left({\Delta }_{n(k)}\right){C}_{n(k)},\sum_{0-s}{f}_{s}\left({\Delta }_{n(k)}\right){C}_{n(k)},\sum_{d}{f}_{d}\left({\Delta }_{n(k)}\right){C}_{n(k)}\right)}\right)$$

Let us briefly explain the notation in the previous expression: the summations over 1-*s*, 0-*s*, and *d* represent the sum over the 1-similarity, 0-similarity, and dissimilarity counters, respectively. The subscript 1s_wd indicates that we are distinguishing between the 1- and 0-similarity counters (hence the “1” in the similarity “s” part), and that the counters in the denominator are weighted (hence the “*w*” in the denominator “*d*” part). We introduce this distinction because we can propose yet another generalization for the additive indices, in the form of:

11 $${s}_{1s\_d}={\sum }_{i}{G}_{i}\left(\frac{{g}_{i1}\left(\sum_{1-s}{f}_{s}\left({\Delta }_{n(k)}\right){C}_{n(k)},\sum_{0-s}{f}_{s}\left({\Delta }_{n(k)}\right){C}_{n(k)},\sum_{d}{f}_{d}\left({\Delta }_{n(k)}\right){C}_{n(k)}\right)}{{g}_{i2}\left(\sum_{1-s}{C}_{n(k)},\sum_{0-s}{C}_{n(k)},\sum_{d}{C}_{n(k)}\right)}\right)$$

Notice that now we are *not weighting* the counters in the denominator (which is reflected in the subscript 1s_d).

As an example of these two possible extensions, let us once again revisit the SM index (detailed expressions for the remaining additive indices are given in Appendix 1: Table 1):

**Table 1:** Extended *n*-ary similarity indices

Additive indices				
Label	Type	Notation	Name	Equation
eAC	eAC_1	eACw	extended Austin-Colwell	${s}_{eAC\left(1s\_wd\right)}=\frac{2}{\pi }\text{arcsin}\sqrt{\frac{\sum_{1-s}{f}_{s}\left({\Delta }_{n(k)}\right){C}_{n(k)}+\sum_{0-s}{f}_{s}\left({\Delta }_{n(k)}\right){C}_{n(k)}}{\sum_{s}{f}_{s}\left({\Delta }_{n(k)}\right){C}_{n(k)}+\sum_{d}{f}_{d}\left({\Delta }_{n(k)}\right){C}_{n(k)}}}$
		eACnw		${s}_{eAC\left(1s\_d\right)}=\frac{2}{\pi }\text{arcsin}\sqrt{\frac{\sum_{1-s}{f}_{s}\left({\Delta }_{n(k)}\right){C}_{n(k)}+\sum_{0-s}{f}_{s}\left({\Delta }_{n(k)}\right){C}_{n(k)}}{\sum_{s}{C}_{n(k)}+\sum_{d}{C}_{n(k)}}}$
eBUB	eBUB_1	eBUBw	extended Baroni-Urbani-Buser	$${s}_{eBUB\left(1s\_wd\right)}=\frac{\sqrt{\left[\sum_{1-s}{f}_{s}\left({\Delta }_{n(k)}\right){C}_{n(k)}\right]\left[\sum_{0-s}{f}_{s}\left({\Delta }_{n(k)}\right){C}_{n(k)}\right]}+\sum_{1-s}{f}_{s}\left({\Delta }_{n(k)}\right){C}_{n(k)}}{\left\{\begin{array}{c}\sqrt{\left[\sum_{1-s}{f}_{s}\left({\Delta }_{n\left(k\right)}\right){C}_{n\left(k\right)}\right]\left[\sum_{0-s}{f}_{s}\left({\Delta }_{n\left(k\right)}\right){C}_{n\left(k\right)}\right]}+\\ \sum_{1-s}{f}_{s}\left({\Delta }_{n\left(k\right)}\right){C}_{n\left(k\right)}+\sum_{d}{f}_{d}\left({\Delta }_{n\left(k\right)}\right){C}_{n\left(k\right)}\end{array}\right\}}$$
		eBUBnw		${s}_{eBUB\left(1s\_d\right)}=\frac{\sqrt{\left[\sum_{1-s}{f}_{s}\left({\Delta }_{n(k)}\right){C}_{n(k)}\right]\left[\sum_{0-s}{f}_{s}\left({\Delta }_{n(k)}\right){C}_{n(k)}\right]}+\sum_{1-s}{f}_{s}\left({\Delta }_{n(k)}\right){C}_{n(k)}}{\left\{\sqrt{\left[\sum_{1-s}{C}_{n\left(k\right)}\right]\left[\sum_{0-s}{C}_{n\left(k\right)}\right]}+\sum_{1-s}{C}_{n\left(k\right)}+\sum_{d}{C}_{n\left(k\right)}\right\}}$
eCT1	eCT1_1	eCT1w	extended Consoni-Todeschini (1)	${s}_{eCT1\left(1s\_wd\right)}=\frac{\text{ln}\left(1+\sum_{1-s}{f}_{s}\left({\Delta }_{n(k)}\right){C}_{n(k)}+\sum_{0-s}{f}_{s}\left({\Delta }_{n(k)}\right){C}_{n(k)}\right)}{\text{ln}\left(1+\sum_{s}{f}_{s}\left({\Delta }_{n(k)}\right){C}_{n(k)}+\sum_{d}{f}_{d}\left({\Delta }_{n(k)}\right){C}_{n(k)}\right)}$
		eCT1nw		${s}_{eCT1\left(1s\_d\right)}=\frac{\text{ln}\left(1+\sum_{1-s}{f}_{s}\left({\Delta }_{n(k)}\right){C}_{n(k)}+\sum_{0-s}{f}_{s}\left({\Delta }_{n(k)}\right){C}_{n(k)}\right)}{\text{ln}\left(1+\sum_{s}{C}_{n(k)}+\sum_{d}{C}_{n(k)}\right)}$
eCT2	eCT2_1	eCT2w	extended Consoni-Todeschini (2)	${s}_{eCT2\left(1s\_wd\right)}=\frac{\text{ln}\left(1+\sum_{s}{f}_{s}\left({\Delta }_{n(k)}\right){C}_{n(k)}+\sum_{d}{f}_{d}\left({\Delta }_{n(k)}\right){C}_{n(k)}\right)-\text{ln}\left(1+\sum_{d}{f}_{d}\left({\Delta }_{n(k)}\right){C}_{n(k)}\right)}{\text{ln}\left(1+\sum_{s}{f}_{s}\left({\Delta }_{n(k)}\right){C}_{n(k)}+\sum_{d}{f}_{d}\left({\Delta }_{n(k)}\right){C}_{n(k)}\right)}$
		eCT2nw		${s}_{eCT2\left(1s\_d\right)}=\frac{\text{ln}\left(1+\sum_{s}{f}_{s}\left({\Delta }_{n(k)}\right){C}_{n(k)}+\sum_{d}{f}_{d}\left({\Delta }_{n(k)}\right){C}_{n(k)}\right)-\text{ln}\left(1+\sum_{d}{f}_{d}\left({\Delta }_{n(k)}\right){C}_{n(k)}\right)}{\text{ln}\left(1+\sum_{s}{C}_{n(k)}+\sum_{d}{C}_{n(k)}\right)}$
eFai	eFai_1	eFaiw	extended Faith	${s}_{eFai\left(1s\_wd\right)}=\frac{\sum_{1-s}{f}_{s}\left({\Delta }_{n(k)}\right){C}_{n(k)}+0.5\sum_{0-s}{f}_{s}\left({\Delta }_{n(k)}\right){C}_{n(k)}}{\sum_{s}{f}_{s}\left({\Delta }_{n(k)}\right){C}_{n(k)}+\sum_{d}{f}_{d}\left({\Delta }_{n(k)}\right){C}_{n(k)}}$
		eFainw		${s}_{eFai\left(1s\_d\right)}=\frac{\sum_{1-s}{f}_{s}\left({\Delta }_{n(k)}\right){C}_{n(k)}+0.5\sum_{0-s}{f}_{s}\left({\Delta }_{n(k)}\right){C}_{n(k)}}{\sum_{s}{C}_{n(k)}+\sum_{d}{C}_{n(k)}}$
eGK	eGK_1	eGKw	extended Goodman–Kruskal	${s}_{eGK\left(1s\_wd\right)}=\frac{2\text{min}\left(\sum_{1-s}{f}_{s}\left({\Delta }_{n(k)}\right){C}_{n(k)},\sum_{0-s}{f}_{s}\left({\Delta }_{n(k)}\right){C}_{n(k)}\right)-\sum_{d}{f}_{d}\left({\Delta }_{n(k)}\right){C}_{n(k)}}{2\text{min}\left(\sum_{1-s}{f}_{s}\left({\Delta }_{n(k)}\right){C}_{n(k)},\sum_{0-s}{f}_{s}\left({\Delta }_{n(k)}\right){C}_{n(k)}\right)+\sum_{d}{f}_{d}\left({\Delta }_{n(k)}\right){C}_{n(k)}}$
		eGKnw		${s}_{eGK\left(1s\_d\right)}=\frac{2\text{min}\left(\sum_{1-s}{f}_{s}\left({\Delta }_{n(k)}\right){C}_{n(k)},\sum_{0-s}{f}_{s}\left({\Delta }_{n(k)}\right){C}_{n(k)}\right)-\sum_{d}{f}_{d}\left({\Delta }_{n(k)}\right){C}_{n(k)}}{2\text{min}\left(\sum_{1-s}{C}_{n(k)},\sum_{0-s}{C}_{n(k)}\right)+\sum_{d}{C}_{n(k)}}$
eHD	eHD_1	eHDw	extended Hawkins-Dotson	$${s}_{eHD\left(1s\_wd\right)}=\frac{1}{2}\left(\begin{array}{c}\frac{\sum_{1-s}{f}_{s}\left({\Delta }_{n(k)}\right){C}_{n(k)}}{\sum_{1-s}{f}_{s}\left({\Delta }_{n(k)}\right){C}_{n(k)}+\sum_{d}{f}_{d}\left({\Delta }_{n(k)}\right){C}_{n(k)}}+\\ \frac{\sum_{0-s}{f}_{s}\left({\Delta }_{n(k)}\right){C}_{n(k)}}{\sum_{0-s}{f}_{s}\left({\Delta }_{n(k)}\right){C}_{n(k)}+\sum_{d}{f}_{d}\left({\Delta }_{n(k)}\right){C}_{n(k)}}\end{array}\right)$$
		eHDnw		$${s}_{eHD\left(1s\_d\right)}=\frac{1}{2}\left(\begin{array}{c}\frac{\sum_{1-s}{f}_{s}\left({\Delta }_{n(k)}\right){C}_{n(k)}}{\sum_{1-s}{C}_{n(k)}+\sum_{d}{C}_{n(k)}}+\\ \frac{\sum_{0-s}{f}_{s}\left({\Delta }_{n(k)}\right){C}_{n(k)}}{\sum_{0-s}{C}_{n(k)}+\sum_{d}{C}_{n(k)}}\end{array}\right)$$
eRT	eRT_1	eRTw	extended Rogers-Tanimoto	${s}_{eRT\left(1s\_wd\right)}=\frac{\sum_{s}{f}_{s}\left({\Delta }_{n(k)}\right){C}_{n(k)}}{\sum_{s}{f}_{s}\left({\Delta }_{n(k)}\right){C}_{n(k)}+2\sum_{d}{f}_{d}\left({\Delta }_{n(k)}\right){C}_{n(k)}}$
		eRTnw		${s}_{eRT\left(1s\_d\right)}=\frac{\sum_{s}{f}_{s}\left({\Delta }_{n(k)}\right){C}_{n(k)}}{\sum_{s}{C}_{n(k)}+2\sum_{d}{C}_{n(k)}}$
eRG	eRG_1	eRGw	extended Rogot-Goldberg	$${s}_{eRG\left(1s\_wd\right)}=\begin{array}{c}\frac{\sum_{1-s}{f}_{s}\left({\Delta }_{n(k)}\right){C}_{n(k)}}{2\sum_{1-s}{f}_{s}\left({\Delta }_{n(k)}\right){C}_{n(k)}+\sum_{d}{f}_{d}\left({\Delta }_{n(k)}\right){C}_{n(k)}}+\\ \frac{\sum_{0-s}{f}_{s}\left({\Delta }_{n(k)}\right){C}_{n(k)}}{2\sum_{0-s}{f}_{s}\left({\Delta }_{n(k)}\right){C}_{n(k)}+\sum_{d}{f}_{d}\left({\Delta }_{n(k)}\right){C}_{n(k)}}\end{array}$$
		eRGnw		${s}_{eRG\left(1s\_d\right)}=\frac{\sum_{1-s}{f}_{s}\left({\Delta }_{n(k)}\right){C}_{n(k)}}{2\sum_{1-s}{C}_{n(k)}+\sum_{d}{C}_{n(k)}}+\frac{\sum_{0-s}{f}_{s}\left({\Delta }_{n(k)}\right){C}_{n(k)}}{2\sum_{0-s}{C}_{n(k)}+\sum_{d}{C}_{n(k)}}$
eSM	eSM_1	eSMw	extended Simple matching, Sokal-Michener	${s}_{eSM\left(1s\_wd\right)}=\frac{\sum_{s}{f}_{s}\left({\Delta }_{n(k)}\right){C}_{n(k)}}{\sum_{s}{f}_{s}\left({\Delta }_{n(k)}\right){C}_{n(k)}+\sum_{d}{f}_{d}\left({\Delta }_{n(k)}\right){C}_{n(k)}}$
		eSMnw		${s}_{eSM\left(1s\_d\right)}=\frac{\sum_{s}{f}_{s}\left({\Delta }_{n(k)}\right){C}_{n(k)}}{\sum_{s}{C}_{n(k)}+\sum_{d}{C}_{n(k)}}$
eSS2	eSS2_1	eSS2w	extended Sokal-Sneath (2)	${s}_{eSS2\left(1s\_wd\right)}=\frac{2\sum_{s}{f}_{s}\left({\Delta }_{n(k)}\right){C}_{n(k)}}{2\sum_{s}{f}_{s}\left({\Delta }_{n(k)}\right){C}_{n(k)}+\sum_{d}{f}_{d}\left({\Delta }_{n(k)}\right){C}_{n(k)}}$
		eSS2nw		${s}_{eSS2\left(1s\_wd\right)}=\frac{2\sum_{s}{f}_{s}\left({\Delta }_{n(k)}\right){C}_{n(k)}}{2\sum_{s}{C}_{n(k)}+\sum_{d}{C}_{n(k)}}$

Asymmetric indices				
Label	Type	Notation	Name	Equation
eCT3	eCT3_1	eCT3w	extended Consoni-Todeschini (3)	${s}_{eCT3\left(1s\_wd\right)}=\frac{\text{ln}\left(1+\sum_{1-s}{f}_{s}\left({\Delta }_{n(k)}\right){C}_{n(k)}\right)}{\text{ln}\left(1+\sum_{s}{f}_{s}\left({\Delta }_{n(k)}\right){C}_{n(k)}+\sum_{d}{f}_{d}\left({\Delta }_{n(k)}\right){C}_{n(k)}\right)}$
		eCT3nw		${s}_{eCT3\left(1s\_d\right)}=\frac{\text{ln}\left(1+\sum_{1-s}{f}_{s}\left({\Delta }_{n(k)}\right){C}_{n(k)}\right)}{\text{ln}\left(1+\sum_{s}{C}_{n(k)}+\sum_{d}{C}_{n(k)}\right)}$
	eCT3_0	eCT30w		${s}_{eCT3\left(s\_wd\right)}=\frac{\text{ln}\left(1+\sum_{s}{f}_{s}\left({\Delta }_{n(k)}\right){C}_{n(k)}\right)}{\text{ln}\left(1+\sum_{s}{f}_{s}\left({\Delta }_{n(k)}\right){C}_{n(k)}+\sum_{d}{f}_{d}\left({\Delta }_{n(k)}\right){C}_{n(k)}\right)}$
		eCT30nw		${s}_{eCT3\left(s\_d\right)}=\frac{\text{ln}\left(1+\sum_{s}{f}_{s}\left({\Delta }_{n(k)}\right){C}_{n(k)}\right)}{\text{ln}\left(1+\sum_{s}{C}_{n(k)}+\sum_{d}{C}_{n(k)}\right)}$
eCT4	eCT4_1	eCT4w	extended Consoni-Todeschini (4)	${s}_{eCT4\left(1s\_wd\right)}=\frac{\text{ln}\left(1+\sum_{1-s}{f}_{s}\left({\Delta }_{n(k)}\right){C}_{n(k)}\right)}{\text{ln}\left(1+\sum_{1-s}{f}_{s}\left({\Delta }_{n(k)}\right){C}_{n(k)}+\sum_{d}{f}_{d}\left({\Delta }_{n(k)}\right){C}_{n(k)}\right)}$
		eCT4nw		${s}_{eCT4\left(1s\_d\right)}=\frac{\text{ln}\left(1+\sum_{1-s}{f}_{s}\left({\Delta }_{n(k)}\right){C}_{n(k)}\right)}{\text{ln}\left(1+\sum_{1-s}{C}_{n(k)}+\sum_{d}{C}_{n(k)}\right)}$
	eCT4_0	eCT40w		${s}_{eCT4\left(s\_wd\right)}=\frac{\text{ln}\left(1+\sum_{s}{f}_{s}\left({\Delta }_{n(k)}\right){C}_{n(k)}\right)}{\text{ln}\left(1+\sum_{s}{f}_{s}\left({\Delta }_{n(k)}\right){C}_{n(k)}+\sum_{d}{f}_{d}\left({\Delta }_{n(k)}\right){C}_{n(k)}\right)}$
		eCT4nw		${s}_{eCT4\left(s\_d\right)}=\frac{\text{ln}\left(1+\sum_{s}{f}_{s}\left({\Delta }_{n(k)}\right){C}_{n(k)}\right)}{\text{ln}\left(1+\sum_{s}{C}_{n(k)}+\sum_{d}{C}_{n(k)}\right)}$
eGle	eGle_1	eGlew	extended Gleason	${s}_{eGle\left(1s\_wd\right)}=\frac{2\sum_{1-s}{f}_{s}\left({\Delta }_{n(k)}\right){C}_{n(k)}}{2\sum_{1-s}{f}_{s}\left({\Delta }_{n(k)}\right){C}_{n(k)}+\sum_{d}{f}_{d}\left({\Delta }_{n(k)}\right){C}_{n(k)}}$
		eGlenw		${s}_{eGle\left(1s\_d\right)}=\frac{2\sum_{1-s}{f}_{s}\left({\Delta }_{n(k)}\right){C}_{n(k)}}{2\sum_{1-s}{C}_{n(k)}+\sum_{d}{C}_{n(k)}}$
	eGle_0	eGle0w		${s}_{eGle\left(s\_wd\right)}=\frac{2\sum_{s}{f}_{s}\left({\Delta }_{n(k)}\right){C}_{n(k)}}{2\sum_{s}{f}_{s}\left({\Delta }_{n(k)}\right){C}_{n(k)}+\sum_{d}{f}_{d}\left({\Delta }_{n(k)}\right){C}_{n(k)}}$
		eGle0nw		${s}_{eGle\left(s\_d\right)}=\frac{2\sum_{s}{f}_{s}\left({\Delta }_{n(k)}\right){C}_{n(k)}}{2\sum_{s}{C}_{n(k)}+\sum_{d}{C}_{n(k)}}$
eJa	eJa_1	eJaw	extended Jaccard	${s}_{eJa\left(1s\_wd\right)}=\frac{3\sum_{1-s}{f}_{s}\left({\Delta }_{n(k)}\right){C}_{n(k)}}{3\sum_{1-s}{f}_{s}\left({\Delta }_{n(k)}\right){C}_{n(k)}+\sum_{d}{f}_{d}\left({\Delta }_{n(k)}\right){C}_{n(k)}}$
		eJanw		${s}_{eJa\left(1s\_d\right)}=\frac{3\sum_{1-s}{f}_{s}\left({\Delta }_{n(k)}\right){C}_{n(k)}}{3\sum_{1-s}{C}_{n(k)}+\sum_{d}{C}_{n(k)}}$
	eJa_0	eJa0w		${s}_{eJa\left(s\_wd\right)}=\frac{3\sum_{s}{f}_{s}\left({\Delta }_{n(k)}\right){C}_{n(k)}}{3\sum_{s}{f}_{s}\left({\Delta }_{n(k)}\right){C}_{n(k)}+\sum_{d}{f}_{d}\left({\Delta }_{n(k)}\right){C}_{n(k)}}$
		eJa0nw		${s}_{eJa\left(s\_d\right)}=\frac{3\sum_{s}{f}_{s}\left({\Delta }_{n(k)}\right){C}_{n(k)}}{3\sum_{s}{C}_{n(k)}+\sum_{d}{C}_{n(k)}}$
eRR	eRR_1	eRRw	extended Russel-Rao	${s}_{eRR\left(1s\_wd\right)}=\frac{\sum_{1-s}{f}_{s}\left({\Delta }_{n(k)}\right){C}_{n(k)}}{\sum_{s}{f}_{s}\left({\Delta }_{n(k)}\right){C}_{n(k)}+\sum_{d}{f}_{d}\left({\Delta }_{n(k)}\right){C}_{n(k)}}$
		eRRnw		${s}_{eRR\left(1s\_d\right)}=\frac{\sum_{1-s}{f}_{s}\left({\Delta }_{n(k)}\right){C}_{n(k)}}{\sum_{s}{C}_{n(k)}+\sum_{d}{C}_{n(k)}}$
	eRR_0	eRR0w		${s}_{eRR\left(s\_wd\right)}=\frac{\sum_{s}{f}_{s}\left({\Delta }_{n(k)}\right){C}_{n(k)}}{\sum_{s}{f}_{s}\left({\Delta }_{n(k)}\right){C}_{n(k)}+\sum_{d}{f}_{d}\left({\Delta }_{n(k)}\right){C}_{n(k)}}$
		eRR0nw		${s}_{eRR\left(s\_d\right)}=\frac{\sum_{s}{f}_{s}\left({\Delta }_{n(k)}\right){C}_{n(k)}}{\sum_{s}{C}_{n(k)}+\sum_{d}{C}_{n(k)}}$
eSS1	eSS1_0	eSSw	extended Sokal-Sneath (1)	${s}_{eSS1\left(1s\_wd\right)}=\frac{\sum_{1-s}{f}_{s}\left({\Delta }_{n(k)}\right){C}_{n(k)}}{\sum_{1-s}{f}_{s}\left({\Delta }_{n(k)}\right){C}_{n(k)}+2\sum_{d}{f}_{d}\left({\Delta }_{n(k)}\right){C}_{n(k)}}$
		eSSnw		${s}_{eSS1\left(1s\_d\right)}=\frac{\sum_{1-s}{f}_{s}\left({\Delta }_{n(k)}\right){C}_{n(k)}}{\sum_{1-s}{C}_{n(k)}+2\sum_{d}{C}_{n(k)}}$
	eSS1_1	eSS0w		${s}_{eSS1\left(s\_wd\right)}=\frac{\sum_{s}{f}_{s}\left({\Delta }_{n(k)}\right){C}_{n(k)}}{\sum_{s}{f}_{s}\left({\Delta }_{n(k)}\right){C}_{n(k)}+2\sum_{d}{f}_{d}\left({\Delta }_{n(k)}\right){C}_{n(k)}}$
		eSS0nw		${s}_{eSS1\left(s\_d\right)}=\frac{\sum_{s}{f}_{s}\left({\Delta }_{n(k)}\right){C}_{n(k)}}{\sum_{s}{C}_{n(k)}+2\sum_{d}{C}_{n(k)}}$
eJT	eJT_1	eJTw	extended Jaccard-Tanimoto	${s}_{eJT\left(1s\_wd\right)}=\frac{\sum_{1-s}{f}_{s}\left({\Delta }_{n(k)}\right){C}_{n(k)}}{\sum_{1-s}{f}_{s}\left({\Delta }_{n(k)}\right){C}_{n(k)}+\sum_{d}{f}_{d}\left({\Delta }_{n(k)}\right){C}_{n(k)}}$
		eJTnw		${s}_{eJT\left(1s\_d\right)}=\frac{\sum_{1-s}{f}_{s}\left({\Delta }_{n(k)}\right){C}_{n(k)}}{\sum_{1-s}{C}_{n(k)}+\sum_{d}{C}_{n(k)}}$
	eJT_0	eJT0w		${s}_{eJT\left(s\_wd\right)}=\frac{\sum_{s}{f}_{s}\left({\Delta }_{n(k)}\right){C}_{n(k)}}{\sum_{s}{f}_{s}\left({\Delta }_{n(k)}\right){C}_{n(k)}+\sum_{d}{f}_{d}\left({\Delta }_{n(k)}\right){C}_{n(k)}}$
		eJT0nw		${s}_{eJT\left(s\_d\right)}=\frac{\sum_{s}{f}_{s}\left({\Delta }_{n(k)}\right){C}_{n(k)}}{\sum_{s}{C}_{n(k)}+\sum_{d}{C}_{n(k)}}$

12 $${s}_{eSM\left(1s\_wd\right)}=\frac{\sum_{1-s}{f}_{s}\left({\Delta }_{n(k)}\right){C}_{n(k)}+\sum_{0-s}{f}_{s}\left({\Delta }_{n(k)}\right){C}_{n(k)}}{\sum_{1-s}{f}_{s}\left({\Delta }_{n(k)}\right){C}_{n(k)}+\sum_{0-s}{f}_{s}\left({\Delta }_{n(k)}\right){C}_{n(k)}+\sum_{d}{f}_{d}\left({\Delta }_{n(k)}\right){C}_{n(k)}}$$

13 $${s}_{eSM\left(1s\_d\right)}=\frac{\sum_{1-s}{f}_{s}\left({\Delta }_{n(k)}\right){C}_{n(k)}+\sum_{0-s}{f}_{s}\left({\Delta }_{n(k)}\right){C}_{n(k)}}{\sum_{1-s}{C}_{n(k)}+\sum_{0-s}{C}_{n(k)}+\sum_{d}{C}_{n(k)}}$$

Here and in the following we will distinguish the extended (*n*-ary) versions of the similarity indices by including an “e” as a subscript (notice the difference with respect to Eq. (5).

Since the asymmetric indices are a sub-class of the additive indices they can also be extended in this form, namely:

14 $${s}_{1s\_wd}={\sum }_{i}{G}_{i}\left(\frac{{g}_{i1}\left(\sum_{1-s}{f}_{s}\left({\Delta }_{n(k)}\right){C}_{n(k)},\sum_{d}{f}_{d}\left({\Delta }_{n(k)}\right){C}_{n(k)}\right)}{{g}_{i2}\left(\sum_{1-s}{f}_{s}\left({\Delta }_{n(k)}\right){C}_{n(k)},\sum_{d}{f}_{d}\left({\Delta }_{n(k)}\right){C}_{n(k)}\right)}\right)$$

15 $${s}_{1s\_d}={\sum }_{i}{G}_{i}\left(\frac{{g}_{i1}\left(\sum_{1-s}{f}_{s}\left({\Delta }_{n(k)}\right){C}_{n(k)},\sum_{d}{f}_{d}\left({\Delta }_{n(k)}\right){C}_{n(k)}\right)}{{g}_{i2}\left(\sum_{1-s}{C}_{n(k)},\sum_{d}{C}_{n(k)}\right)}\right)$$

Moreover, there are further possibilities, if we replace the sum over the 1-similarity counters with a sum over all the similarity counters:

16 $${s}_{s\_wd}={\sum }_{i}{G}_{i}\left(\frac{{g}_{i1}\left(\sum_{s}{f}_{s}\left({\Delta }_{n(k)}\right){C}_{n(k)},\sum_{d}{f}_{d}\left({\Delta }_{n(k)}\right){C}_{n(k)}\right)}{{g}_{i2}\left(\sum_{s}{f}_{s}\left({\Delta }_{n(k)}\right){C}_{n(k)},\sum_{d}{f}_{d}\left({\Delta }_{n(k)}\right){C}_{n(k)}\right)}\right)$$

17 $${s}_{s\_d}={\sum }_{i}{G}_{i}\left(\frac{{g}_{i1}\left(\sum_{s}{f}_{s}\left({\Delta }_{n(k)}\right){C}_{n(k)},\sum_{d}{f}_{d}\left({\Delta }_{n(k)}\right){C}_{n(k)}\right)}{{g}_{i2}\left(\sum_{s}{C}_{n(k)},\sum_{d}{C}_{n(k)}\right)}\right)$$

Now the summation over *s* indicates the sum over all similarity counters. Also, we do not include the “1” in the subscript because now we are not distinguishing between the 1- and 0-similarity counters. As it was the case in Eqs. (9) and (10) the presence (absence) of the “*w*” in the subscript indicates that we are (or are not) weighting the counters in the denominator. In Appendix 2 we include a detailed step-by-step calculation of the SM index for 4-ary and 5-ary comparisons.

Notice that when *n* = 2 this generalization will be equivalent to substituting *a* by *a* + *d* (and leaving *p* unchanged wherever it appears). This makes easier to realize that this procedure will be redundant in most cases, in the sense that we will just obtain the expression for an already known (additive) index. This is actually the case for six of the seven asymmetric indices considered here; Consoni-Todeschini (3) (CT3), Consoni-Todeschini (4) (CT4), Gleason (Gle), Russell-Rao (RR), Jaccard-Tanimoto (JT), and Sokal-Sneath (1) (SS1):

18 $$\begin{array}{c}{s}_{CT3}=\frac{\text{ln}\left(1+a\right)}{\text{ln}\left(1+p\right)}\\ {s}_{CT4}=\frac{\text{ln}\left(1+a\right)}{\text{ln}\left(1+a+b+c\right)}\end{array}\to {s}_{CT1}=\frac{\text{ln}\left(1+a+d\right)}{\text{ln}\left(1+p\right)}$$

19 $${s}_{Gle}=\frac{2a}{2a+b+c}\to {s}_{SS2}=\frac{2a+2d}{p+a+d}$$

20 $$\begin{array}{c}{s}_{RR}=\frac{a}{p}\\ {s}_{JT}=\frac{a}{a+b+c}\end{array}\to {s}_{SM}=\frac{a+d}{p}$$

21 $${s}_{SS1}=\frac{a}{a+2b+2c}\to {s}_{RT}=\frac{a+d}{p+b+c}$$

The Jaccard (Ja) index is the only one that actually leads to a new result:

22 $${s}_{Ja}=\frac{3a}{3a+b+c}\to {s}_{Ja0}=\frac{3a+3d}{3a+3d+b+c}=\frac{3a+3d}{p+2a+2d}$$

In the general *n*-ary case, the four possible variants of the Jaccard index are:

23 $${s}_{eJa\left(1s\_wd\right)}=\frac{3\sum_{1-s}{f}_{s}\left({\Delta }_{n(k)}\right){C}_{n(k)}}{3\sum_{1-s}{f}_{s}\left({\Delta }_{n(k)}\right){C}_{n(k)}+\sum_{d}{f}_{d}\left({\Delta }_{n(k)}\right){C}_{n(k)}}$$

24 $${s}_{eJa\left(1s\_d\right)}=\frac{3\sum_{1-s}{f}_{s}\left({\Delta }_{n(k)}\right){C}_{n(k)}}{3\sum_{1-s}{C}_{n(k)}+\sum_{d}{C}_{n(k)}}$$

25 $${s}_{eJa\left(s\_wd\right)}=\frac{3\sum_{s}{f}_{s}\left({\Delta }_{n(k)}\right){C}_{n(k)}}{3\sum_{s}{f}_{s}\left({\Delta }_{n(k)}\right){C}_{n(k)}+\sum_{d}{f}_{d}\left({\Delta }_{n(k)}\right){C}_{n(k)}}$$

26 $${s}_{eJa\left(s\_d\right)}=\frac{3\sum_{1-s}{f}_{s}\left({\Delta }_{n(k)}\right){C}_{n(k)}}{3\sum_{1-s}{C}_{n(k)}+\sum_{d}{C}_{n(k)}}$$

It is important to realize that all of these different generalizations will naturally reduce to the standard binary expressions when we substitute *n* = 2 in the above formulas.

Before concluding this section, it is worth noting that while we have focused on generalizing different similarity indices, the concepts introduced above can be used to generalize several dissimilarity indices. In Appendix 3, we briefly touch on this subject, with the particular case of the Hamming distance.

## Computational methods

### Development of sum of ranking differences (SRD)

The sum of ranking differences (SRD) algorithm was introduced in 2010, [23] showing practical examples and its validation by a permutation test. SRD was first demonstrated to solve method-comparison problems in a fast and easy way: the smaller the sum, the better the method (*i.e*. closer to the gold standard or best consensus). In the beginning, validation was done by running SRD on randomly generated variables in the size of the input data matrix. The obtained histogram shows whether the ranking is comparable with random ranking (*e.g*. when the original variables overlap with the random variables) [23]. The theoretical SRD distributions were defined for different sample sizes up to 13. The theoretical SRD can be well approximated with a Gaussian distribution, if the the number of rows (*n*) in the input matrix exceeds 13 [24]. Later, the SRD algorithm was extended to repeated observations (ties) [25]. Exact theoretical distributions were derived for 4 < *n* < 9 and a reasonable approximation works for *n* > 8, using Gaussian distribution fitted on three million *n*–dimensional random vectors [25]. Coupling SRD with variance analysis (ANOVA) provided a unique way of decomposing the effects of different factors influencing the comparions [26, 27].

Recent examinations have unambiguously shown that sum of ranking differences (SRD) realizes a multicriteria optimization [28, 29]. Lourenço and Lebensztajn have illustrated on two practical examples that SRD realizes a consensus of eight different multicriteria decision making (MCDM) methods [29], whereas any of the individual ones selects various parts of the Pareto front as optimal. Hence, the individual usage of any MCDM tools is limited; moreover, the selection of weights is a highly subjective and individual process.

Here, we will use the SRD approach to compare the extended similarity metrics with each other and study the effects of the various possible choices (*e.g.* weighting, value of *n*).

### Description of the SRD procedure

The variables or methods (here, similarity coefficents) to be compared should be arranged in the columns, and the objects (here, objects are the simulated dichotomous fingerprints) are arranged in the rows of the input matrix. The SRD technique needs a gold standard (benchmark or reference) to be defined for ranking. In lack thereof, this reference can be provided by data fusion as summarized by Willett [30] and should be selected according to the nature of the data.

The SRD algorithm consists of three stages:

1. Definition of the reference vector: Selection of a gold standard (benchmark) depending on the features of the dataset. This is straightforward, if there is a clearly defined reference vector (*e.g.* experimentally determined reference values); in other cases, data fusion from the compared data vectors is recommended. Perhaps the most frequently applied data fusion possibility is the usage of mean values. The basic assumption of their usage is that the random errors cancel each other out. The systematic errors behave similarly; provided they are numerous (above or around seven sources). Even if a (small) bias remains we are better off by using the most probable value (consensus) instead of any individual one. In fact, we have shown that the mean values as reference are inherently robust when including/omitting methods that rank the objects highly consistently, or even identically. [19] Of course other data fusion options also exist: it is “natural” to select the row minima for residuals, errors or misclassification rates. Similarly, row maximum is a suitable gold standard for the best classification rates, explained variance, etc. Recently, we have extended SRD with an option to compare each method pair-wise and present the results in a heatmap format [31].

As we have remarked before: “If the true (ideal) ranking is not known, it is expedient to substitute it with the average of all methods for each object (row average). This is the key step: the ranking by average values can be accepted as ''ideal'', since the errors cancel each other.” [23] This is precisely the approach we use here.

2. Calculation: Calculation of absolute differences between each rank-transformed individual vector values and the reference (benchmark) column (ranks, with partial rankings being used for ties), and sum the absolute differences for each object (compound) together. These values are called SRD values and rank the individual variables (similarity coefficents). SRD values are normalized between 0 and 100 to obtain comparability between various data sets. The smaller the SRD value, the closer the variable is to the benchmark (consensus). For an easy visual representation of the SRD procedure, we refer the reader to our earlier work (more specifically an animation, supplied as Additional file 3 in ref. [19]). Because the values in the columns cannot always be strictly monotonously ordered, the way of determining the index vectors' coordinates is known as ranking with ties [25].

3. Validations: a permutation test is applied as part of the validation phase, termed comparison of ranks with random numbers (CRRN). The result is shown as a cumulative frequency distribution curve in the SRD plots. Moreover, *k*-fold cross-validation was realized in two ways and the results of them were used together. A contiguous *k*-fold cross-validation and a randomized *k*-fold cross-validation (boosted repeated resampling, with return) were applied, while the number of folds can be varied (5 < *k* < 10) according to the number of samples in the original matrix [27].

Therefore, SRD is not simply a distance metric (extension of Spearman’s footrule to including repeated observations), but a multistep procedure including data fusion and validation steps [32, 33]. As SRD realizes a multicriteria (multiobjective) optimization, it selects and groups a smaller set of alternatives from the Pareto front [29].

SRD is developed as an MS Excel macro and is available for download at http://aki.ttk.mta.hu/srd.

### Factorial ANOVA

The mean of the extended similarity coefficents were analyzed using factorial analysis of variance (ANOVA) [34]. The following factors were considered: number (*n*) of objects compared (fingerprints or other representations), 14 levels: *n* = 2, 3, … 15; *m*—length of the fingerprints, four levels: *m* = 10, 100, 1000, 100 000 (fingerprints are generated as random dichotomous vectors with length *m*); role of weighting, two levels: weighted and non-weighted versions of novel similarity coefficients, and the similarity coefficient themselves, 19 levels. Factorial ANOVA was also applied for the SRD values, with the already mentioned factors (with more input data due to the validation protocols of SRD, see section “Analysis of SRD data”).

## Results

### Individual index variations

**Fig. 1:**
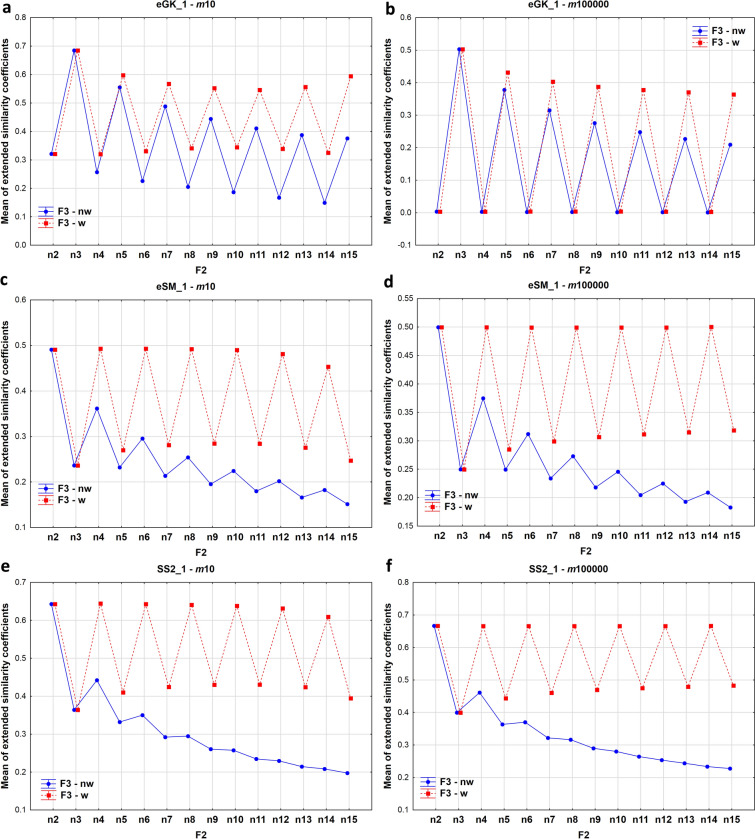
Variation of the average (of the absolute value) of all possible *n*-ary comparisons over 16 fingerprints of length *m* = 10 (**a**, **c**, **e**) and *m* = 100,000 (**b**, **d**, **f**) for different values of *n* for the extended Goodman–Kruskal (**a**, **b**), extended Sokal-Michener (**c**, **d**) and extended Sokal-Sneath (**e**, **f**) 2 indices

To explore how the introduced extended similarity metrics behave for different input data, we have generated random dichotomous fingerprints of various lengths (*m* = 10, 100, 1000 or 100,000) and calculated the extended similarity values for various numbers of compared objects (here fingerprints, *n*), according to both the weighted (w) and non-weighted (nw) formulas. In each case we randomly generated 16 fingerprints. First, let us study how the average (of the absolute value) of the comparisons for an individual index *s* (average |s|) changes when we change *n* (see Fig. 1 for some examples and the Additional file 1 for the complete results).

Of the 19 indices studied, the alternating (zigzag) pattern (with local maxima for even values of *n* and local minima for odd values of *n*) observed for the eSM index appears in 16 cases. This has to do with our choice of $\gamma =n\text{mod}2$. Notice that $n=2l\to \gamma =0$, and hence we only have one type of dissimilarity counter, ${C}_{2l\left(l\right)}$. On the contrary, for odd values of *n* we will have $n=2l+1\to \gamma =1$. Hence, in this case there will be two types of dissimilarity counters, ${C}_{2l+1\left(l\right)}$ and ${C}_{2l+1\left(l+1\right)}$. Now notice that when we go from *n* = 2*l* to *n* = 2*l* + 1 the amount of similarity counters remains constant, while the amount of dissimilarity counters increases (from 1 to 2). This implies that, for a given similarity index *s*:

26 $${s}_{n=2l+1}<{s}_{n=2l}$$

Moreover, when we go from *n* = 2*l* + 1 to *n* = 2*l* + 2, the amount of dissimilarity counters decreases (from 2 to 1) and the amount of similarity counters increases (from 2*l* to 2*l* + 2), so:

27 $${s}_{n=2l+1}<{s}_{n=2l+2}$$

The combination of Eqs. (26) and (27) explains the observed alternating pattern:

28 $${s}_{n=2l}>{s}_{n=2l+1}<{s}_{n=2l+2}$$

On the other hand, the extended Sokal-Sneath (2) (eSS(2)) and extended Jaccard (eJa0) indices (in their non-weighted variants) at some point start to monotonically decrease with *n*. This has to do with the more prominent role of the similarity counters in the denominators of these indices. In these cases the increase in the types of similarity counters with increasing *n* actually causes the numerator to grow less rapidly than the denominator (since the counters in the latter are not weighted). Finally, the extended Goodman–Kruskal (eGK) is a singular example, since it clearly presents an alternating pattern, but with local maxima for odd values of *n* and local minima for even values of *n*. This behavior can be explained by the unique way in which the similarity counters are considered in the definition of this index. Before concluding this analysis it is worth noting that, as seen in Fig. 1, the general trends observed in the variation of the average (of the absolute value) of the comparisons for an individual index do not depend strongly on the fingerprint length.

### Analysis of mean similarity indices

**Fig. 2:**
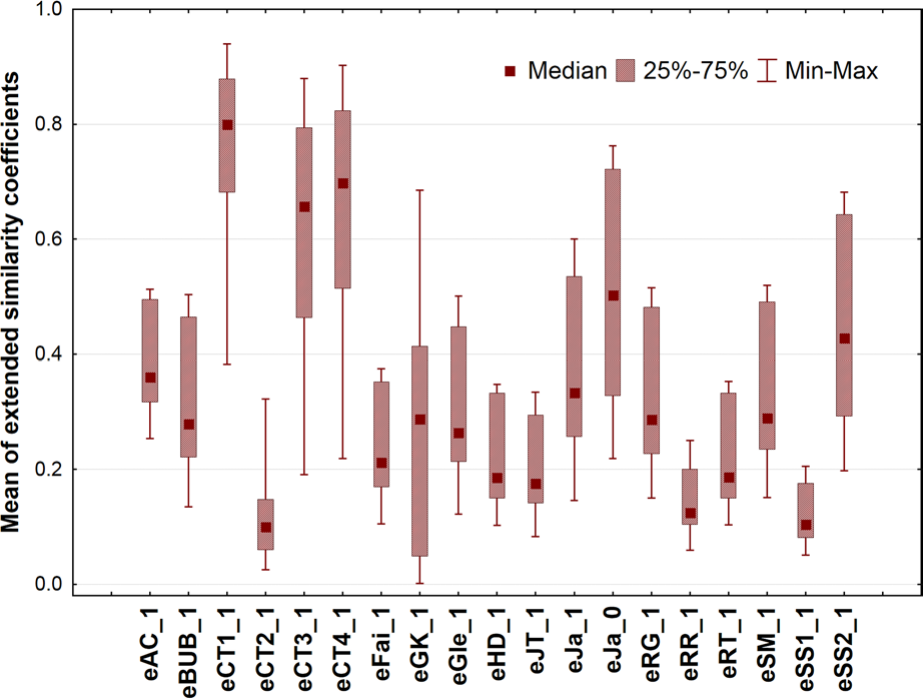
Box and whisker plot of extended similarity coefficents. Maximum number of fingerprints to be compared is 16

A simple box and whisker plot shows the variability of novel indices: median, interquartile range, minimum and maximum are plotted (Fig. 2).

In Fig. 2, the indices occupy different ranges and cover the domain from zero to (almost) 1. No definite trend can be observed. Hence, the idea seems to be plausible: all extended similarity indices express the similarity of molecules with error. Then, variance analysis is a suitable technique to decompose the effects of different factors. The following factors were considered: F2–number (*n*) of objects (fingerprints) compared, 14 levels: *n* = 2, 3, …15; F3–role of weighting, two levels: weighted and non-weighted versions; F4–the extended similarity coefficients themselves: 19 levels; F5–length of the fingerprints, four levels: *m* = 10, 100, 1000, 100 000 (F1 being a dummy factor for the cross-validation iterations). Altogether 14*2*19*4 = 2128 items (averages of similarity indices) have been decomposed into the above factors. As expected, the means of the extended indices also show a characteristic zigzag pattern with homogeneous variance (see Additional file 1: Figures S1–S20), which is consistent with the results shown in Fig. 1.

**Fig. 3:**
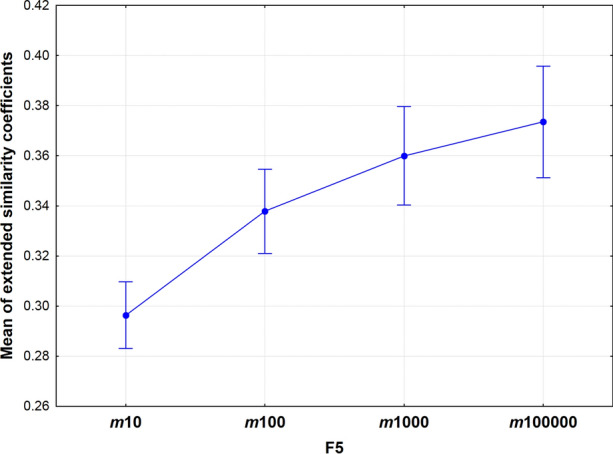
Mean of extended similarity coefficients as a function of fingerprint length. The length of the fingerprint is given as numbers of the x axis after *m*

The effect of fingerprint length on the overall means is plotted in Fig. 3: here, a definite increasing trend can be seen. Moreover, the variances are also increasing with the fingerprint length (heteroscedasticity). It seems that the curve approximates a limit value (saturation) at a relatively small number: ~ 0.38–0.39.

**Fig. 4:**
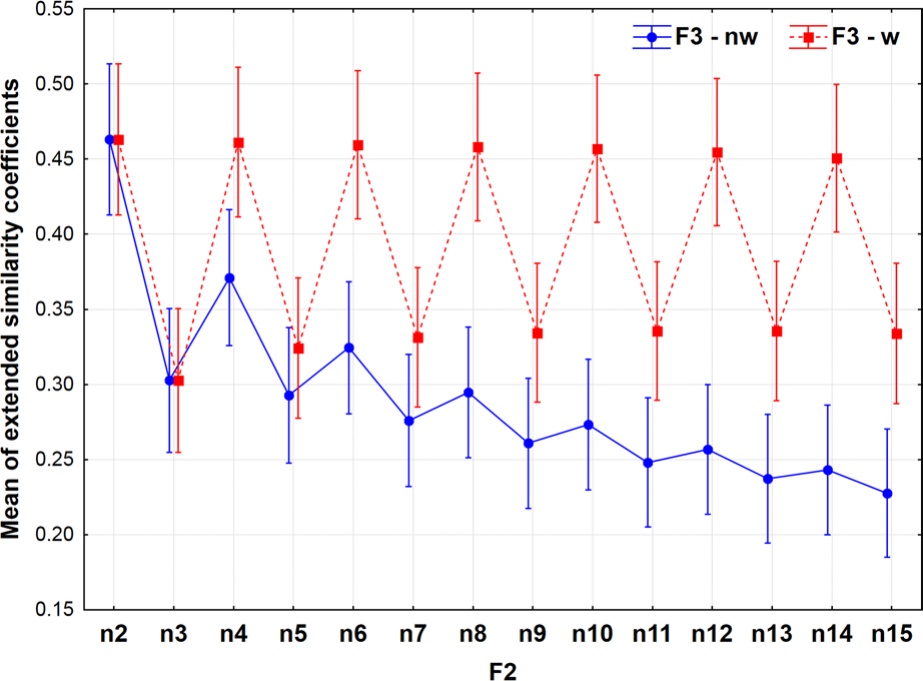
The effect of weighting on the means of extended similarity coefficients as a function of compared objects (fingerprints), w = weighted, nw = non-weighted

The means of extended similarity coefficients can be decomposed into interaction terms, as *e.g*. F2*F3. The role of weighting as a function of the multiplicity of fingerprint comparisons is illustrated in Fig. 4.

The previously observed zigzag pattern can also be seen here, but the patterns split at *n* > 3: means of nonweighted coefficients show a damped zigzag pattern. Although the gap between weighted and non-weighted means increases as the number of compared objects increases, the difference gets smaller between odd and even values of *n*. The variances remain almost constant, as the multiplicity of comparisons increases. The conclusion is obvious, there is no use of weighting for binary and ternary comparisons. The largest difference in terms of weighting is for even numbers and 14-ary comparisons (in the studied range of *n* at least). Notice how the weighted versions of coefficients have higher means than the non-weighted versions. This is an expected result, since while the numerators of both the weighted and non-weighted indices are the same, the denominators of the former are never greater than those of the latter.

**Fig. 5:**
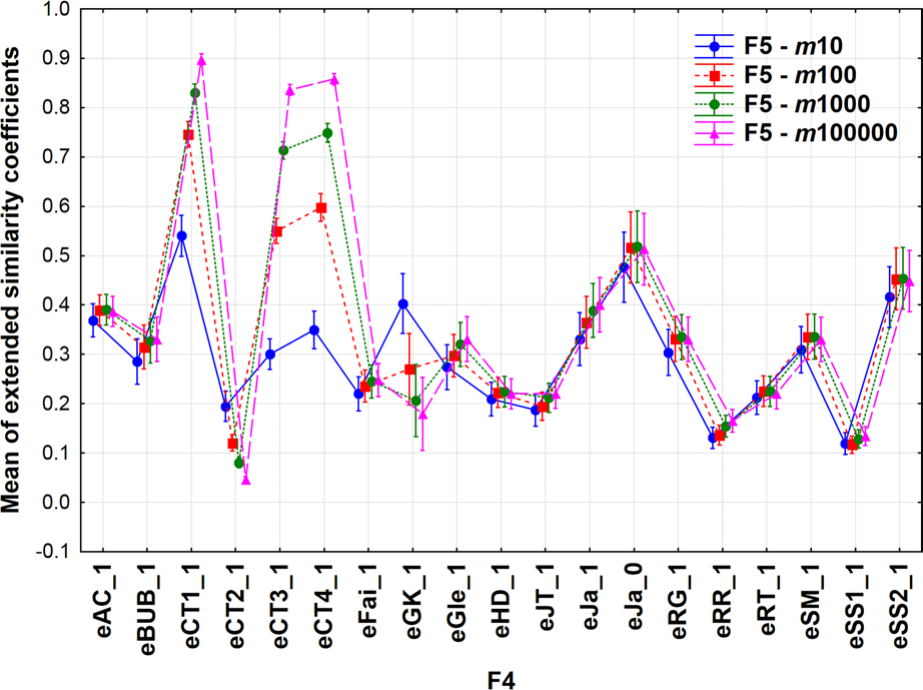
Means of extended similarity coefficients. Line plots correspond to the length of the fingerprints (F5). The abbreviations of coefficients can be found in Appendix 1

There are many more interactions between the factors, but most of them are hard to perceive. However, the coupling between F4*F5 shows a different behavior of the extended similarity coefficients as a function of fingerprint lengths (Fig. 5).

The individual coefficients cover different ranges as in Fig. 1 and their variances are also highly divergent. eCT2_1, eRR_1 and eSS1_1 exhibit small variances, whereas those of eGK_1, eJa_0 and eSS2_1 are higher. Generally, shorter fingerprints show larger variances. In fact, most of the means of extended similarity coefficients are insensitive to the fingerprint lengths. However, three of the extended coefficients defined originally by Consonni and Todeschini (eCT1_1, eCT3_1 and eCT4_1) exhibit highly diverging behavior as the fingerprint lengths increase, with two more coefficients (eCT2_1 and eGK_1) behaving similarly, but to a lesser extent. The latter two produce a reverse ordering (*c.f*. color codes) than the highly diverging indices. These behaviors can be easily understood if we look at these indices’ formulas (see Appendix 1: Table 1). In most of the cases, the numerators and denominators only include terms that are functions of *a*, *b*, *c*, and *d*. As the fingerprint length increases, these terms also increase in a roughly proportional way, so their ratio will remain approximately constant. In the case of the CT indices, however, we have some “1 + ” terms that break this proportionality, which means that the mean value of these indices will indeed depend on the fingerprint length. This explains why in the limit of infinite fingerprint length, eCT1, eCT3, and eCT4 all tend to 1, while eCT2 tends to 0.

The features of the indices have far reaching consequences. In the next chapter, we aim to determine which one should be chosen optimally.

### Analysis of SRD data

As SRD is a preferable multicriteria optimization tool, it can be advantageously applied to select the best and recommendable indices for further usage. In our example, the total number of fingerprints was 16. The SRD input matrix has been changed as the numbers of compared objects changed from *n* = 2 to *n* = 15. The number of rows in the input SRD matrix was given by the binomial coefficients: 16!/[*n*!*(16-*n*)!] where 1 < *n* < 16. The smallest number of rows we considered was 15 for *n* = 2 and *n* = 15, then 120 for *n* = 3 and *n* = 14, and so on, whereas the largest number of rows was 12 870 for *n* = 8. The extended similarity coefficients were enumerated in the columns of the SRD input matrix. No data preprocessing was necessary as all coefficients are scaled between 0 and 1.

**Fig. 6:**
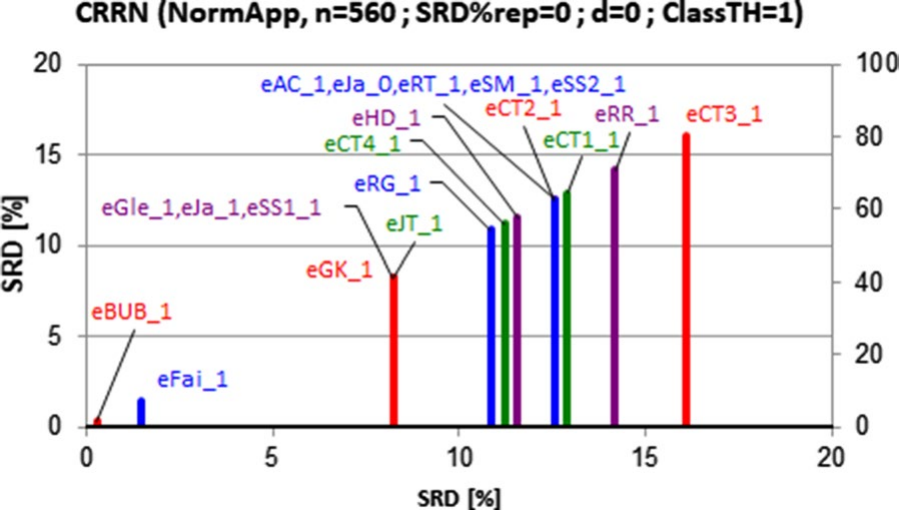
SRD ordering of extended similarity coefficients for a realistic fingerprint length, *m* = 1000, weighting was applied, number of objects compared was *n* = 13. X and left Y axes plot the scaled SRD values between 0 and 100. The Gaussian approximation of the discrete random distribution (~ 60 < SRD <  ~ 70) was omitted for clarity. The abbreviations can be found in Appendix 1

An example SRD result is shown in Fig. 6

The information is represented in such SRD plots as distances from 0 and the random distribution, and the proximity and grouping of the lines. Several extended indices behave similarly (degeneration), coincidence can be seen on some of the lines in case of weighting.

The following factors were considered: F1–variants of sevenfold cross-validation, 2 levels: contiguous and repeated resampling (without and with return, respectively); F2–number (*n*) of compared objects (fingerprints), 14 levels: *n* = 2, 3, … 15; F3–role of weighting, two levels: weighted and non-weighted versions; F4–the similarity coefficients themselves: 19 levels; F5–length of the fingerprints: four levels *m* = 10, 100, 1000, 100 000. Altogether 2*7*14*2*19*4 = 29 792 SRD values were subjected to variance analysis.

**Fig. 7:**
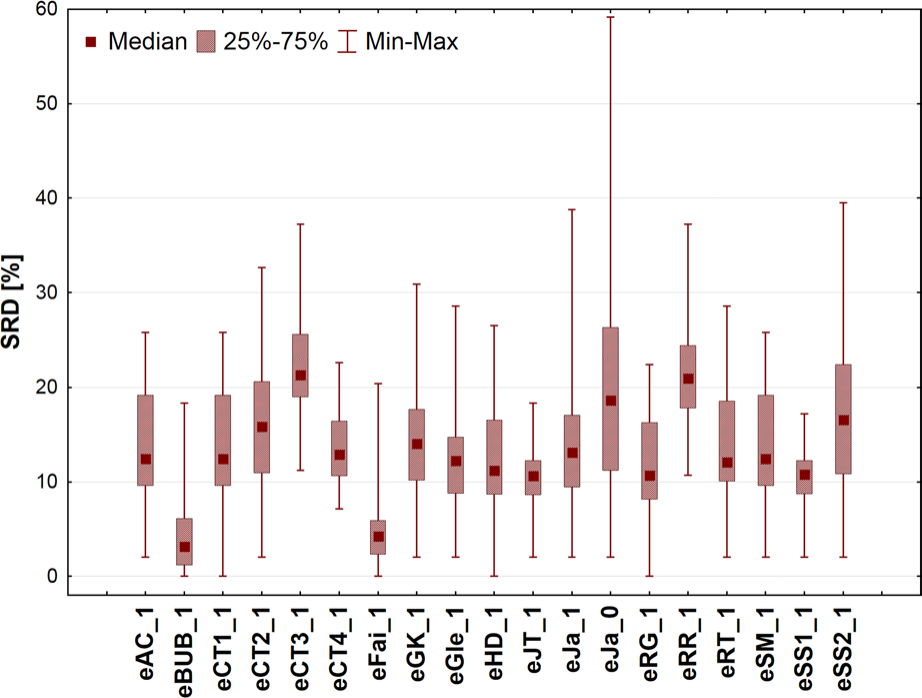
Box and whisker plot for scaled SRD values (between 0 and 100)

Although the ANOVA completed on SRD scores is basically the same as in the case of the mean similarity values, one crucial difference should be mentioned. As SRD is a city block (Manhattan) distance to a gold standard, the smallest SRD value means a better scenario, such a way the best/recommendable indices, number of objects compared, etc. can be revealed and selected. This feature is not applicable on the mean similarity values (previous section). Hence, the box and whisker plot (Fig. 7) shows some rearrangements as compared to that of the similarity values (Fig. 2).

Two indices immediately arise as preferable ones (closest to the reference): eBUB_1 and eFai_1, whereas eJa_0 (which includes the 0-similarity counters) has the largest variability. The nice behavior of the eBUB_1 index is in perfect agreement with the properties of the binary BUB index, as observed in our metabolomics study [17].

**Fig. 8:**
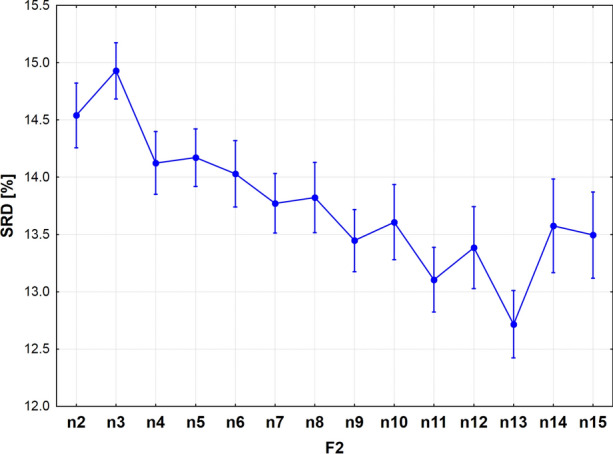
Sum of ranking differences (SRD) scores scaled between 0 and 100 as a function of the number of compared objects (*n*)

The dependence on the multiplicity on comparisons preserves the zigzag pattern, but is somewhat distorted (Fig. 8). The essential difference is that the generally decreasing trend has two jumps at *n* = 3 and *n* = 14, discouraging the usage of these numbers of objects compared. The minimum is reached at *n* = 13, which is therefore suggested as the best number of objects to compare (closest to the reference).

**Fig. 9:**
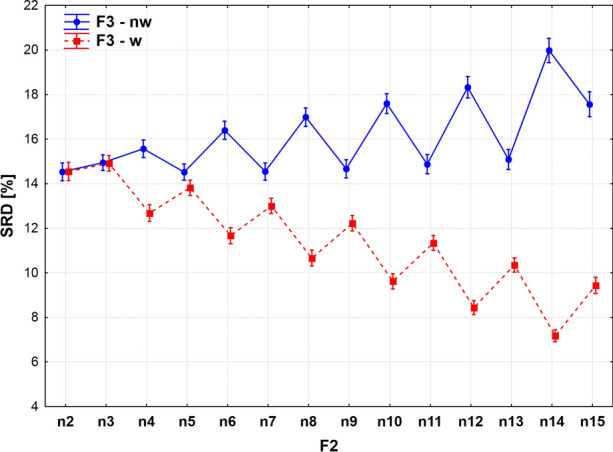
Sum of ranking differences scaled between 0 and 100 as a function of the number of compared objects (*n*) for weighted and non-weighted extended similarity indices

The above statement may be nuanced by the fact that weighting has a different effect on the multiplicity in case of the SRD values (Fig. 9), if *n* > 3.

While weighting has no influence at the beginning for *n* = 2 and 3; non-weighted coefficients show a somewhat increasing-alternating trend deviating from the gold standard. Comparison of 14 objects exhibits the highest gap between the weighted and non-weighted scenarios. In general, weighting is recommended above an *n* value of three.

**Fig. 10:**
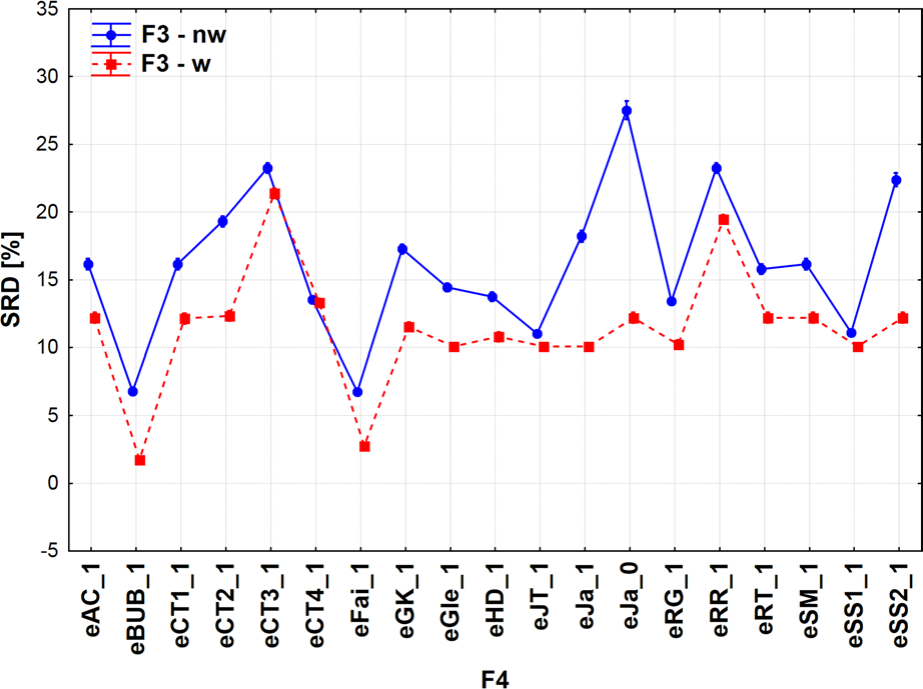
Effect of weighting on the extended similarity coefficients (w = weighted, nw = non-weighted)

Figure 10 shows the effects of weighting on the extended similarity coefficients. Generally, weighting is advantageous (smaller SRD values), and the confidence intervals are so small that they are barely visible (except for eJa_0 non-weighted). It means that all coefficients provided significantly different results, significantly different distances from the reference (consensus). There are some cases when weighting plays a subordinate role: eCT4_1 and eSS1_1. Two indices manifest highly advantageous features (close to the reference): eBUB_1 and eFai_1, with and without weighting, alike. Some indices are relatively good, especially in weighted forms, and they are indistinguishable from each other: eGle_1, eJT_1, eJa_1, eRG_1, and eSS_1. The Jaccard-Tanimoto coefficient in its extended form is also an acceptable choice, though there are some “better” indices (*i.e.* more consistent with the consensus).

**Fig. 11:**
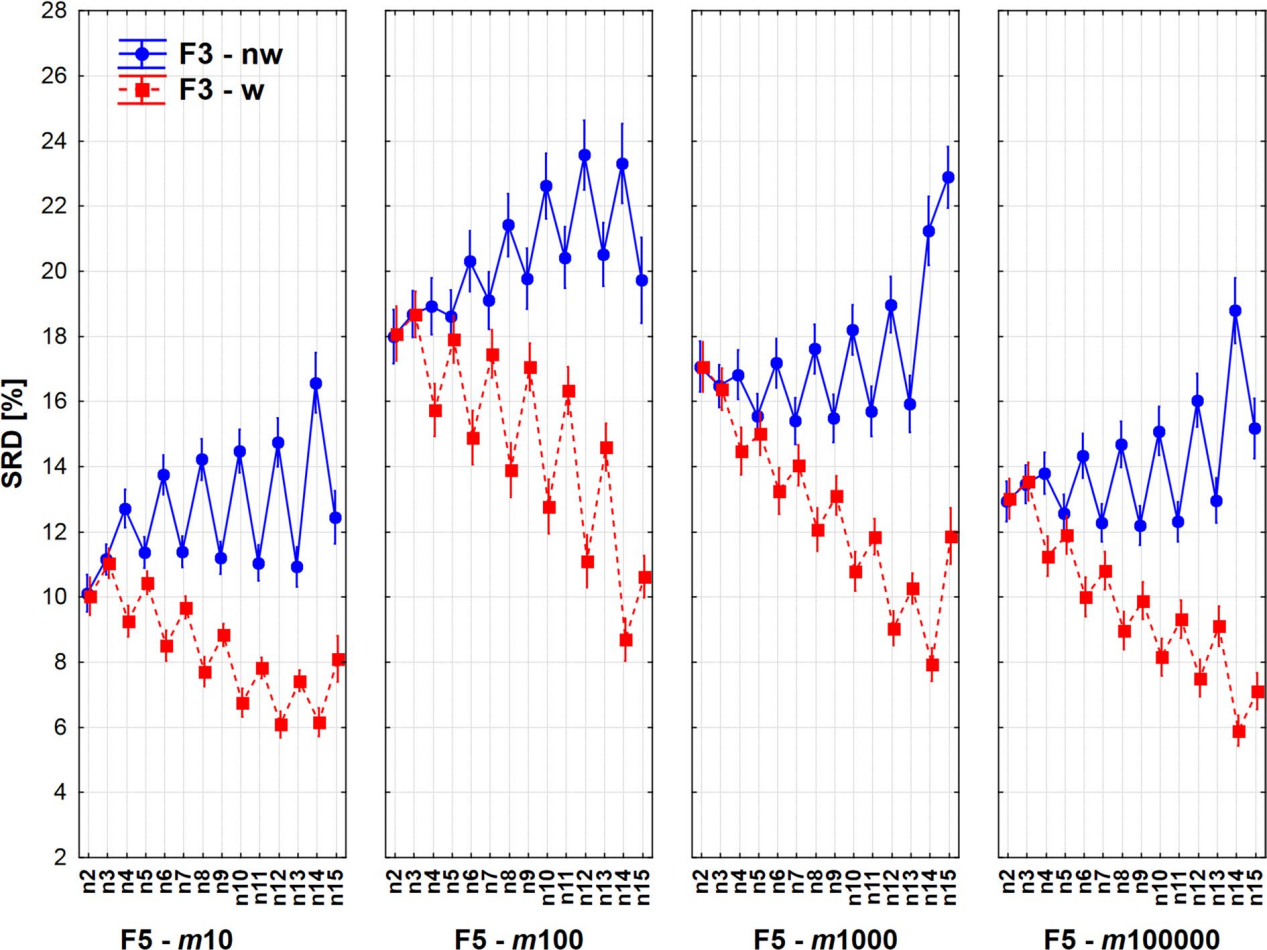
Effect of the number of compared objects on (weighted and non-weighted) extended comparisons of fingerprints with various lengths

Similarly, equivalent indices can be observed from among the non-weighted ones: eAC_1, eCT1_1, eRT_1 and eSM_1. Finally, the interplay of three factors F2*F3*F5 is presented in Fig. 11: number of compared objects, weighting and fingerprint length. It is understandable that the smallest fingerprint length produces the smallest SRD values. The realistic 1000 bit-length fingerprint has an intermediate position, especially *n* = 14 is an outlier, still it is recommendable for further usage if using weighting.

## Summary and conclusion

The present work introduces a series of new similarity indices, which can be applied for the comparison of more than two objects (bitvectors) at once. The essence of the novel extended similarity coefficients is their ability to compare multiple objects at the same time. The features of the average similarity coefficents was studied in detail: the effects of multiplicity, role of weighting, and the fingerprint length have also been studied by variance analysis. A multicriteria decision tool (sum of ranking differences) allowed to select the most advantageous similarity coefficents. We conclude that in general, comparing a larger number of objects (*n* = 12–14) with weighted similarity indices is more advantageous. Two indices are manifested as highly advantageous (close to the reference): eBUB_1 and eFai_1, with and without weighting, alike.

Currently, chemical diversity of a set of molecules is calculated as the mean of all the pairwise similarities between the molecules of the set (order O(*N*^2^)). The framework that we introduced here provides a new alternative, which allows to simultaneously compare more than two dichotomous vectors. This scales in order O(*N*), presenting a tremendous speed gain: this is further discussed in the accompanying paper [22]. Applications include subset selection, clustering, diversity picking or we can even apply this methodology to estimate the diversities of entire compound libraries.

### Supplementary Information

**Additional file 1.** Tables S1–S5 and figures S1–S21 with binary similarity formulas, rankings and ANOVA results.

## Data Availability

Python code for calculating the extended similarity metrics is freely available at: https://github.com/ramirandaq/MultipleComparisons.

## Appendix 1

See Table 1.

## Appendix 2: Extended Sokal-Michener index for quaternary (4-ary) and quintenary (5-ary) comparisons

In order to exemplify the work with the new concepts introduced in the manuscript, and to illustrate the work with the *n*-ary similarity indices, here we present a detailed calculation of the eSM index in two different cases.

*4-ary eSM*:

To fix ideas, let us compare the following four fingerprints:

$$\begin{array}{c}{F}_{1}=\left(1 0 1 1 0 1 0 0\right)\\ \begin{array}{c}{F}_{2}=\left(0 0 1 0 0 1 0 1\right)\\ {F}_{3}=\left(1 0 1 1 1 0 0 1\right)\\ {F}_{4}=\left(0 0 1 1 0 1 0 0\right)\end{array}\end{array}$$

a) The first step is to calculate the ${C}_{4(k)}$ counters, keeping in mind that $0\le k\le 4$:

2.1 $${C}_{4(4)}=1;{C}_{4(3)}=2;{C}_{4(2)}=2;{C}_{4(1)}=1;{C}_{4(0)}=2$$

At this step we can do a quick check and test the following identity: $\sum_{k=0}^{n}{C}_{n(k)}=m$, where *m* is the length of the fingerprints. In this case ${C}_{4(4)}+{C}_{4(3)}+{C}_{4(2)}+{C}_{4(1)}+{C}_{4(0)}=1+2+2+1+2=8$.

b) The second step is calculating the ${\Delta }_{4(k)}=|2k-4|$ values:

2.2 $${\Delta }_{4(4)}=4;{\Delta }_{4(3)}=2;{\Delta }_{4(2)}=0;{\Delta }_{4(1)}=2;{\Delta }_{4(0)}=4$$

c) The third step is to select a coincidence threshold, *γ*. Here we will follow the same convention as in the manuscript, taking $\gamma =n\text{mod}2$:

2.3 $$\begin{array}{c}\gamma =4\text{mod}2\\ \gamma =0\end{array}$$

d) Now we can classify the ${C}_{4(k)}$ in 1-similarity (if $2k-n>\gamma$), 0-similarity (if $n-2k>\gamma$), or dissimilarity (if ${\Delta }_{n(k)}\le \gamma$) counters:

2.4 $$\begin{array}{c}2\times 4-4>0\to {C}_{4\left(4\right)} \, \text{is a } \,1-\text{similarity counter}\\ 2\times 3-4>0\to {C}_{4\left(3\right)} \, \text{is a } \,1-\text{similarity counter}\\ \begin{array}{c}2\times 2-4\le 0\to {C}_{4\left(2\right)} \text{is a dissimilarity counter}\\ 4-2\times 1>0\to {C}_{4\left(1\right)} \, \text{is a } \, 0-\text{similarity counter}\\ 4-2\times 0>0\to {C}_{4\left(0\right)} \, \text{is a } \, 0-\text{similarity counter}\end{array}\end{array}$$

e) The next step is to select the weight functions, ${f}_{s}\left({\Delta }_{n(k)}\right)$ and ${f}_{d}\left({\Delta }_{n(k)}\right)$, and calculate the weigths associated to each ${C}_{4(k)}$. We will make the same convention as in the text, namely: ${f}_{s}\left({\Delta }_{n(k)}\right)=\frac{{\Delta }_{n(k)}}{n}$, and ${f}_{d}\left({\Delta }_{n(k)}\right)=1-\frac{{\Delta }_{n(k)}-n\text{mod}2}{n}$. In this particular case these translate to:

2.5 $${f}_{s}\left({\Delta }_{4(k)}\right)=\frac{{\Delta }_{4(k)}}{4}$$

2.6 $${f}_{d}\left({\Delta }_{4(k)}\right)=1-\frac{{\Delta }_{4(k)}}{4}$$

For the similarity counters:

2.7 $$\begin{array}{c}{f}_{s}\left({\Delta }_{4(4)}\right)=\frac{4}{4}=1\\ {f}_{s}\left({\Delta }_{4(3)}\right)=\frac{2}{4}=\frac{1}{2}\\ \begin{array}{c}{f}_{s}\left({\Delta }_{4(1)}\right)=\frac{2}{4}=\frac{1}{2}\\ {f}_{s}\left({\Delta }_{4(0)}\right)=\frac{4}{4}=1\end{array}\end{array}$$

For the dissimilarity counter:

2.8 $${f}_{d}\left({\Delta }_{4(2)}\right)=1-\frac{0}{4}=1$$

f) Finally, we just need to combine all of these magnitudes according to the definitions of the extended index that we want to calculate. To do this we follow the expressions listed in Appendix 1. For the two ${s}_{eSM}$ indices we have:

f1) $${s}_{eSM\left(1s\_wd\right)}$$:

2.9 $${s}_{eSM\left(1s\_wd\right)}=\frac{\sum_{s}{f}_{s}\left({\Delta }_{n(k)}\right){C}_{n(k)}}{\sum_{s}{f}_{s}\left({\Delta }_{n(k)}\right){C}_{n(k)}+\sum_{d}{f}_{d}\left({\Delta }_{n(k)}\right){C}_{n(k)}}$$

2.10 $${s}_{eSM\left(1s\_wd\right)}=\frac{{f}_{s}\left({\Delta }_{4(4)}\right){C}_{4(4)}+{f}_{s}\left({\Delta }_{4(3)}\right){C}_{4(3)}+{f}_{s}\left({\Delta }_{4(1)}\right){C}_{4(1)}+{f}_{s}\left({\Delta }_{4(0)}\right){C}_{4(0)}}{{f}_{s}\left({\Delta }_{4(4)}\right){C}_{4(4)}+{f}_{s}\left({\Delta }_{4(3)}\right){C}_{4(3)}+{f}_{s}\left({\Delta }_{4(1)}\right){C}_{4(1)}+{f}_{s}\left({\Delta }_{4(0)}\right){C}_{4(0)}+{f}_{d}\left({\Delta }_{4(2)}\right){C}_{4(2)}}$$

2.11 $${s}_{eSM\left(1s\_wd\right)}=\frac{1\times 1+0.5\times 2+0.5\times 1+1\times 2}{1\times 1+0.5\times 2+0.5\times 1+1\times 2+1\times 2}=\frac{4.5}{6.5}= 0.6923$$

f2) $${s}_{eSM\left(1s\_d\right)}$$:

2.12 $${s}_{eSM\left(1s\_d\right)}=\frac{\sum_{s}{f}_{s}\left({\Delta }_{n(k)}\right){C}_{n(k)}}{\sum_{s}{C}_{n(k)}+\sum_{d}{C}_{n(k)}}$$

2.13 $${s}_{eSM\left(1s\_d\right)}=\frac{{f}_{s}\left({\Delta }_{4(4)}\right){C}_{4(4)}+{f}_{s}\left({\Delta }_{4(3)}\right){C}_{4(3)}+{f}_{s}\left({\Delta }_{4(1)}\right){C}_{4(1)}+{f}_{s}\left({\Delta }_{4(0)}\right){C}_{4(0)}}{{C}_{4(4)}+{C}_{4(3)}+{C}_{4(1)}+{C}_{4(0)}+{C}_{4(2)}}$$

2.14 $${s}_{eSM\left(1s\_d\right)}=\frac{1\times 1+0.5\times 2+0.5\times 1+1\times 2}{1+2+1+2+2}=\frac{4.5}{8.0}=0.5625$$

*5-ary eSM*:

The comparison of an odd number of molecules is largely equivalent to the comparison of an even number of object. Let us illustrate this with the comparison of the following five fingerprints:

$$\begin{array}{c}{F}_{1}=\left(1 0 1 1 0 1 0 0\right)\\ \begin{array}{c}{F}_{2}=\left(0 0 1 0 0 1 0 1\right)\\ {F}_{3}=\left(1 0 1 1 1 0 0 1\right)\\ \begin{array}{c}{F}_{4}=\left(0 0 1 1 0 1 0 0\right)\\ {F}_{5}=\left(0 0 1 1 0 1 1 0\right)\end{array}\end{array}\end{array}$$

a) Calculating the ${C}_{5(k)}$ counters, keeping in mind that $0\le k\le 5$:

2.15 $${C}_{5(5)}=1;{C}_{5(4)}=2;{C}_{5(3)}=0;{C}_{5(2)}=2;{C}_{5(1)}=2;{C}_{5(0)}=1$$

Now $${C}_{5(5)}+{C}_{5(4)}+{C}_{5(3)}+{C}_{5(2)}+{C}_{5(1)}+{C}_{5(0)}=1+2+0+2+2+1=8$$.

b) Calculating the ${\Delta }_{5(k)}=|2k-5|$ values:

2.16 $${\Delta }_{5(5)}=5;{\Delta }_{5(4)}=3;{\Delta }_{5(3)}=1;{\Delta }_{5(2)}=1;{\Delta }_{5(1)}=3;{\Delta }_{5(0)}=5$$

c) Selecting a coincidence threshold, *γ*. Here we will also take $\gamma =n\text{mod}2$:

2.17 $$\begin{array}{c}\gamma =5\text{mod}2\\ \gamma =1\end{array}$$

d) Classifying the ${C}_{5(k)}$ in 1-similarity (if $2k-n>\gamma$), 0-similarity (if $n-2k>\gamma$), or dissimlarity (if ${\Delta }_{n(k)}\le \gamma$) counters:

2.18 $$\begin{array}{c}\begin{array}{c}2\times 5-5>1\to {C}_{5\left(4\right)} \, \text{is a } \, 1-\text{similarity counter}\\ 2\times 4-5>1\to {C}_{5\left(4\right)} \, \text{is a } \, 1-\text{similarity counter}\end{array}\\ 2\times 3-5\le 1\to {C}_{5\left(3\right)} \text{is a dissimilarity counter}\\ \begin{array}{c}2\times 2-5\le 1\to {C}_{5\left(2\right)} \text{is a dissimilarity counter}\\ 5-2\times 1>1\to {C}_{5\left(1\right)} \, \text{is a } \, 0-\text{similarity counter}\\ 5-2\times 0>1\to {C}_{5\left(0\right)} \, \text{is a } \, 0-\text{similarity counter}\end{array}\end{array}$$

This shows the biggest difference between the comparison of even or odd numbers of molecules, namely, the presence of more dissimilarity counters when we compare and odd number of molecules.

e) Selecting the weight functions, ${f}_{s}\left({\Delta }_{n(k)}\right)$ and ${f}_{d}\left({\Delta }_{n(k)}\right)$, and calculating the weigths associated to each ${C}_{5(k)}$. We will make the same convention, namely: ${f}_{s}\left({\Delta }_{n(k)}\right)=\frac{{\Delta }_{n(k)}}{n}$, and ${f}_{d}\left({\Delta }_{n(k)}\right)=1-\frac{{\Delta }_{n(k)}-n\text{mod}2}{n}$. In this particular case these translate to:

2.19 $${f}_{s}\left({\Delta }_{5(k)}\right)=\frac{{\Delta }_{5(k)}}{5}$$

2.20 $${f}_{d}\left({\Delta }_{5(k)}\right)=1-\frac{{\Delta }_{5(k)}-1}{5}$$

For the similarity counters:

2.21 $$\begin{array}{c}{f}_{s}\left({\Delta }_{5(5)}\right)=\frac{5}{5}=1\\ {f}_{s}\left({\Delta }_{5(4)}\right)=\frac{3}{5}\\ \begin{array}{c}{f}_{s}\left({\Delta }_{5(1)}\right)=\frac{3}{5}\\ {f}_{s}\left({\Delta }_{5(0)}\right)=\frac{5}{5}=1\end{array}\end{array}$$

For the dissimilarity counters:

2.22 $$\begin{array}{c}{f}_{d}\left({\Delta }_{5(3)}\right)=1-\frac{1-1}{5}=1\\ {f}_{d}\left({\Delta }_{5(2)}\right)=1-\frac{1-1}{5}=1\end{array}$$

f) Finally, we combine all of these magnitudes according to the definitions of the extended index that we want to calculate. For the two ${s}_{eSM}$ indices we have:

f1) $${s}_{eSM\left(1s\_wd\right)}$$:

2.23 $${s}_{eSM\left(1s\_wd\right)}=\frac{\sum_{s}{f}_{s}\left({\Delta }_{n(k)}\right){C}_{n(k)}}{\sum_{s}{f}_{s}\left({\Delta }_{n(k)}\right){C}_{n(k)}+\sum_{d}{f}_{d}\left({\Delta }_{n(k)}\right){C}_{n(k)}}$$

2.24 $${s}_{eSM\left(1s\_wd\right)}=\frac{{f}_{s}\left({\Delta }_{5(5)}\right){C}_{5(5)}+{f}_{s}\left({\Delta }_{5(4)}\right){C}_{5(4)}+{f}_{s}\left({\Delta }_{5(1)}\right){C}_{5(1)}+{f}_{s}\left({\Delta }_{5(0)}\right){C}_{5(0)}}{{f}_{s}\left({\Delta }_{5(5)}\right){C}_{5(5)}+{f}_{s}\left({\Delta }_{5(4)}\right){C}_{5(4)}+{f}_{s}\left({\Delta }_{5(1)}\right){C}_{5(1)}+{f}_{s}\left({\Delta }_{5(0)}\right){C}_{5(0)}+{f}_{d}\left({\Delta }_{5(3)}\right){C}_{5(3)}+{f}_{d}\left({\Delta }_{5(2)}\right){C}_{5(2)}}$$

2.25 $${s}_{eSM\left(1s\_wd\right)}=\frac{1\times 1+0.6\times 2+0.6\times 2+1\times 1}{1\times 1+0.6\times 2+0.6\times 2+1\times 1+1\times 0+1\times 2}=\frac{4.4}{6.4}=0.6875$$

f2) $${s}_{eSM\left(1s\_d\right)}$$:

2.26 $${s}_{eSM\left(1s\_d\right)}=\frac{\sum_{s}{f}_{s}\left({\Delta }_{n(k)}\right){C}_{n(k)}}{\sum_{s}{C}_{n(k)}+\sum_{d}{C}_{n(k)}}$$

2.27 $${s}_{eSM\left(1s\_d\right)}=\frac{{f}_{s}\left({\Delta }_{5(5)}\right){C}_{5(5)}+{f}_{s}\left({\Delta }_{5(4)}\right){C}_{5(4)}+{f}_{s}\left({\Delta }_{5(1)}\right){C}_{5(1)}+{f}_{s}\left({\Delta }_{5(0)}\right){C}_{5(0)}}{{C}_{5(5)}+{C}_{5(4)}+{C}_{5(1)}+{C}_{5(0)}+{C}_{5(3)}+{C}_{5(2)}}$$

2.28 $${s}_{eSM\left(1s\_d\right)}=\frac{1\times 1+0.6\times 2+0.6\times 2+1\times 1}{1+2+2+1+0+2}=\frac{4.4}{8.0}=0.5500$$

## Appendix 3: Extended dissimilarity indices

The concepts presented in the manuscript can be straightforwardly used to define *n*-ary dissimilarity indices. As a simple example we will consider the well-known Hamming distance, ${d}_{Ham}$, that measures the number of bits that are different between two given fingerprints. In the standard binary case (and using the notation introduced at the beginning of this paper), this can be easily written as:

3.1 $${d}_{Ham}=b+c$$

Now it is easy to see that the extended *n*-ary flavor of this index is nothing more than the sum of all the dissimilarity counters, namely:

3.2 $${d}_{eHam}=\sum_{d}{f}_{d}\left({\Delta }_{n(k)}\right){C}_{n(k)}$$

Finally, it is worth noting that we can define a normalized version of the Hamming distance, ${d}_{eHam\_n}$, by dividing the standard expression for this index by the length of the fingerprints:

3.3 $${d}_{eHam\_n}=\frac{b+c}{p}$$

This form is closely related to the Sokal-Michener index, since:

3.4 $${d}_{Ham\_n}=\frac{b+c}{p}=1-\frac{a+d}{p}=1-{s}_{SM}$$

Then, we can trivially use the extended expressions of the Sokal-Michener index in order to obtain the *n*-ary forms of the normalized Hamming distance.

3.5 $${d}_{eHam\_n\left(1s\_wd\right)}=1-\frac{\sum_{s}{f}_{s}\left({\Delta }_{n(k)}\right){C}_{n(k)}}{\sum_{s}{f}_{s}\left({\Delta }_{n(k)}\right){C}_{n(k)}+\sum_{d}{f}_{d}\left({\Delta }_{n(k)}\right){C}_{n(k)}}$$

3.6 $${d}_{eHam\_n\left(1s\_d\right)}=1-\frac{\sum_{s}{f}_{s}\left({\Delta }_{n\left(k\right)}\right){C}_{n\left(k\right)}}{\sum_{s}{C}_{n\left(k\right)}+\sum_{d}{C}_{n\left(k\right)}} $$

This simple procedure can be extended to other dissimilarity indices.

## References

[1] Todeschini R, Consonni V, Xiang H, Holliday J, Buscema M, Willett P (2012). Similarity coefficients for binary chemoinformatics data: overview and extended comparison using simulated and real data sets. J Chem Inf Model.

[2] Rácz A, Bajusz D, Héberger K (2018). Life beyond the Tanimoto coefficient: similarity measures for interaction fingerprints Journal of. Cheminformatics.

[3] Eckert H, Bajorath J (2007). Molecular similarity analysis in virtual screening: foundations, limitations and novel approaches. Drug Discov Today.

[4] Keserü GM, Makara GM (2009). The influence of lead discovery strategies on the properties of drug candidates. Nat Rev Drug Discov.

[5] Cherkasov A, Muratov E, Fourches D, Varnek A, Baskin I, Cronin M, Dearden J, Gramatica P, Martin YC, Todeschini R, Consonni V, Kuz'min VE, Cramer R, Benigni R, Yang C, Rathman J, Terfloth L, Gasteiger J, Richard A, Tropsha A (2014). QSAR modeling: where have you been? Where are you going to?. J Med Chem.

[6] Stumpfe D, Bajorath J (2012). Exploring activity cliffs in medicinal chemistry. J Med Chem.

[7] Cortes-Ciriano I, Firth NC, Bender A, Watson O (2018). Discovering highly potent molecules from an initial set of inactives using iterative screening. J Chem Inf Model.

[8] Bender A, Glen RC (2004). Molecular similarity: a key technique in molecular informatics. Org Biomol Chem.

[9] Heidar Zadeh F, Ayers PW (2013). Molecular alignment as a penalized permutation Procrustes problem. J Math Chem.

[10] Alcoba DR, Lain L, Torre A, Ona OB, Tiznado W (2012). Ground and excited state similarity studies by means of Fukui and dual-descriptor matrices Chem. Phys Lett.

[11] Ayers PW, Carbo-Dorca R (2011). The relationship between the eigenvalues and eigenvectors of a similarity matrix and its associated Carbo index matrix. J Math Chem.

[12] Miranda-Quintana RA, Cruz-Rodes R, Codorniu-Hernandez E, Batista-Leyva AJ (2010). Formal theory of the comparative relations: its application to the study of quantum similarity and dissimilarity measures and indices. J Math Chem.

[13] Borgoo A, Torrent-Sucarrat M, De Proft F, Geerlings P (2007). Quantum similarity study of atoms: a bridge between hardness and similarity indices. J Chem Phys.

[14] Carbo-Dorca R, Leyda L, Arnau M (1980). How similar is a molecule to another? An electron density measure of similarity between two molecular structures Int. J Quantum Chem.

[15] Willett P (2006). Similarity-based virtual screening using 2D fingerprints. Drug Discov Today.

[16] Todeschini R, Ballabio D, Consonni V (2015). Encyclopedia of analytical chemistry: applications, theory and instrumentation.

[17] Rácz A, Bajusz D, Héberger K (2018). Binary similarity measures for fingerprint analysis of qualitative metabolomic profiles. Metabolomics.

[18] Bajusz D, Rácz A, Héberger K (2017) Comprehensive medicinal chemistry III. In: Chackalamannil S, Rotella D, Ward SE (Eds). Elsevier, Amsterdam

[19] Bajusz D, Rácz A, Héberger K (2015). Why is Tanimoto index an appropriate choice for fingerprint-based similarity calculations?. J Cheminformatics.

[20] Miranda-Quintana RA, Kim TD, Heidar-Zadeh F, Ayers PW (2019). On the impossibility of unambiguously selecting the best model for fitting data. J Math Chem.

[21] Brereton AE, MacKinnon S, Safikhani Z, Reeves S, Alwash S, Shahani V, Windemuth A (2020). Predicting drug properties with parameter-free machine learning: pareto-optimal embedded modeling (POEM). Mach Learn Sci Technol.

[22] Miranda-Quintana RA, Rácz A, Bajusz D, Héberger K (2021). Extended similarity indices: the beneits of comparing more than two objects simultaneously. Part 2: speed, consistency, diversity selection. J Cheminform.

[23] Héberger K (2010). Sum of ranking differences compares methods or models fairly. Trends Anal Chem.

[24] Kollár-Hunek K, Héberger K (2013). Method and model comparison by sum of ranking differences in cases of repeated observations (ties). Chemometr Intell Lab Syst.

[25] Héberger K, Kollár-Hunek K (2011). Sum of ranking differences for method discrimination and its validation: comparison of ranks with random numbers. J Chemom.

[26] Héberger K, Kolarević S, Kračun-Kolarević M, Sunjog K, Gačić Z, Kljajić Z, Mitrić M, Vuković-Gačić B (2014). Evaluation of single cell gel electrophoresis data: combination of variance analysis with sum of ranking differences. Mutation Res Genet Toxicol Environ Mutagenesis.

[27] Héberger K, Kollár-Hunek K (2019). Comparison of validation variants by sum of ranking differences and ANOVA. J Chemom.

[28] Rácz A, Bajusz D, Héberger K (2015). Consistency of QSAR models: Correct split of training and test sets, ranking of models and performance parameters. SAR QSAR Environ Res.

[29] Lourenço J, Lebensztajn L (2018). Post-pareto optimality analysis with sum of ranking differences. IEEE Trans Magn.

[30] Willett P (2013). Combination of similarity rankings using data fusion. J Chem Inf Model.

[31] Andrić F, Bajusz D, Rácz A, Šegan S, Héberger K (2016). Multivariate assessment of lipophilicity scales—computational and reversed phase thin-layer chromatographic indices. J Pharm Biomed Anal.

[32] Stokes TD, Fotein M, Brownfield B, Kalivas JH, Mousdis G, Amine A, Georgiou C (2018). Feasibility assessment of synchronous fluorescence spectral fusion by application to argan oil for adulteration analysis Appl. Spectrosc.

[33] Sipos L, Gere A, Popp J, Kovács S (2018). A novel ranking distance measure combining Cayley and Spearman footrule metrics. J Chemom.

[34] Lindman HR (1991). Analysis of variance in experimental design.

