# Supplementary material for: Extended similarity indices: the benefits of comparing more than two objects simultaneously. Part 1: Theory and characteristics†
Source: J Cheminform. 2021 Apr 23;13:32. doi: 10.1186/s13321-021-00505-3 (PMC8067658; doi:10.1186/s13321-021-00505-3)
Supplement: Supplementary file 1 — Additional file 1. Tables S1–S5 and figures S1–S21 with binary similarity formulas, rankings and ANOVA results. [file 13321_2021_505_MOESM1_ESM.docx]

**Extended similarity indices: the benefits of comparing more than two objects simultaneously. Part 1: Theory and characteristics**

Ramón Alain Miranda-Quintana^1^, Dávid Bajusz^2^, Anita Rácz^3^, Károly Héberger^3^

*^1^ Department of Chemistry, University of Florida, Gainesville, FL 32603, USA*

*^2^ Medicinal Chemistry Research Group, ELKH Research Centre for Natural Sciences, Magyar tudósok krt. 2, 1117, Budapest, Hungary*

*^3^ Plasma Chemistry Research Group, ELKH Research Centre for Natural Sciences, Magyar tudósok krt. 2, 1117, Budapest, Hungary*

**Table S1:** Binary similarity indices

| **General Indices** | | |
| --- | --- | --- |
| **Label** | **Name** | **Equation** |
| For | Forbes | $s_{For}=\frac{pa}{\left( a+b \right)\left( a+c \right)}$ |
| Sim | Simpson | $s_{Sim}=\frac{a}{\min\left\{ \left( a+b \right),\left( a+c \right) \right\}}$ |
| BB | Braun-Blanquet | $s_{BB}=\frac{a}{\max\left\{ \left( a+b \right),\left( a+c \right) \right\}}$ |
| DK | Driver-Kroeber, Ochiai, cosine | $s_{DK}=\frac{a}{\sqrt{\left( a+b \right)\left( a+c \right)}}$ |
| Kul | Kulczynski | $s_{Kul}=\frac{1}{2}\left( \frac{a}{a+b}+\frac{a}{a+c} \right)$ |
| Mou | Mountford | $s_{Mou}=\frac{2a}{ab+ac+2bc}$ |
| Mic | Michael | $s_{Mic}=\frac{4\left( ad-bc \right)}{\left( a+d \right)^{2}+\left( b+c \right)^{2}}$ |
| Yu1 | Yule (1) | $s_{Yu1}=\frac{ad-bc}{ad+bc}$ |
| Yu2 | Yule (2) | $s_{Yu2}=\frac{\sqrt{ad}-\sqrt{bc}}{\sqrt{ad}+\sqrt{bc}}$ |
| Fos | Fossum | $s_{Fos}=\frac{p\left( a-0.5 \right)^{2}}{\left( a+b \right)\left( a+c \right)}$ |
| Den | Dennis | $s_{Den}=\frac{ad-bc}{\sqrt{p\left( a+b \right)\left( a+c \right)}}$ |
| Co1 | Cole (1) | $s_{Co1}=\frac{ad-bc}{\left( a+c \right)\left( c+d \right)}$ |
| Co2 | Cole (2) | $s_{Co2}=\frac{ad-bc}{\left( a+b \right)\left( b+d \right)}$ |
| Dis | Dispersion | $s_{dis}=\frac{ad-bc}{p^{2}}$ |
| SS3 | Sokal-Sneath (3) | $s_{SS3}=\frac{1}{4}\left( \frac{a}{a+b}+\frac{a}{a+c}+\frac{d}{d+b}+\frac{d}{d+c} \right)$ |
| SS4 | Sokal-Sneath (4) | $s_{SS4}=\frac{ad}{\sqrt{\left( a+b \right)\left( a+c \right)\left( d+b \right)\left( d+c \right)}}$ |
| Phi | Pearson-Heron colligation coefficient | $s_{SS4}=\frac{ad-bc}{\sqrt{\left( a+b \right)\left( a+c \right)\left( d+b \right)\left( d+c \right)}}$ |
| Di1 | Dice (1) | $s_{Di1}=\frac{a}{a+b}$ |
| Di2 | Dice (2) | $s_{Di2}=\frac{a}{a+c}$ |
| Sor | Sorgenfrei | $s_{Sor}=\frac{a^{2}}{\left( a+b \right)\left( a+c \right)}$ |
| Coh | Cohen | $s_{Coh}=\frac{2\left( ad-bc \right)}{\left( a+b \right)\left( b+d \right)+\left( a+c \right)\left( c+d \right)}$ |
| Pe1 | Peirce (1) | $s_{Pe1}=\frac{ad-bc}{\left( a+b \right)\left( c+d \right)}$ |
| Pe2 | Peirce (2) | $s_{Pe2}=\frac{ad-bc}{\left( a+c \right)\left( b+d \right)}$ |
| MP | Maxwell-Pilliner | $s_{MP}=\frac{2\left( ad-bc \right)}{\left( a+b \right)\left( c+d \right)+\left( a+c \right)\left( b+d \right)}$ |
| CT5 | Consoni-Todeschini (5) | $s_{CT5}=\frac{\ln\left( 1+ad \right)-\ln\left( 1+bc \right)}{\ln\left( 1+\frac{p^{2}}{4} \right)}$ |
| **Additive indices** | | |
| **Label** | **Name** | **Equation** |
| AC | Austin-Colwell | $s_{AC}=\frac{2}{\pi}\arcsin\sqrt{\frac{a+d}{p}}$ |
| BUB | Baroni-Urbani-Buser | $s_{BUB}=\frac{\sqrt{ad}+a}{\sqrt{ad}+a+b+c}$ |
| CT1 | Consoni-Todeschini (1) | $s_{CT1}=\frac{\ln\left( 1+a+d \right)}{\ln\left( 1+p \right)}$ |
| CT2 | Consoni-Todeschini (2) | $s_{CT2}=\frac{\ln\left( 1+p \right)-\ln\left( 1+b+c \right)}{\ln\left( 1+p \right)}$ |
| Fai | Faith | $s_{Fai}=\frac{a+0.5d}{p}$ |
| GK | Goodman-Kruskal | $s_{GK}=\frac{2\min\left( a,d \right)-b-c}{2\min\left( a,d \right)+b+c}$ |
| HD | Hawkins-Dotson | $s_{HD}=\frac{1}{2}\left( \frac{a}{a+b+c}+\frac{d}{b+c+d} \right)$ |
| RT | Rogers-Tanimoto | $s_{RT}=\frac{a+d}{p+b+c}$ |
| RG | Rogot-Goldberg | $s_{RG}=\frac{a}{2a+b+c}+\frac{d}{b+c+2d}$ |
| SM | Simple matching,  Sokal-Michner | $s_{SM}=\frac{a+d}{p}$ |
| SS2 | Sokal-Sneath (2) | $s_{SS2}=\frac{2a+2d}{p+a+d}$ |
| **Asymmetric indices** | | |
| **Label** | **Name** | **Equation** |
| CT3 | Consoni-Todeschini (3) | $s_{CT3}=\frac{\ln\left( 1+a \right)}{\ln\left( 1+p \right)}$ |
| CT4 | Consoni-Todeschini (4) | $s_{CT4}=\frac{\ln\left( 1+a \right)}{\ln\left( 1+a+b+c \right)}$ |
| Gle | Gleason | $s_{Gle}=\frac{2a}{2a+b+c}$ |
| Ja | Jaccard | $s_{Ja}=\frac{3a}{3a+b+c}$ |
| RR | Russel-Rao | $s_{RR}=\frac{a}{p}$ |
| SS1 | Sokal-Sneath (1) | $s_{SS1}=\frac{a}{a+2b+2c}$ |
| JT | Jaccard-Tanimoto | $s_{JT}=\frac{a}{a+b+c}$ |

**Table S2:** Rankings obtained after comparing 15 fingerprints using non-weighted extended similarity indices. Fingerprints with length m = 10.

| **Ref** | **eAC** | **eBUB** | **eCT1** | **eCT2** | **eCT3** | **eCT4** | **eFai** | **eGK** | **eGle** | **eHD** | **eJT** | **eJa0** | **eJa** | **eRG** | **eRR** | **eRT** | **eSM** | **eSS1** | **eSS2** |
| --- | --- | --- | --- | --- | --- | --- | --- | --- | --- | --- | --- | --- | --- | --- | --- | --- | --- | --- | --- |
| 2.0 | 2.0 | 2.0 | 2.0 | 3.0 | 2.5 | 2.5 | 2.0 | 3.5 | 2.5 | 2.0 | 2.5 | 1.0 | 2.5 | 2.0 | 2.5 | 3.0 | 2.0 | 2.5 | 1.0 |
| 12.0 | 12.5 | 11.0 | 12.5 | 12.5 | 10.5 | 10.5 | 11.0 | 11.5 | 10.5 | 12.5 | 10.5 | 8.5 | 6.5 | 12.5 | 10.5 | 12.5 | 12.5 | 10.5 | 9.5 |
| 6.5 | 5.0 | 6.5 | 5.0 | 5.0 | 7.0 | 7.5 | 6.5 | 6.5 | 7.5 | 5.5 | 7.5 | 6.0 | 9.5 | 5.5 | 7.0 | 5.0 | 5.0 | 7.5 | 6.0 |
| 13.0 | 12.5 | 13.0 | 12.5 | 12.5 | 13.0 | 13.5 | 13.0 | 13.5 | 13.5 | 12.5 | 13.5 | 8.5 | 13.5 | 12.5 | 13.0 | 12.5 | 12.5 | 13.5 | 9.5 |
| 8.5 | 10.0 | 9.5 | 10.0 | 8.0 | 7.0 | 7.5 | 9.0 | 6.5 | 7.5 | 8.5 | 7.5 | 12.0 | 9.5 | 9.5 | 7.0 | 8.0 | 10.0 | 7.5 | 12.0 |
| 8.5 | 10.0 | 9.5 | 10.0 | 8.0 | 7.0 | 7.5 | 9.0 | 6.5 | 7.5 | 8.5 | 7.5 | 12.0 | 9.5 | 9.5 | 7.0 | 8.0 | 10.0 | 7.5 | 12.0 |
| 1.0 | 1.0 | 1.0 | 1.0 | 1.0 | 2.5 | 1.0 | 1.0 | 1.0 | 1.0 | 1.0 | 1.0 | 3.0 | 1.0 | 1.0 | 2.5 | 1.0 | 1.0 | 1.0 | 2.0 |
| 3.0 | 5.0 | 3.0 | 5.0 | 5.0 | 2.5 | 2.5 | 3.0 | 3.5 | 2.5 | 4.0 | 2.5 | 6.0 | 2.5 | 4.0 | 2.5 | 5.0 | 5.0 | 2.5 | 6.0 |
| 11.0 | 10.0 | 12.0 | 10.0 | 8.0 | 13.0 | 12.0 | 12.0 | 10.0 | 12.0 | 10.0 | 12.0 | 12.0 | 12.0 | 11.0 | 13.0 | 8.0 | 10.0 | 12.0 | 12.0 |
| 15.5 | 15.5 | 15.5 | 15.5 | 15.5 | 15.5 | 15.5 | 15.5 | 15.5 | 15.5 | 15.5 | 15.5 | 15.5 | 15.5 | 15.5 | 15.5 | 15.5 | 15.5 | 15.5 | 15.5 |
| 14.0 | 14.0 | 14.0 | 14.0 | 14.0 | 13.0 | 13.5 | 14.0 | 13.5 | 13.5 | 14.0 | 13.5 | 14.0 | 13.5 | 14.0 | 13.0 | 14.0 | 14.0 | 13.5 | 14.0 |
| 6.5 | 5.0 | 6.5 | 5.0 | 5.0 | 7.0 | 7.5 | 6.5 | 6.5 | 7.5 | 5.5 | 7.5 | 6.0 | 9.5 | 5.5 | 7.0 | 5.0 | 5.0 | 7.5 | 6.0 |
| 15.5 | 15.5 | 15.5 | 15.5 | 15.5 | 15.5 | 15.5 | 15.5 | 15.5 | 15.5 | 15.5 | 15.5 | 15.5 | 15.5 | 15.5 | 15.5 | 15.5 | 15.5 | 15.5 | 15.5 |
| 5.0 | 7.5 | 5.0 | 7.5 | 10.5 | 2.5 | 4.0 | 4.5 | 9.0 | 4.0 | 7.0 | 4.5 | 3.0 | 4.0 | 7.0 | 2.5 | 10.5 | 7.5 | 5.0 | 3.5 |
| 10.0 | 7.5 | 8.0 | 7.5 | 10.5 | 10.5 | 10.5 | 9.0 | 11.5 | 10.5 | 11.0 | 10.5 | 3.0 | 6.5 | 8.0 | 10.5 | 10.5 | 7.5 | 10.5 | 3.5 |
| 4.0 | 3.0 | 4.0 | 3.0 | 2.0 | 7.0 | 5.0 | 4.5 | 2.0 | 5.0 | 3.0 | 4.5 | 10.0 | 5.0 | 3.0 | 7.0 | 2.0 | 3.0 | 4.0 | 8.0 |

**Table S3:** Rankings obtained after comparing 15 fingerprints using non-weighted extended similarity indices. Fingerprints with length m = 100.

| **Ref** | **eAC** | **eBUB** | **eCT1** | **eCT2** | **eCT3** | **eCT4** | **eFai** | **eGK** | **eGle** | **eHD** | **eJT** | **eJa0** | **eJa** | **eRG** | **eRR** | **eRT** | **eSM** | **eSS1** | **eSS2** |
| --- | --- | --- | --- | --- | --- | --- | --- | --- | --- | --- | --- | --- | --- | --- | --- | --- | --- | --- | --- |
| 15.0 | 16.0 | 16.0 | 16.0 | 16.0 | 12.0 | 14.0 | 15.0 | 15.0 | 14.0 | 16.0 | 14.0 | 12.0 | 14.0 | 15.0 | 12.0 | 16.0 | 16.0 | 15.0 | 15.0 |
| 1.0 | 2.0 | 2.0 | 2.0 | 2.0 | 2.0 | 2.0 | 1.0 | 1.0 | 2.0 | 2.0 | 2.0 | 3.0 | 2.0 | 2.0 | 2.0 | 2.0 | 2.0 | 2.0 | 2.0 |
| 13.0 | 9.5 | 10.0 | 9.5 | 12.0 | 13.0 | 12.0 | 12.0 | 13.0 | 11.0 | 10.0 | 12.0 | 5.0 | 9.0 | 9.0 | 13.0 | 10.0 | 9.5 | 13.0 | 9.0 |
| 14.0 | 11.5 | 14.0 | 11.5 | 13.5 | 15.5 | 15.5 | 14.0 | 14.0 | 15.0 | 14.0 | 15.5 | 6.5 | 15.0 | 14.0 | 15.5 | 13.5 | 11.5 | 14.0 | 10.5 |
| 4.0 | 6.0 | 5.0 | 6.0 | 6.0 | 3.0 | 3.0 | 4.0 | 4.0 | 4.0 | 6.0 | 3.0 | 2.0 | 4.0 | 4.0 | 3.0 | 6.0 | 6.0 | 3.0 | 3.0 |
| 16.0 | 15.0 | 15.0 | 15.0 | 15.0 | 15.5 | 15.5 | 16.0 | 16.0 | 13.0 | 15.0 | 15.5 | 13.0 | 12.0 | 16.0 | 15.5 | 15.0 | 15.0 | 16.0 | 16.0 |
| 7.0 | 7.0 | 8.0 | 7.0 | 7.0 | 7.0 | 8.0 | 7.0 | 6.0 | 12.0 | 7.0 | 8.0 | 10.0 | 13.0 | 7.0 | 7.0 | 7.0 | 7.0 | 7.0 | 7.0 |
| 12.0 | 9.5 | 13.0 | 9.5 | 8.0 | 14.0 | 13.0 | 13.0 | 8.0 | 16.0 | 8.0 | 13.0 | 16.0 | 16.0 | 11.0 | 14.0 | 8.0 | 9.5 | 12.0 | 13.0 |
| 9.0 | 14.0 | 9.0 | 14.0 | 11.0 | 4.0 | 6.0 | 10.0 | 9.0 | 7.0 | 12.0 | 7.0 | 15.0 | 7.0 | 13.0 | 4.0 | 12.0 | 14.0 | 8.0 | 14.0 |
| 8.0 | 8.0 | 7.0 | 8.0 | 9.0 | 9.5 | 11.0 | 8.0 | 11.0 | 8.0 | 9.0 | 9.0 | 4.0 | 8.0 | 8.0 | 9.5 | 9.0 | 8.0 | 9.0 | 6.0 |
| 5.0 | 5.0 | 4.0 | 5.0 | 3.0 | 7.0 | 4.0 | 5.0 | 5.0 | 3.0 | 4.0 | 4.0 | 11.0 | 3.0 | 6.0 | 7.0 | 3.0 | 5.0 | 4.0 | 8.0 |
| 3.0 | 1.0 | 3.0 | 1.0 | 1.0 | 9.5 | 5.0 | 3.0 | 3.0 | 5.0 | 1.0 | 5.0 | 1.0 | 5.0 | 1.0 | 9.5 | 1.0 | 1.0 | 5.0 | 1.0 |
| 11.0 | 11.5 | 11.0 | 11.5 | 13.5 | 5.0 | 9.0 | 9.0 | 12.0 | 10.0 | 13.0 | 11.0 | 6.5 | 10.0 | 10.0 | 5.0 | 13.5 | 11.5 | 11.0 | 10.5 |
| 2.0 | 3.5 | 1.0 | 3.5 | 4.5 | 1.0 | 1.0 | 2.0 | 2.0 | 1.0 | 3.0 | 1.0 | 8.5 | 1.0 | 3.0 | 1.0 | 4.5 | 3.5 | 1.0 | 4.5 |
| 10.0 | 13.0 | 12.0 | 13.0 | 10.0 | 7.0 | 10.0 | 11.0 | 10.0 | 9.0 | 11.0 | 10.0 | 14.0 | 11.0 | 12.0 | 7.0 | 11.0 | 13.0 | 10.0 | 12.0 |
| 6.00 | 3.5 | 6.0 | 3.5 | 4.5 | 11.0 | 7.0 | 6.0 | 7.0 | 6.0 | 5.0 | 6.0 | 8.5 | 6.0 | 5.0 | 11.0 | 4.5 | 3.5 | 6.0 | 4.5 |

**Table S4:** Rankings obtained after comparing 15 fingerprints using non-weighted extended similarity indices. Fingerprints with length m = 1000.

| **Ref** | **eAC** | **eBUB** | **eCT1** | **eCT2** | **eCT3** | **eCT4** | **eFai** | **eGK** | **eGle** | **eHD** | **eJT** | **eJa0** | **eJa** | **eRG** | **eRR** | **eRT** | **eSM** | **eSS1** | **eSS2** |
| --- | --- | --- | --- | --- | --- | --- | --- | --- | --- | --- | --- | --- | --- | --- | --- | --- | --- | --- | --- |
| 4.0 | 4.0 | 6.0 | 4.0 | 2.0 | 9.0 | 7.0 | 8.0 | 3.0 | 6.0 | 2.0 | 5.0 | 12.0 | 10.0 | 4.0 | 9.0 | 2.0 | 4.0 | 5.0 | 8.0 |
| 5.0 | 8.0 | 2.0 | 8.0 | 8.0 | 5.5 | 3.0 | 6.0 | 8.0 | 2.0 | 7.0 | 3.0 | 7.0 | 2.0 | 8.0 | 5.5 | 7.0 | 8.0 | 3.0 | 7.0 |
| 3.0 | 7.0 | 5.0 | 7.0 | 6.0 | 4.0 | 4.0 | 4.0 | 5.0 | 5.0 | 6.0 | 4.0 | 5.0 | 4.0 | 7.0 | 4.0 | 6.0 | 7.0 | 4.0 | 6.0 |
| 1.0 | 1.0 | 1.0 | 1.0 | 1.0 | 1.0 | 1.0 | 1.0 | 1.0 | 1.0 | 1.0 | 1.0 | 4.0 | 1.0 | 1.0 | 1.0 | 1.0 | 1.0 | 1.0 | 2.0 |
| 13.0 | 16.0 | 15.0 | 16.0 | 11.0 | 13.0 | 13.0 | 15.0 | 7.0 | 15.0 | 12.0 | 13.0 | 16.0 | 15.0 | 16.0 | 13.0 | 13.0 | 16.0 | 12.0 | 16.0 |
| 6.0 | 6.0 | 4.0 | 6.0 | 13.0 | 3.0 | 5.0 | 3.0 | 13.0 | 4.0 | 10.0 | 7.0 | 1.0 | 3.0 | 6.0 | 3.0 | 10.0 | 6.0 | 8.0 | 1.0 |
| 10.0 | 10.0 | 9.0 | 10.0 | 10.0 | 10.0 | 10.0 | 9.5 | 10.0 | 8.0 | 9.0 | 8.0 | 9.0 | 6.0 | 10.0 | 10.0 | 9.0 | 10.0 | 9.0 | 10.0 |
| 14.0 | 14.0 | 14.0 | 14.0 | 14.0 | 14.0 | 14.0 | 13.0 | 14.0 | 13.0 | 15.0 | 14.0 | 14.0 | 14.0 | 14.0 | 14.0 | 14.0 | 14.0 | 14.0 | 14.0 |
| 16.0 | 15.0 | 16.0 | 15.0 | 16.0 | 15.0 | 16.0 | 14.0 | 15.0 | 16.0 | 16.0 | 16.0 | 10.0 | 16.0 | 15.0 | 15.0 | 16.0 | 15.0 | 16.0 | 12.0 |
| 15.0 | 12.5 | 13.0 | 12.5 | 12.0 | 16.0 | 15.0 | 16.0 | 16.0 | 14.0 | 13.0 | 15.0 | 13.0 | 13.0 | 13.0 | 16.0 | 12.0 | 12.5 | 15.0 | 13.0 |
| 11.0 | 9.0 | 10.0 | 9.0 | 9.0 | 11.0 | 11.0 | 9.5 | 11.0 | 9.0 | 8.0 | 9.0 | 8.0 | 8.0 | 9.0 | 11.0 | 8.0 | 9.0 | 11.0 | 9.0 |
| 7.0 | 5.0 | 8.0 | 5.0 | 5.0 | 8.0 | 8.0 | 5.0 | 6.0 | 12.0 | 5.0 | 11.0 | 3.0 | 12.0 | 5.0 | 8.0 | 5.0 | 5.0 | 7.0 | 4.0 |
| 8.0 | 2.0 | 7.0 | 2.0 | 4.0 | 12.0 | 12.0 | 7.0 | 9.0 | 11.0 | 4.0 | 10.0 | 2.0 | 9.0 | 3.0 | 12.0 | 4.0 | 2.0 | 10.0 | 3.0 |
| 2.0 | 3.0 | 3.0 | 3.0 | 3.0 | 2.0 | 2.0 | 2.0 | 2.0 | 3.0 | 3.0 | 2.0 | 6.0 | 5.0 | 2.0 | 2.0 | 3.0 | 3.0 | 2.0 | 5.0 |
| 12.0 | 12.5 | 12.0 | 12.5 | 15.0 | 7.0 | 9.0 | 12.0 | 12.0 | 10.0 | 14.0 | 12.0 | 11.0 | 7.0 | 12.0 | 7.0 | 15.0 | 12.5 | 13.0 | 11.0 |
| 9.0 | 11.0 | 11.0 | 11.0 | 7.0 | 5.5 | 6.0 | 11.0 | 4.0 | 7.0 | 11.0 | 6.0 | 15.0 | 11.0 | 11.0 | 5.5 | 11.0 | 11.0 | 6.0 | 15.0 |

**Table S5:** Rankings obtained after comparing 15 fingerprints using non-weighted extended similarity indices. Fingerprints with length m = 100000.

| **Ref** | **eAC** | **eBUB** | **eCT1** | **eCT2** | **eCT3** | **eCT4** | **eFai** | **eGK** | **eGle** | **eHD** | **eJT** | **eJa0** | **eJa** | **eRG** | **eRR** | **eRT** | **eSM** | **eSS1** | **eSS2** |
| --- | --- | --- | --- | --- | --- | --- | --- | --- | --- | --- | --- | --- | --- | --- | --- | --- | --- | --- | --- |
| 4.0 | 4.0 | 4.0 | 4.0 | 5.0 | 4.0 | 3.0 | 4.0 | 6.0 | 4.0 | 6.0 | 4.0 | 6.0 | 4.0 | 4.0 | 4.0 | 6.0 | 4.0 | 4.0 | 6.0 |
| 13.0 | 13.0 | 14.0 | 13.0 | 12.0 | 14.0 | 14.0 | 14.0 | 11.0 | 14.0 | 13.0 | 14.0 | 15.0 | 13.0 | 13.0 | 14.0 | 13.0 | 13.0 | 14.0 | 15.0 |
| 15.0 | 16.0 | 15.0 | 16.0 | 16.0 | 15.0 | 15.0 | 15.0 | 16.0 | 15.0 | 16.0 | 15.0 | 12.0 | 14.0 | 16.0 | 15.0 | 16.0 | 16.0 | 15.0 | 14.0 |
| 12.0 | 12.0 | 12.0 | 12.0 | 13.0 | 10.0 | 12.0 | 11.0 | 13.0 | 10.0 | 12.0 | 12.0 | 8.0 | 10.0 | 12.0 | 10.0 | 12.0 | 12.0 | 12.0 | 9.0 |
| 16.0 | 15.0 | 16.0 | 15.0 | 15.0 | 16.0 | 16.0 | 16.0 | 15.0 | 16.0 | 15.0 | 16.0 | 16.0 | 16.0 | 15.0 | 16.0 | 15.0 | 15.0 | 16.0 | 16.0 |
| 10.0 | 8.0 | 9.0 | 8.0 | 11.0 | 8.0 | 9.0 | 8.0 | 12.0 | 9.0 | 11.0 | 10.0 | 3.0 | 9.0 | 8.0 | 8.0 | 11.0 | 8.0 | 11.0 | 4.0 |
| 1.0 | 1.0 | 1.0 | 1.0 | 1.0 | 1.0 | 1.0 | 1.0 | 1.0 | 1.0 | 1.0 | 1.0 | 1.0 | 1.0 | 1.0 | 1.0 | 1.0 | 1.0 | 1.0 | 1.0 |
| 8.0 | 10.0 | 5.0 | 10.0 | 10.0 | 6.0 | 6.0 | 9.0 | 10.0 | 2.0 | 10.0 | 5.0 | 9.0 | 2.0 | 10.0 | 6.0 | 10.0 | 10.0 | 6.0 | 8.0 |
| 3.0 | 3.0 | 3.0 | 3.0 | 7.0 | 2.0 | 5.0 | 3.0 | 7.0 | 5.0 | 5.0 | 6.0 | 2.0 | 5.0 | 3.0 | 2.0 | 5.0 | 3.0 | 5.0 | 2.0 |
| 7.0 | 7.0 | 7.0 | 7.0 | 8.0 | 9.0 | 8.0 | 6.0 | 8.0 | 8.0 | 7.0 | 8.0 | 4.0 | 7.0 | 7.0 | 9.0 | 7.0 | 7.0 | 9.0 | 5.0 |
| 2.0 | 2.0 | 2.0 | 2.0 | 2.0 | 3.0 | 2.0 | 2.0 | 2.0 | 3.0 | 2.0 | 2.0 | 5.0 | 3.0 | 2.0 | 3.0 | 2.0 | 2.0 | 2.0 | 3.0 |
| 9.0 | 9.0 | 8.0 | 9.0 | 9.0 | 5.0 | 7.0 | 7.0 | 9.0 | 7.0 | 9.0 | 7.0 | 7.0 | 6.0 | 9.0 | 5.0 | 9.0 | 9.0 | 7.0 | 7.0 |
| 5.0 | 6.0 | 6.0 | 6.0 | 3.0 | 7.0 | 4.0 | 5.0 | 4.0 | 6.0 | 3.0 | 3.0 | 11.0 | 8.0 | 6.0 | 7.0 | 3.0 | 6.0 | 3.0 | 11.0 |
| 14.0 | 14.0 | 13.0 | 14.0 | 14.0 | 11.0 | 13.0 | 13.0 | 14.0 | 12.0 | 14.0 | 13.0 | 14.0 | 11.0 | 14.0 | 11.0 | 14.0 | 14.0 | 13.0 | 13.0 |
| 11.0 | 11.0 | 11.0 | 11.0 | 6.0 | 13.0 | 11.0 | 12.0 | 5.0 | 11.0 | 8.0 | 11.0 | 13.0 | 12.0 | 11.0 | 13.0 | 8.0 | 11.0 | 10.0 | 12.0 |
| 6.0 | 5.0 | 10.0 | 5.0 | 4.0 | 12.0 | 10.0 | 10.0 | 3.0 | 13.0 | 4.0 | 9.0 | 10.0 | 15.0 | 5.0 | 12.0 | 4.0 | 5.0 | 8.0 | 10.0 |

**Figures S1-19.** Mean values of the extended similarity indices for different numbers of compared objects (*n*), fingerprint lengths (*m*) in weighted (w) *vs.* non-weighted (nw) definitions.

**
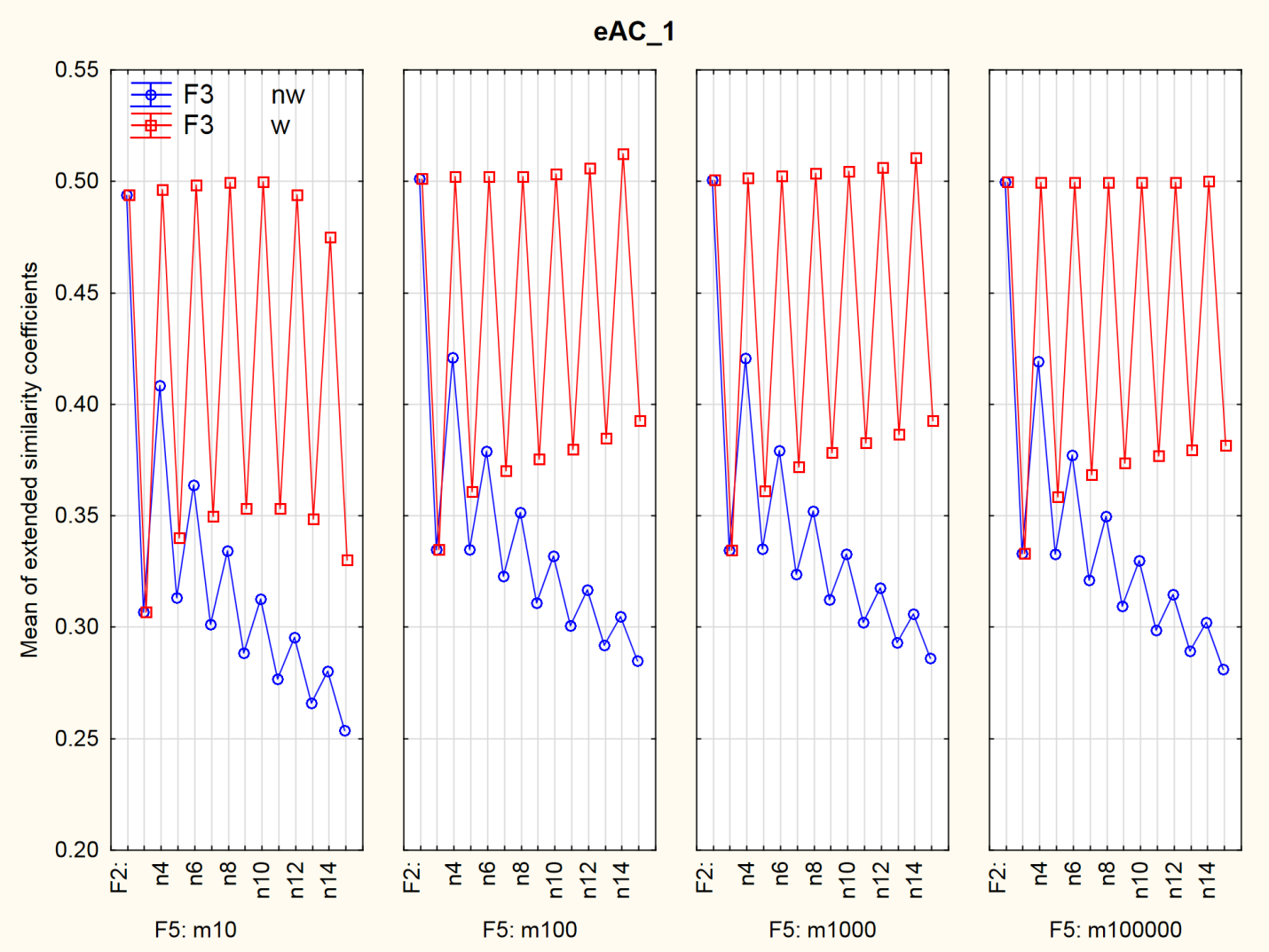
**

**
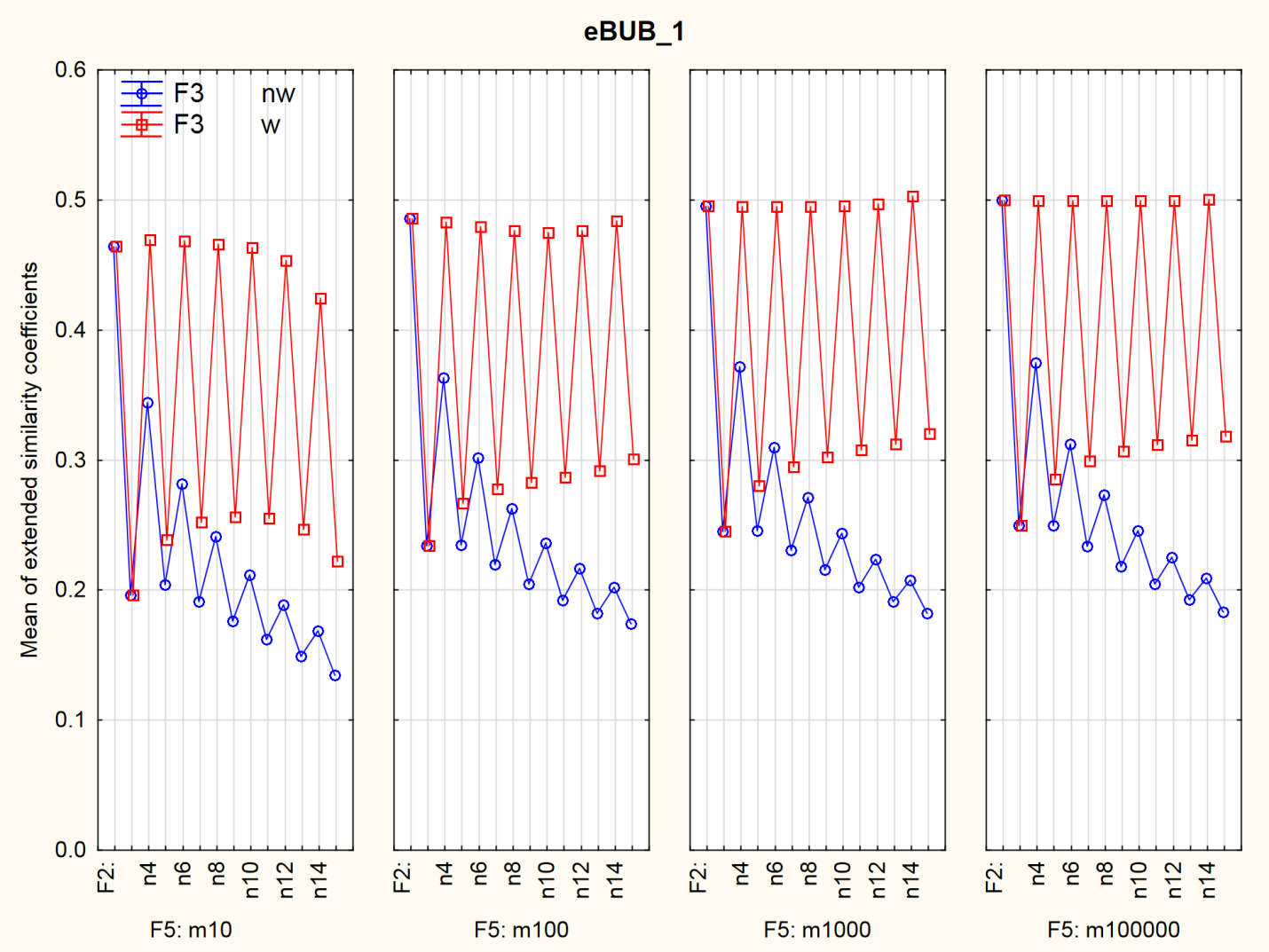
**

**
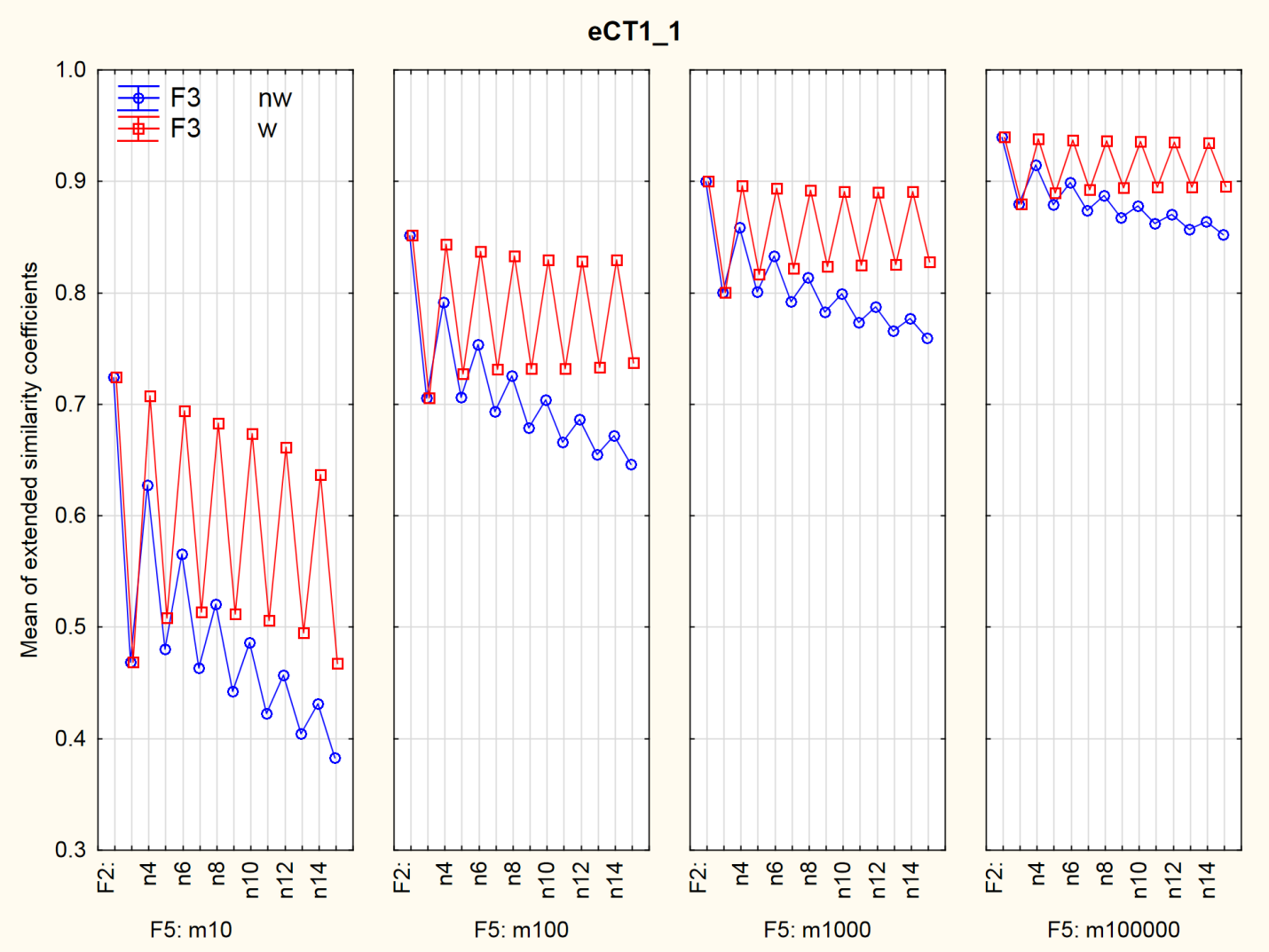
**

**
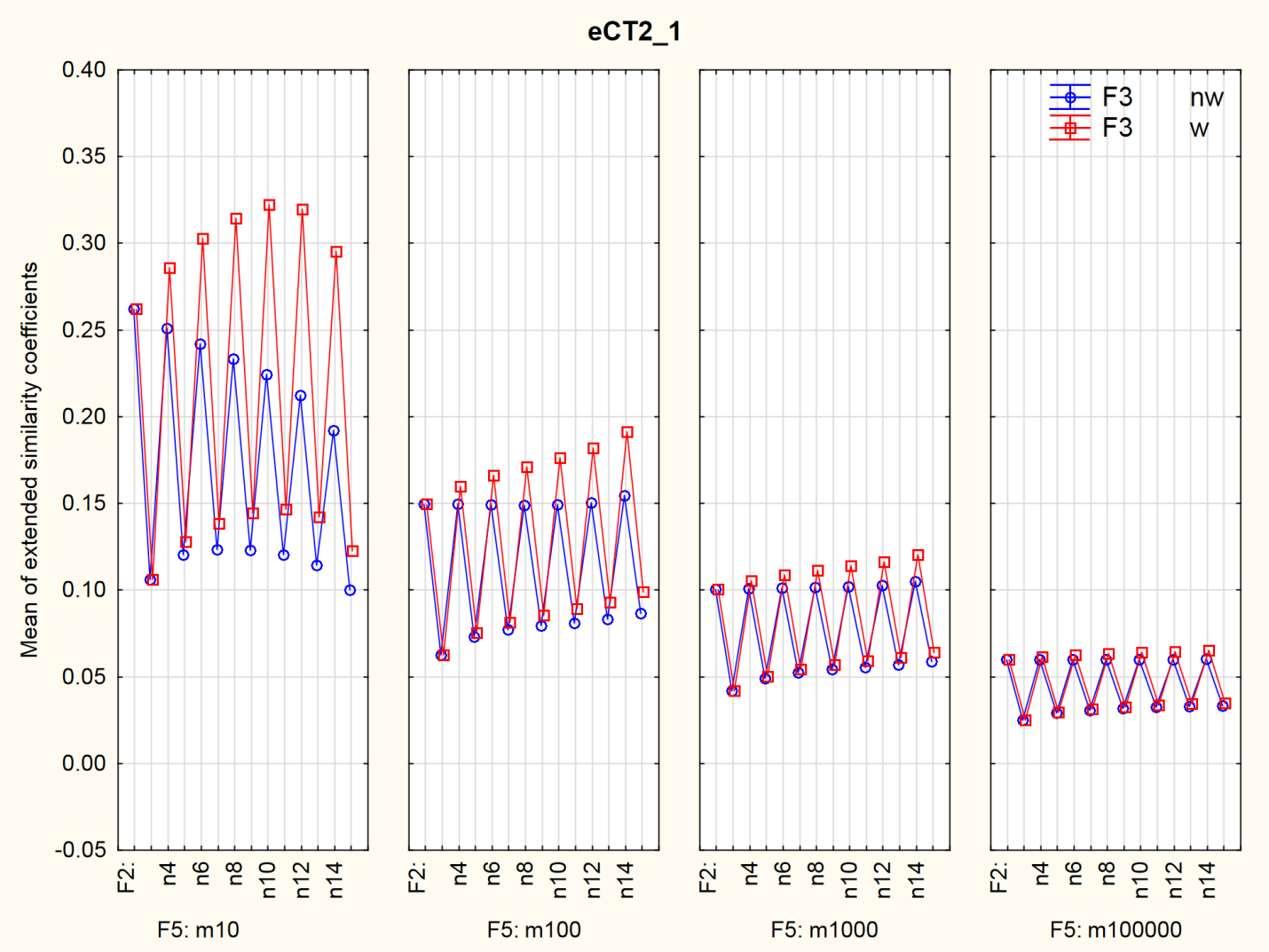

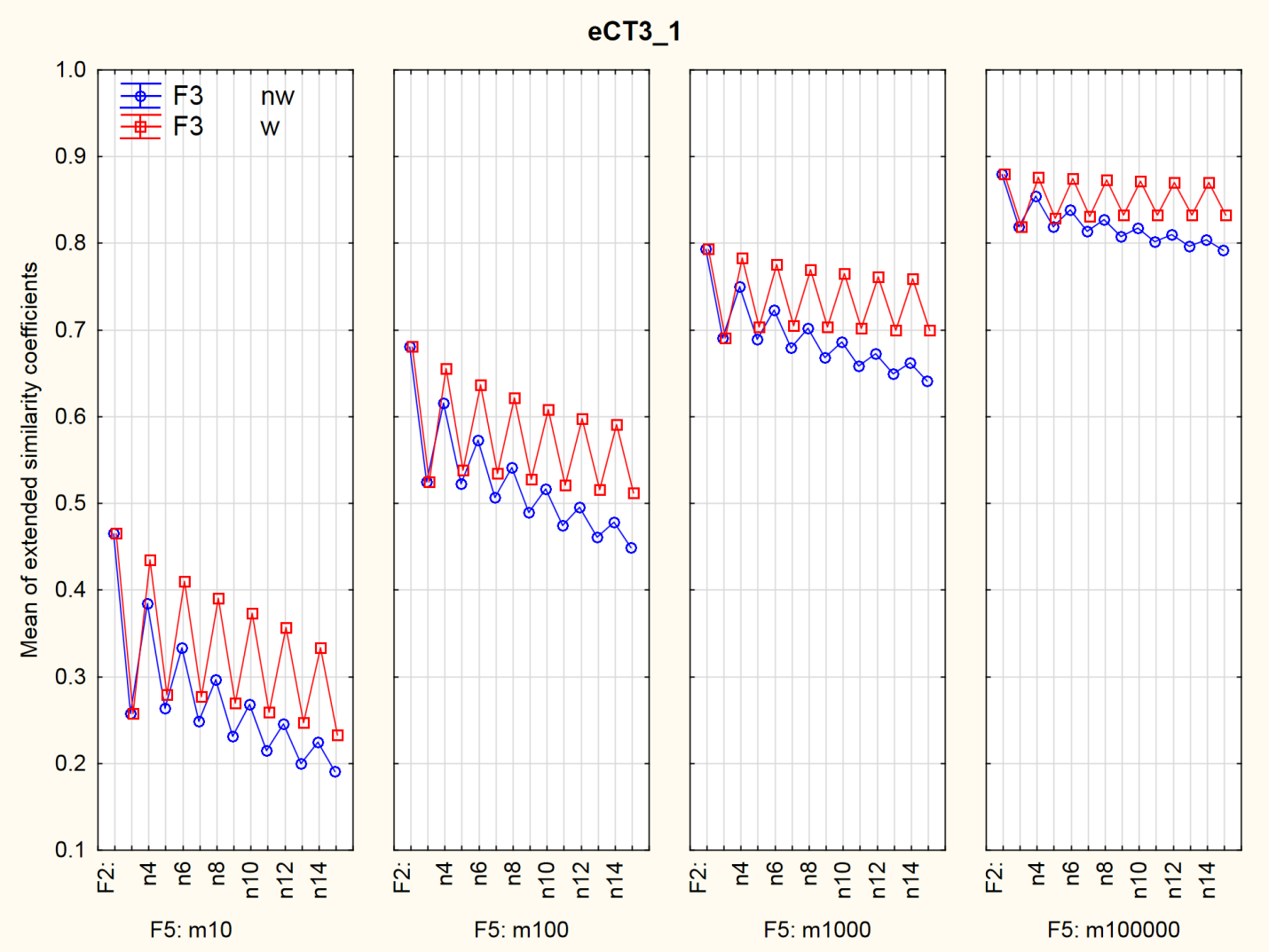

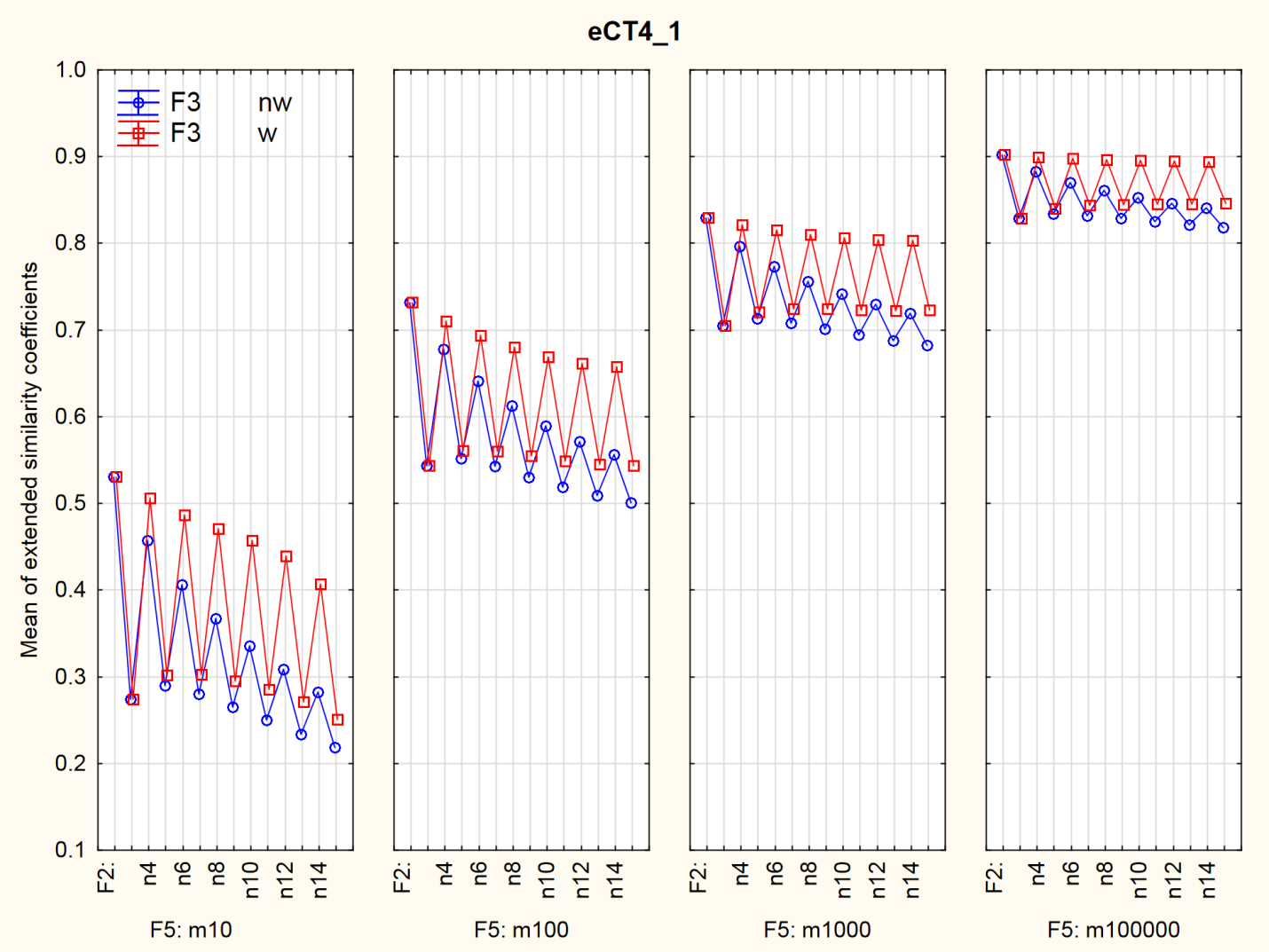

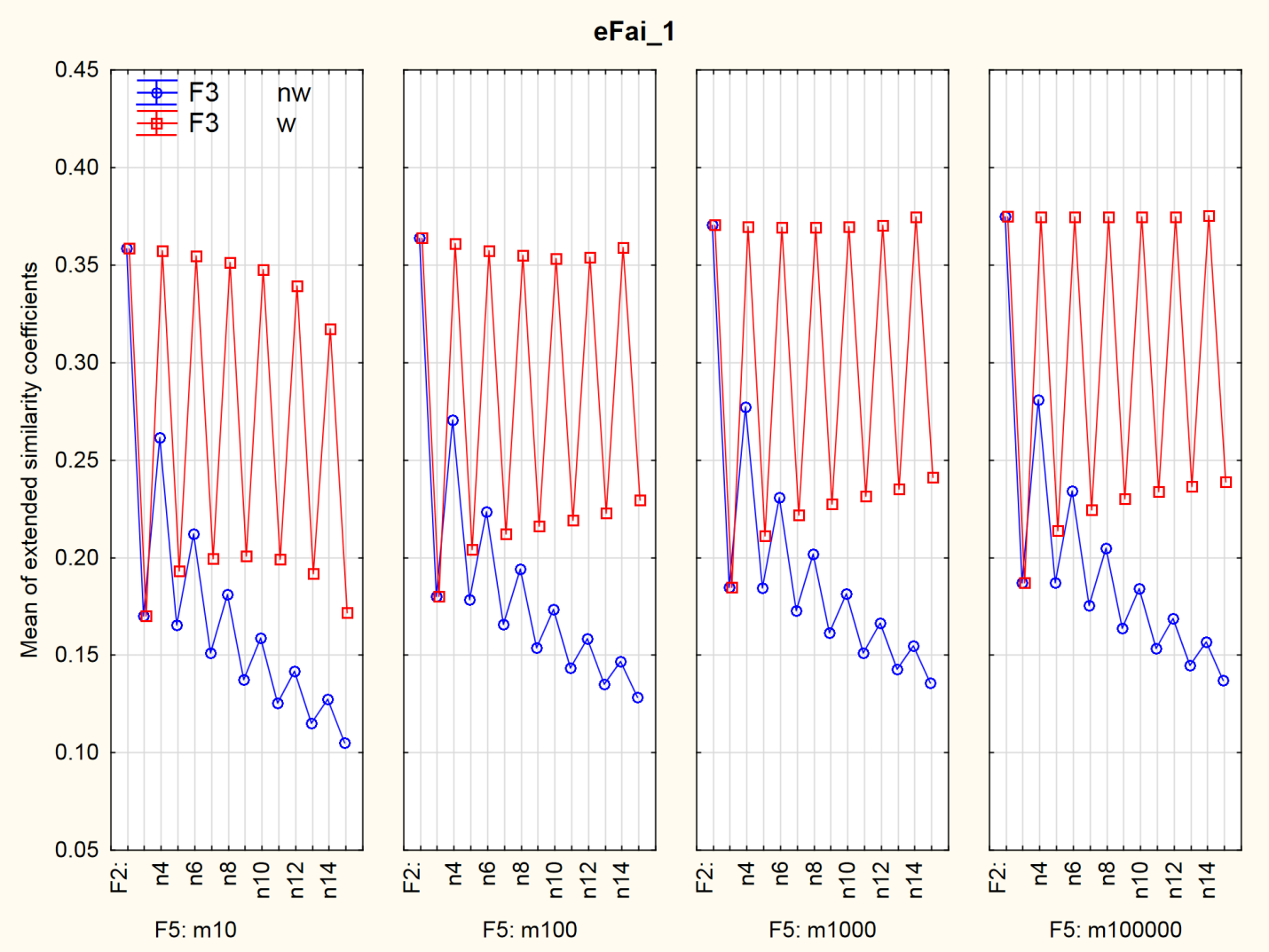

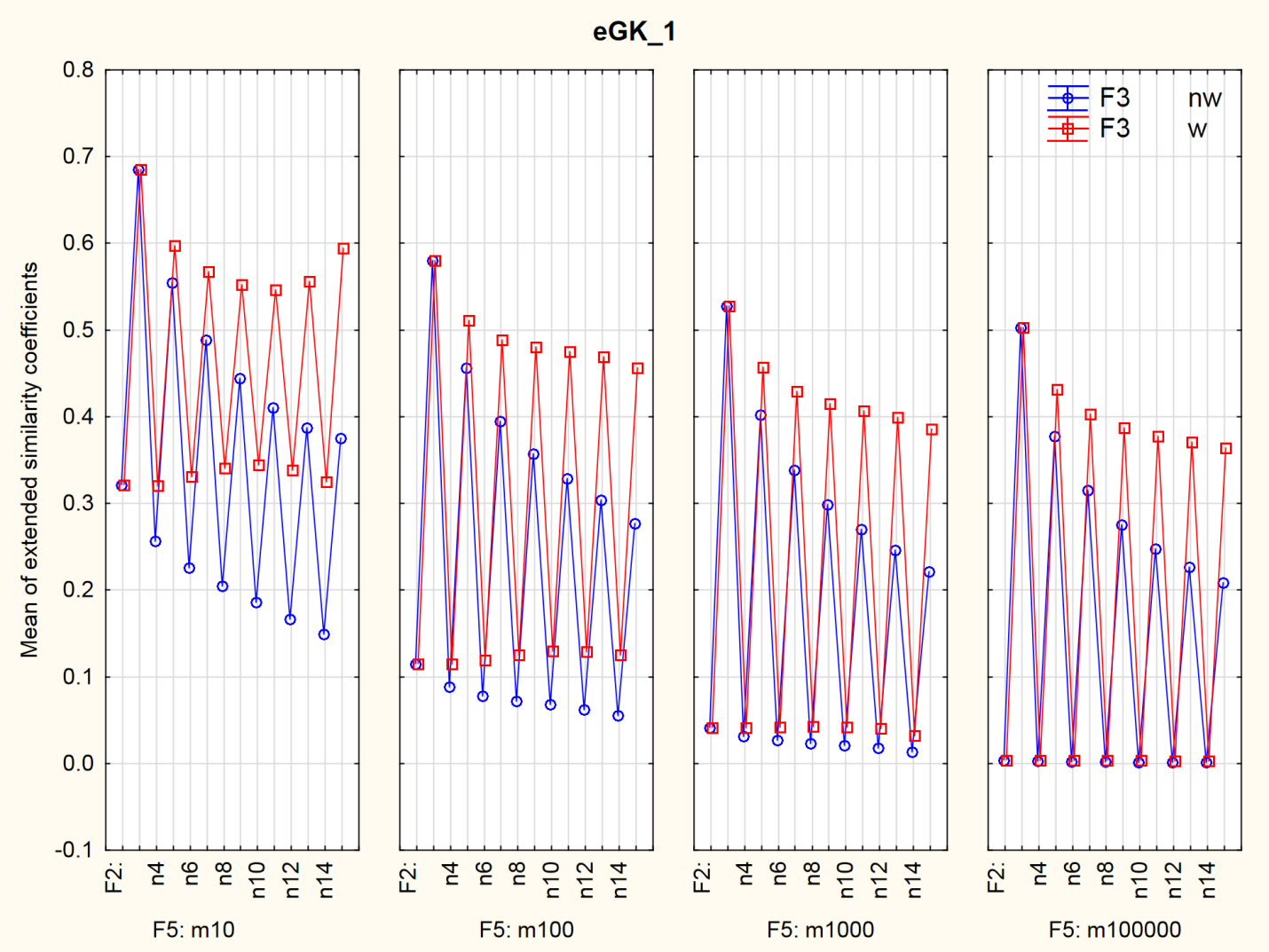

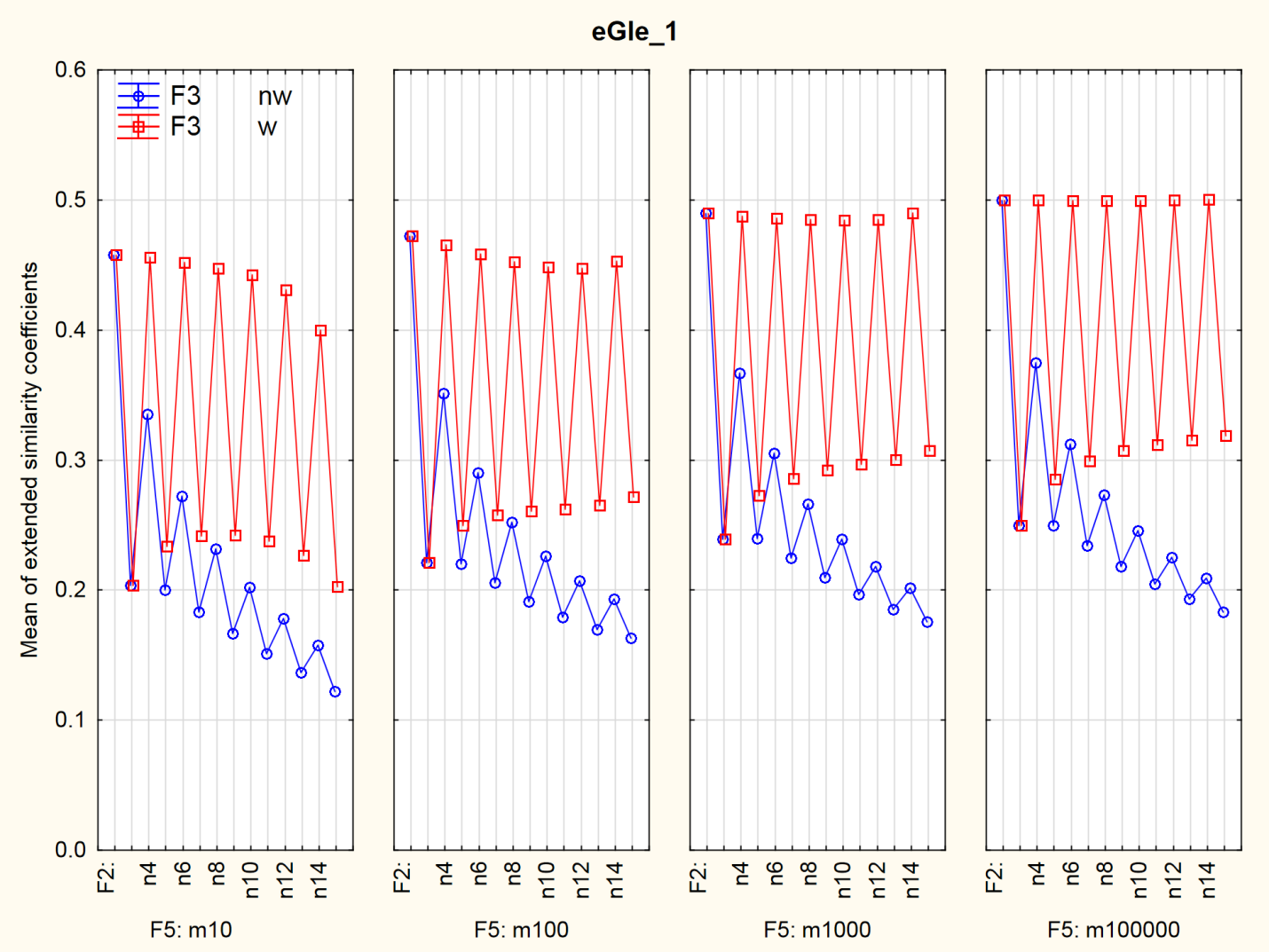

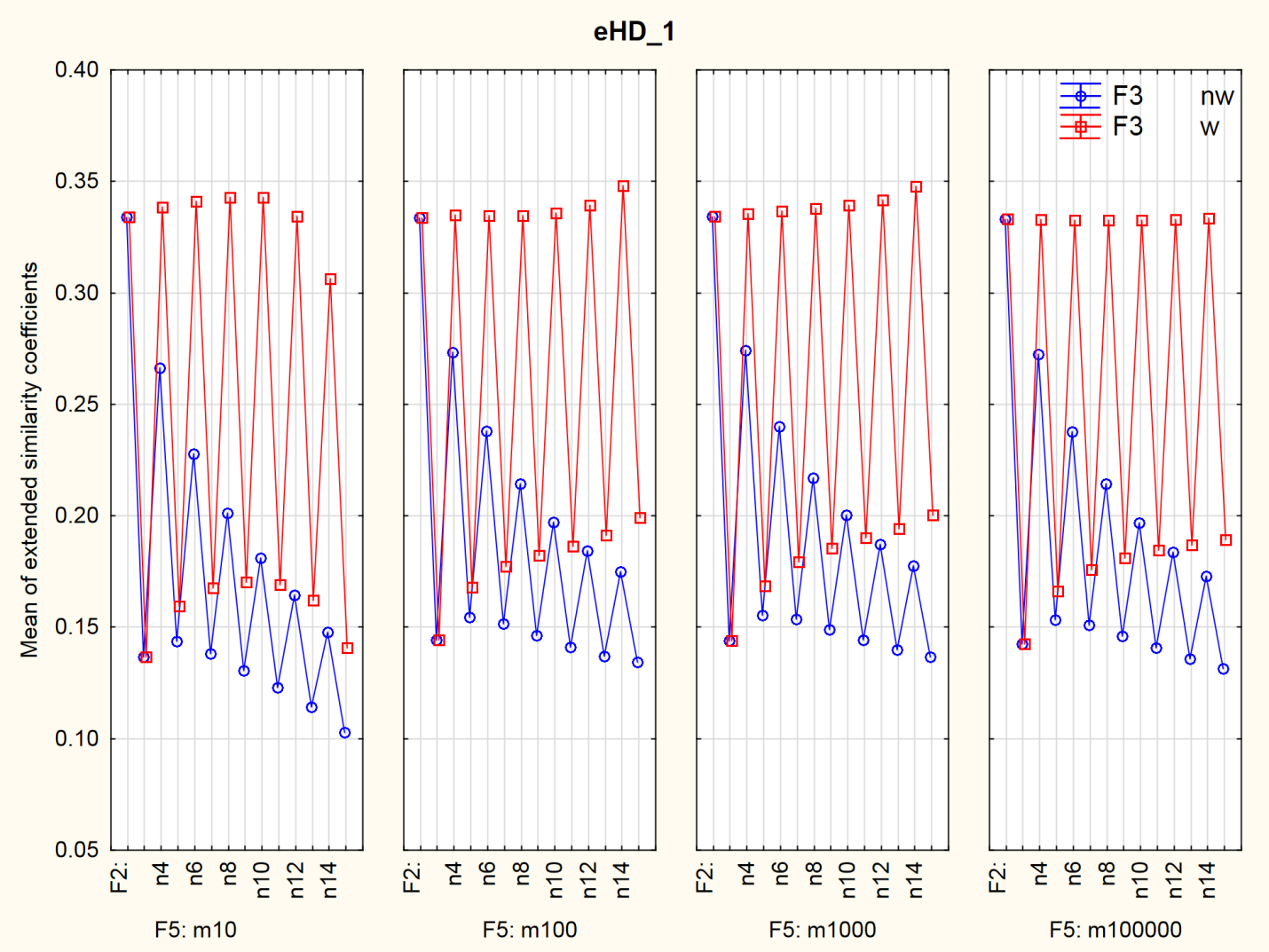

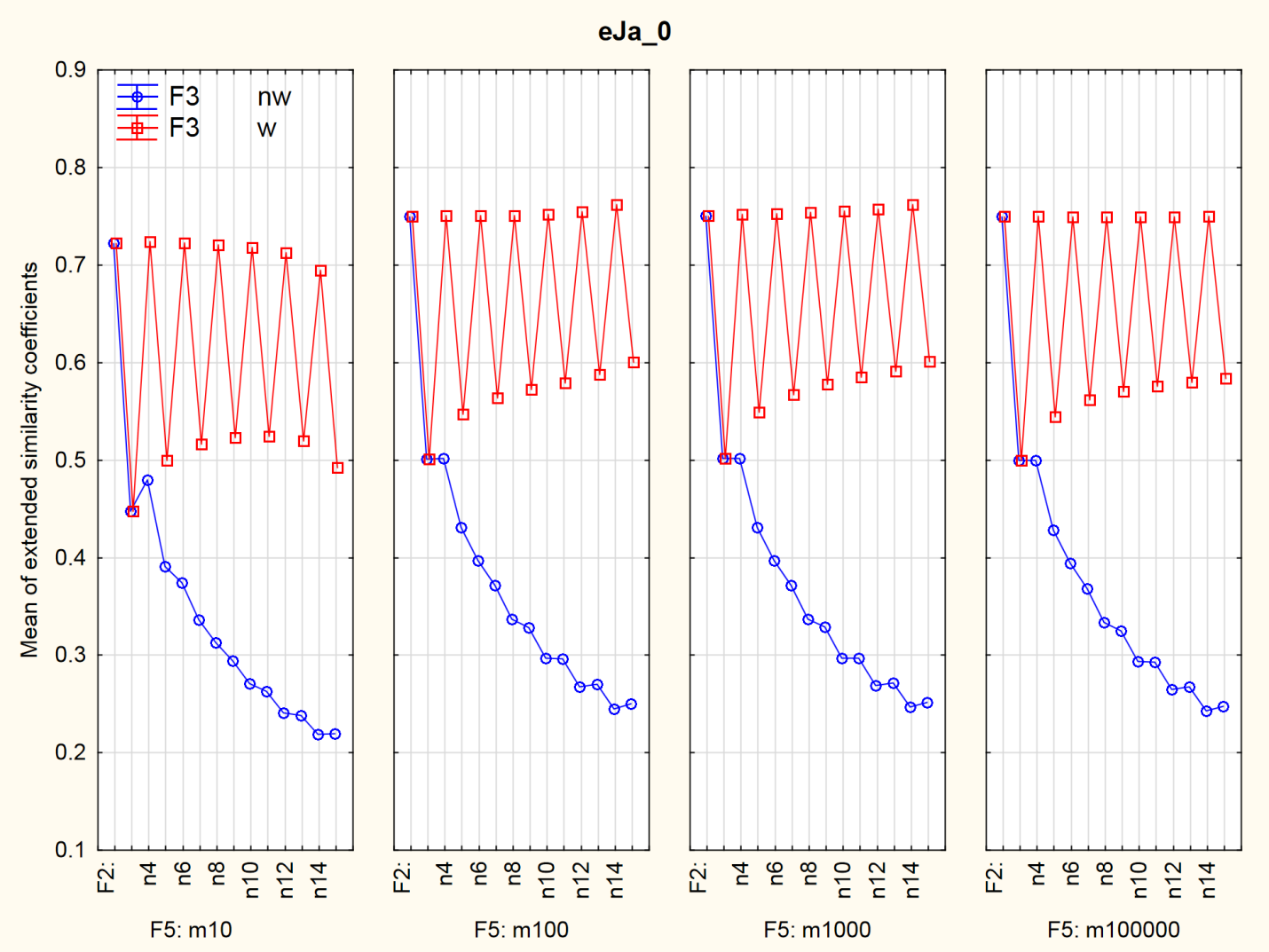

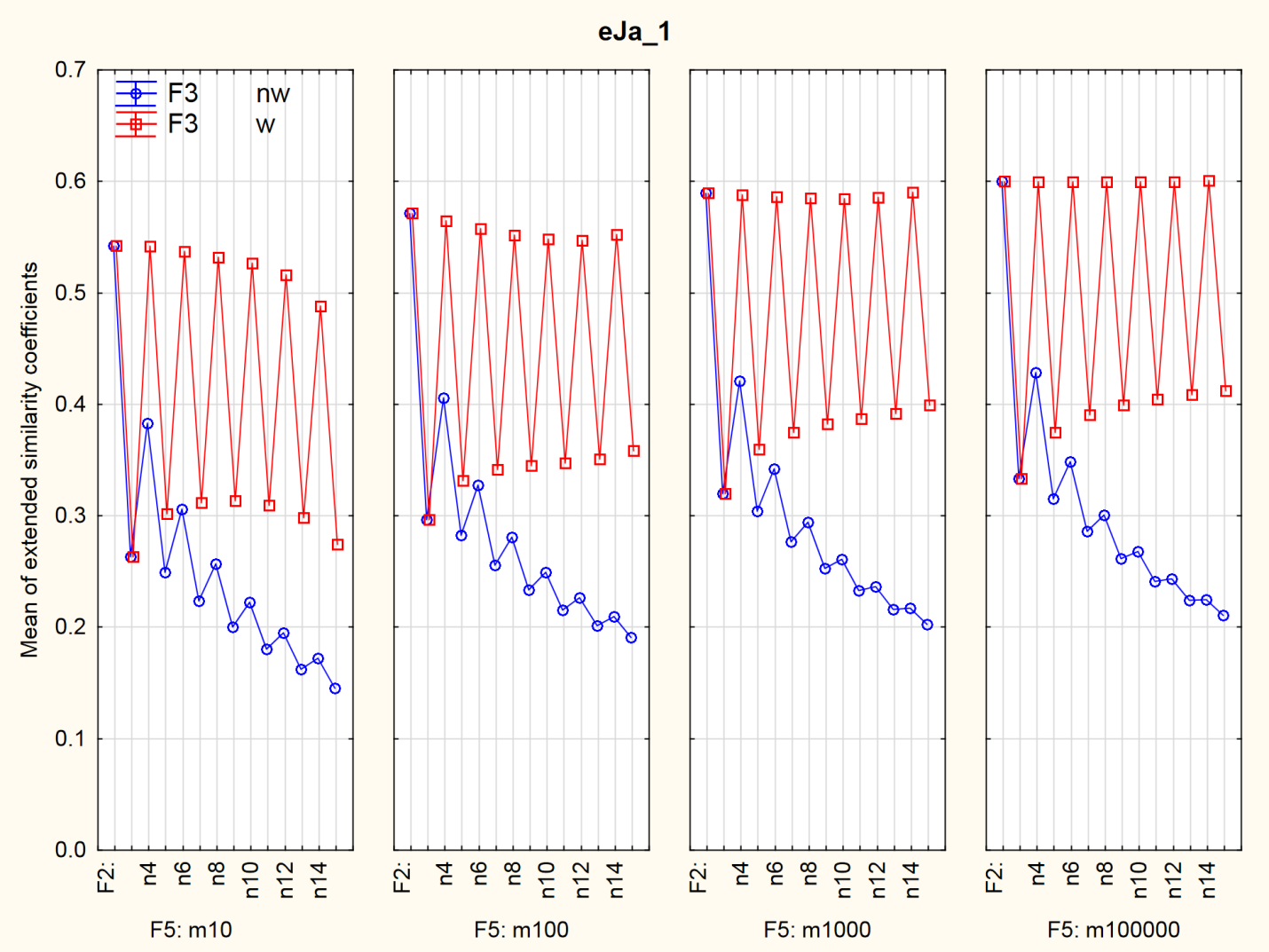

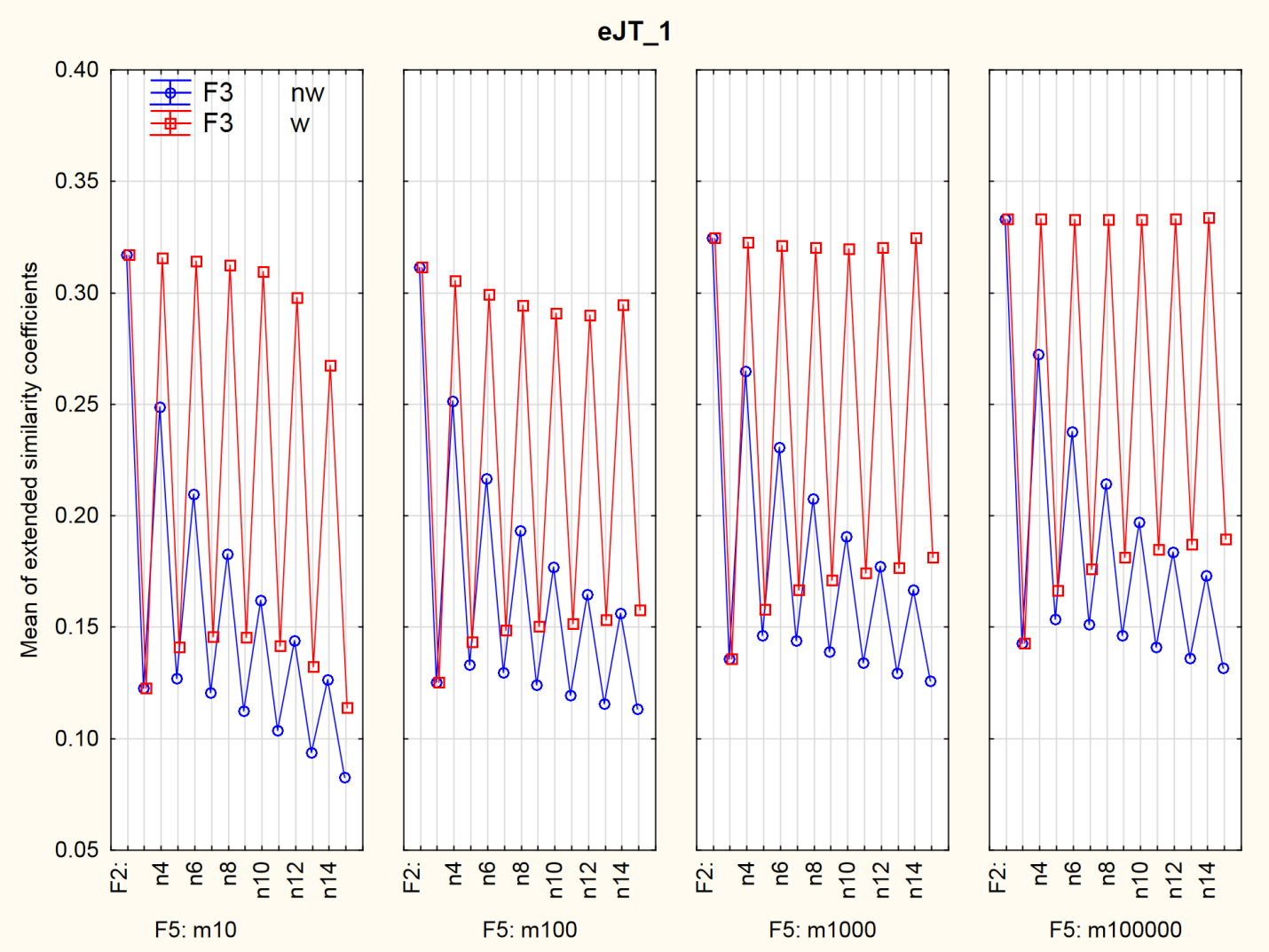

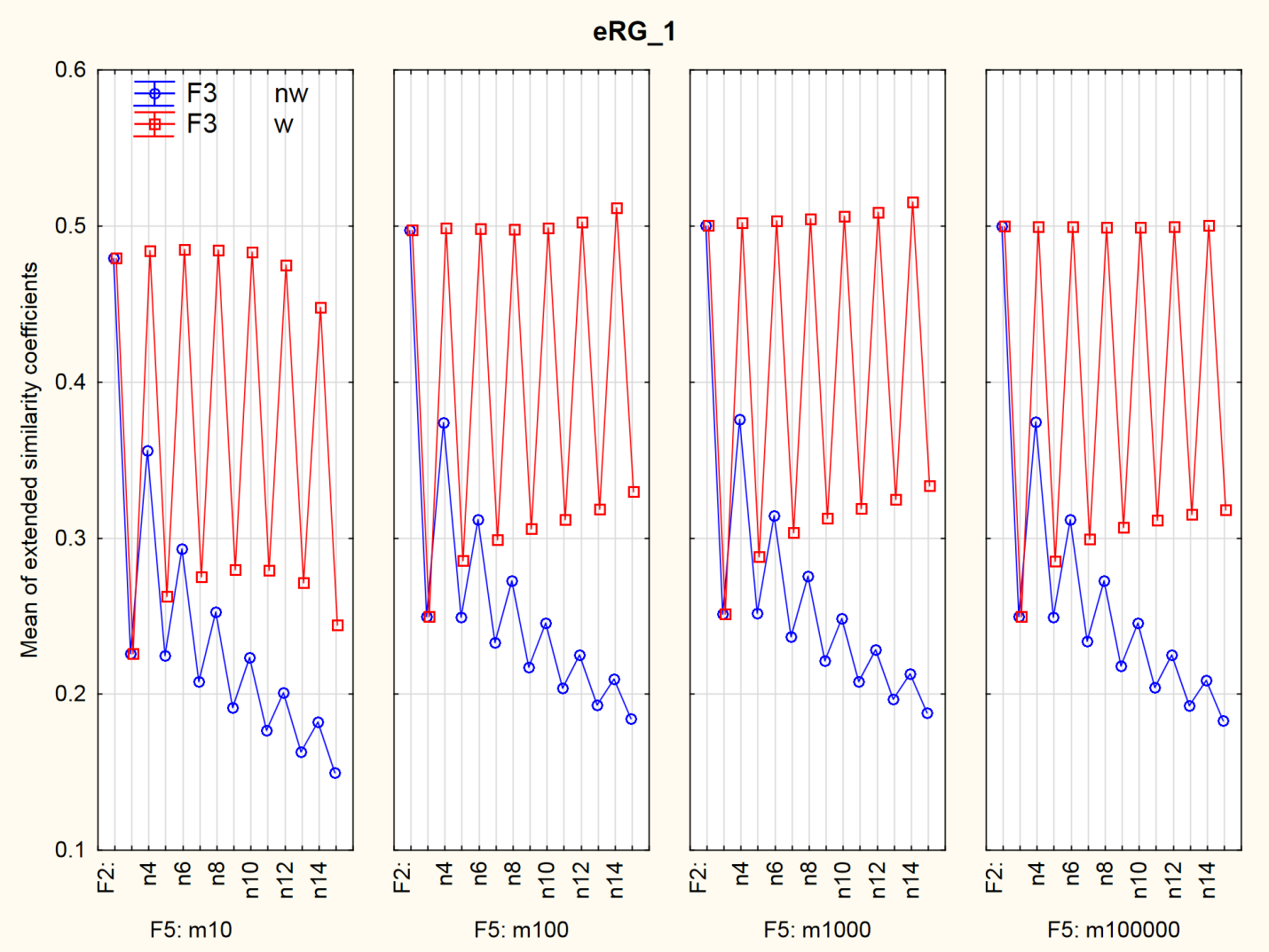

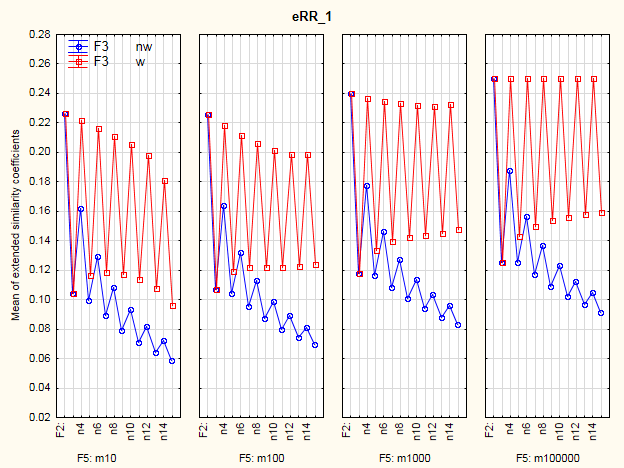

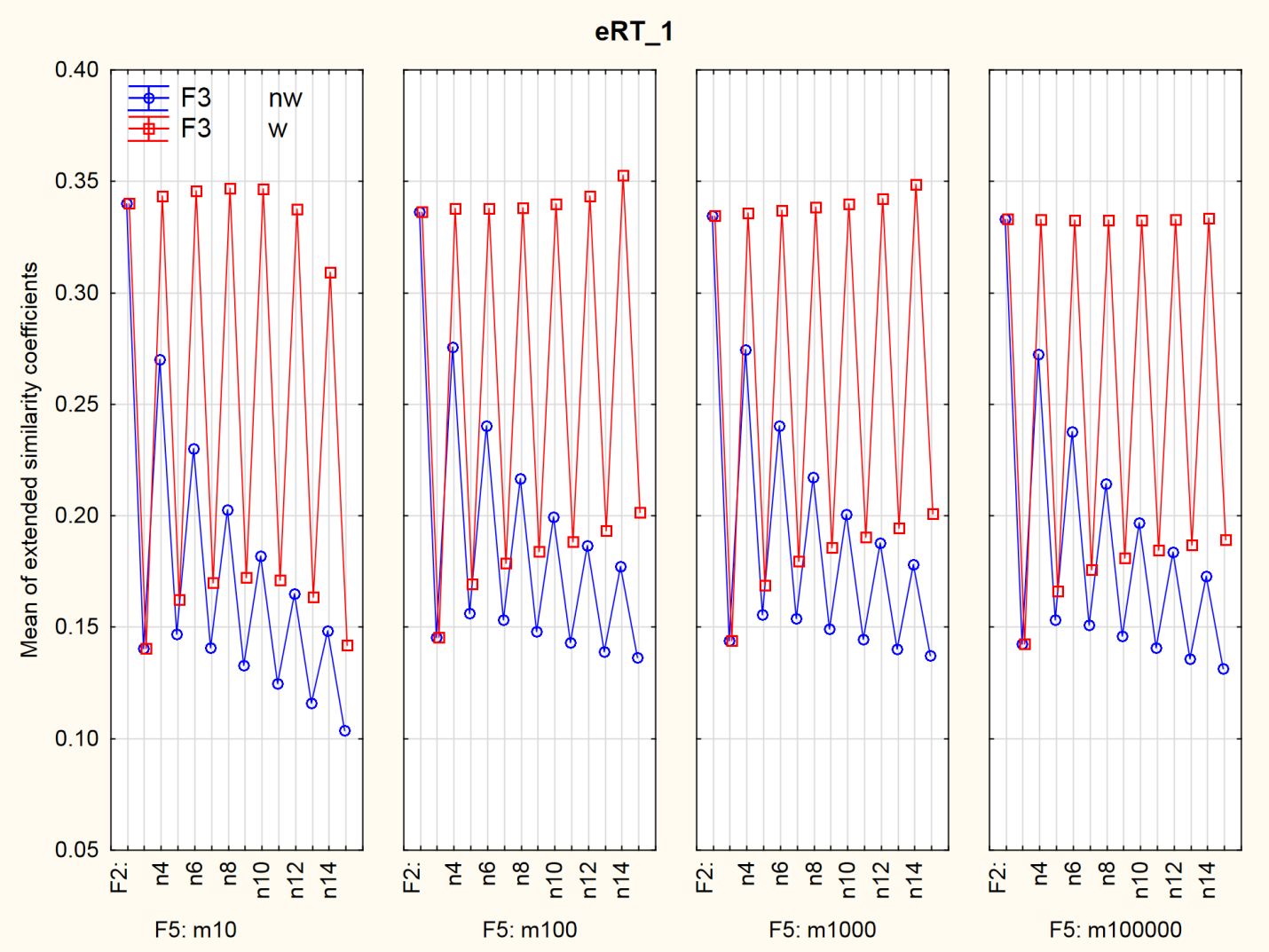

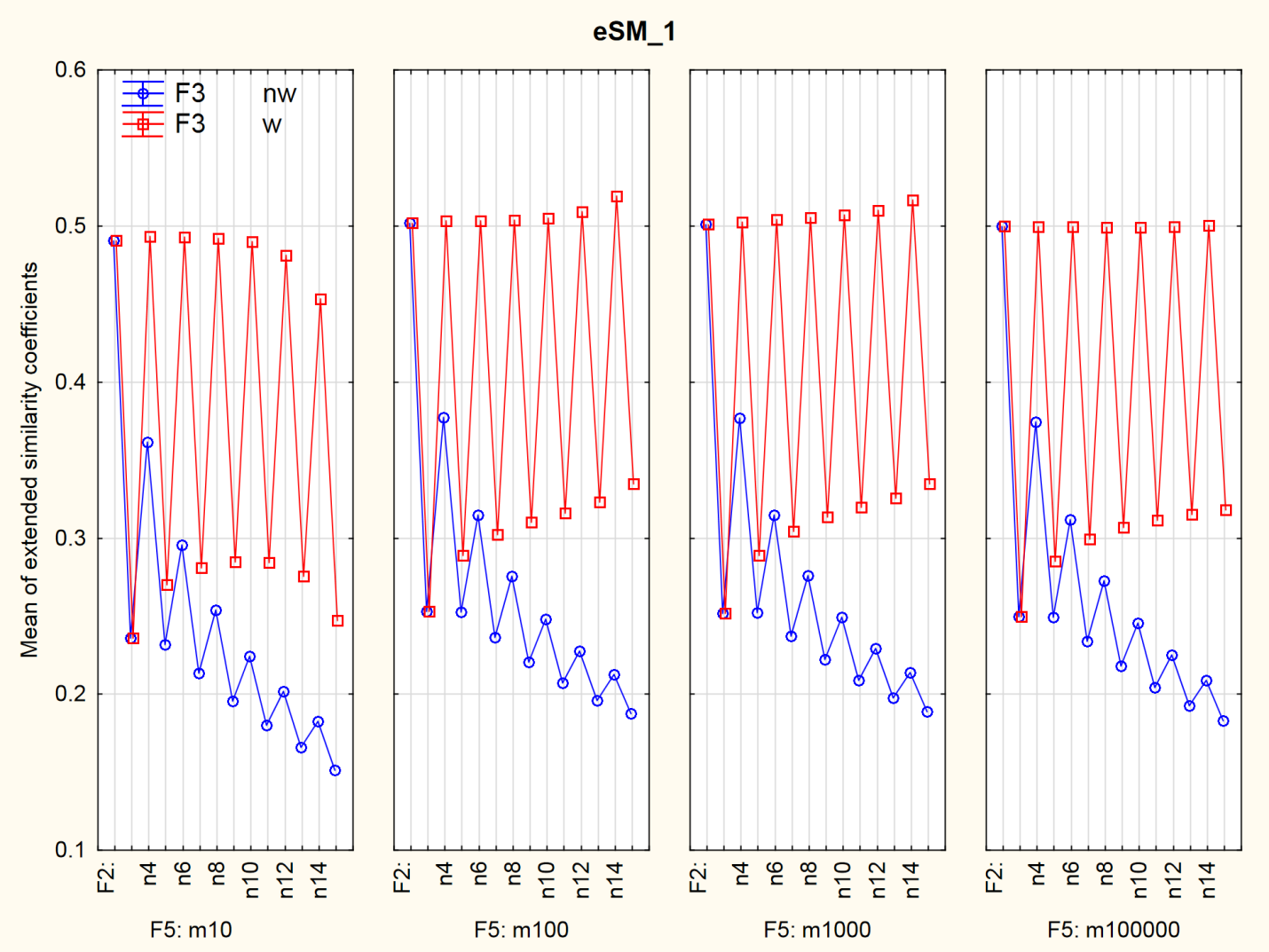

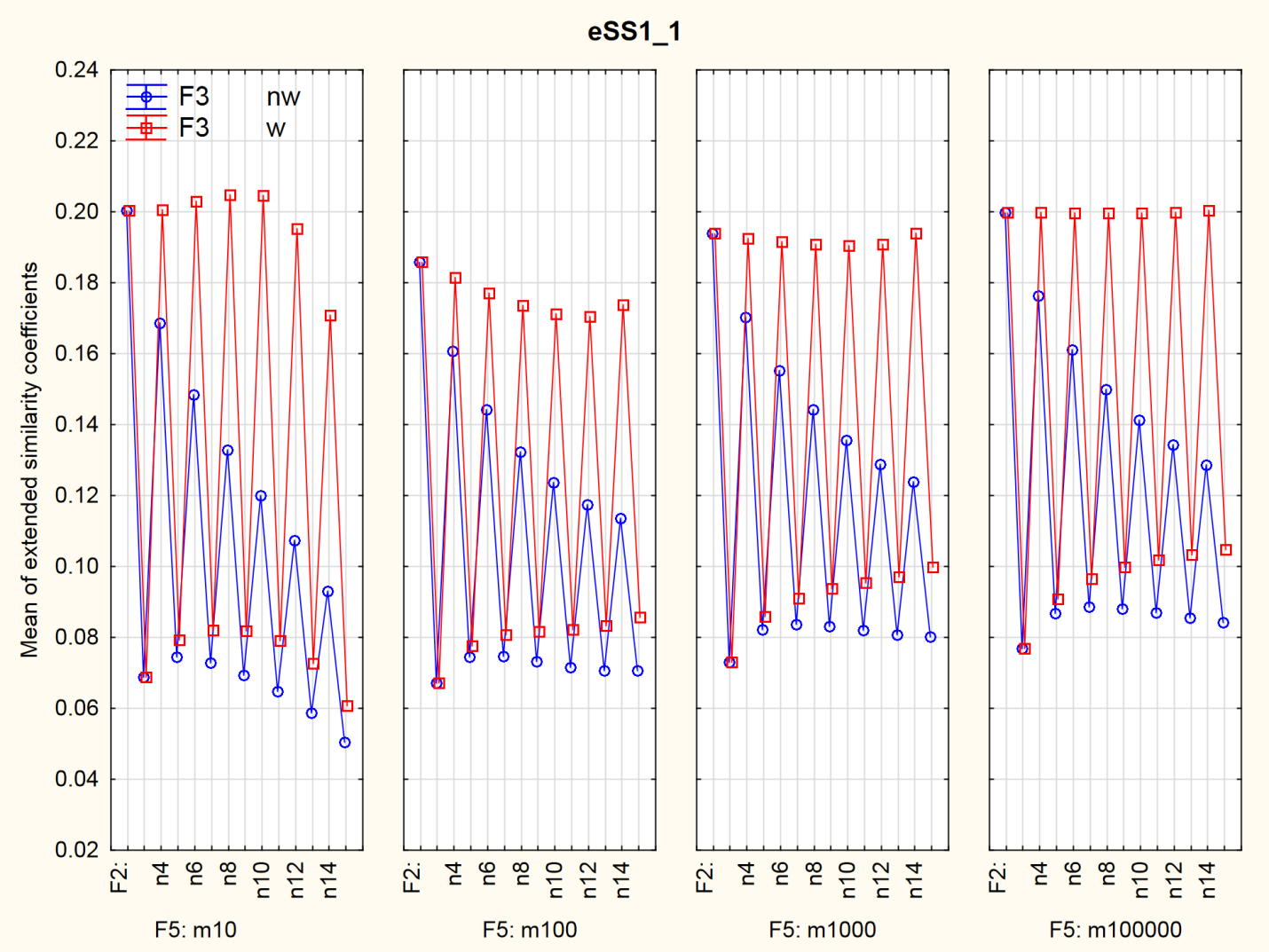

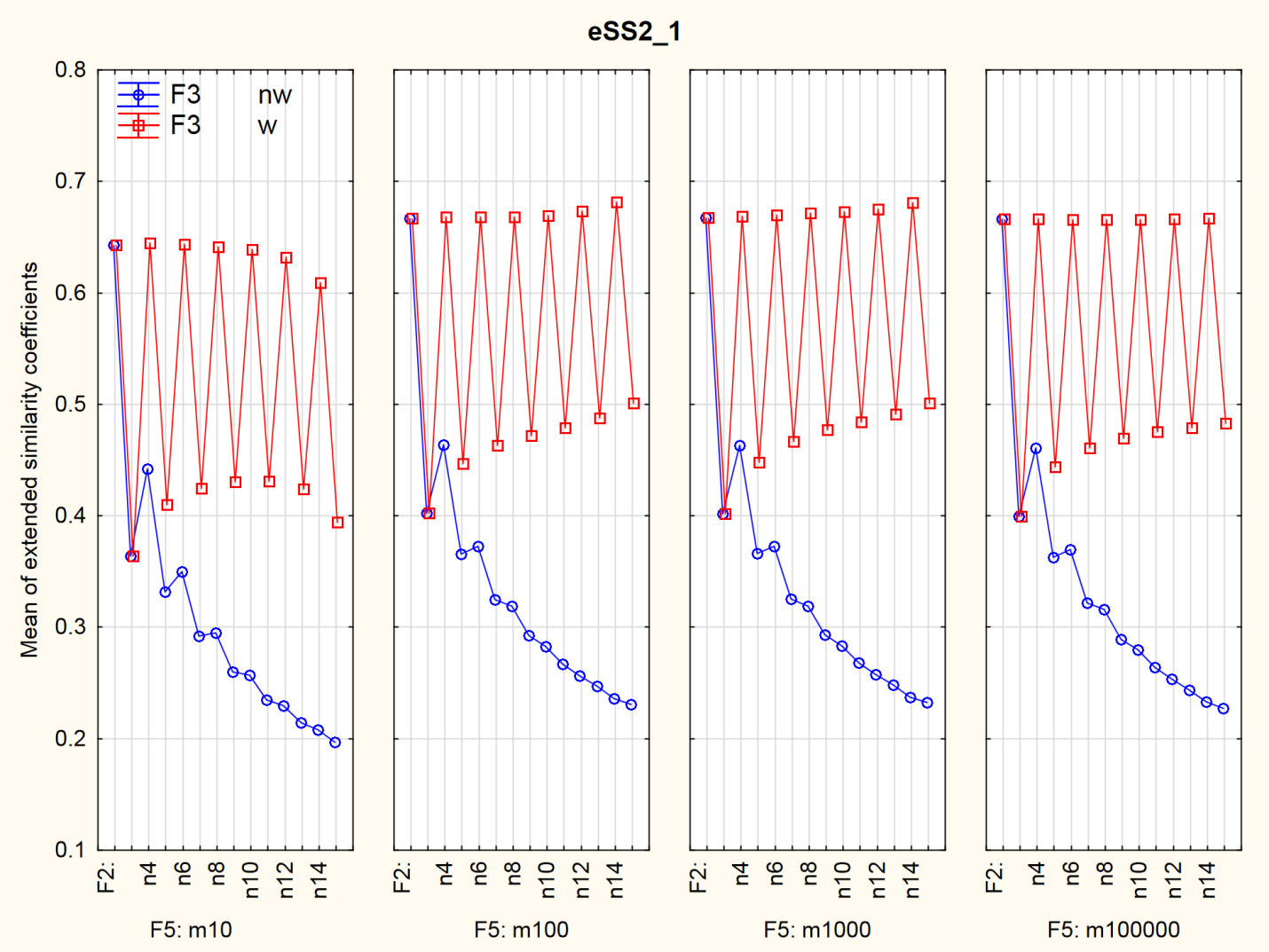
**


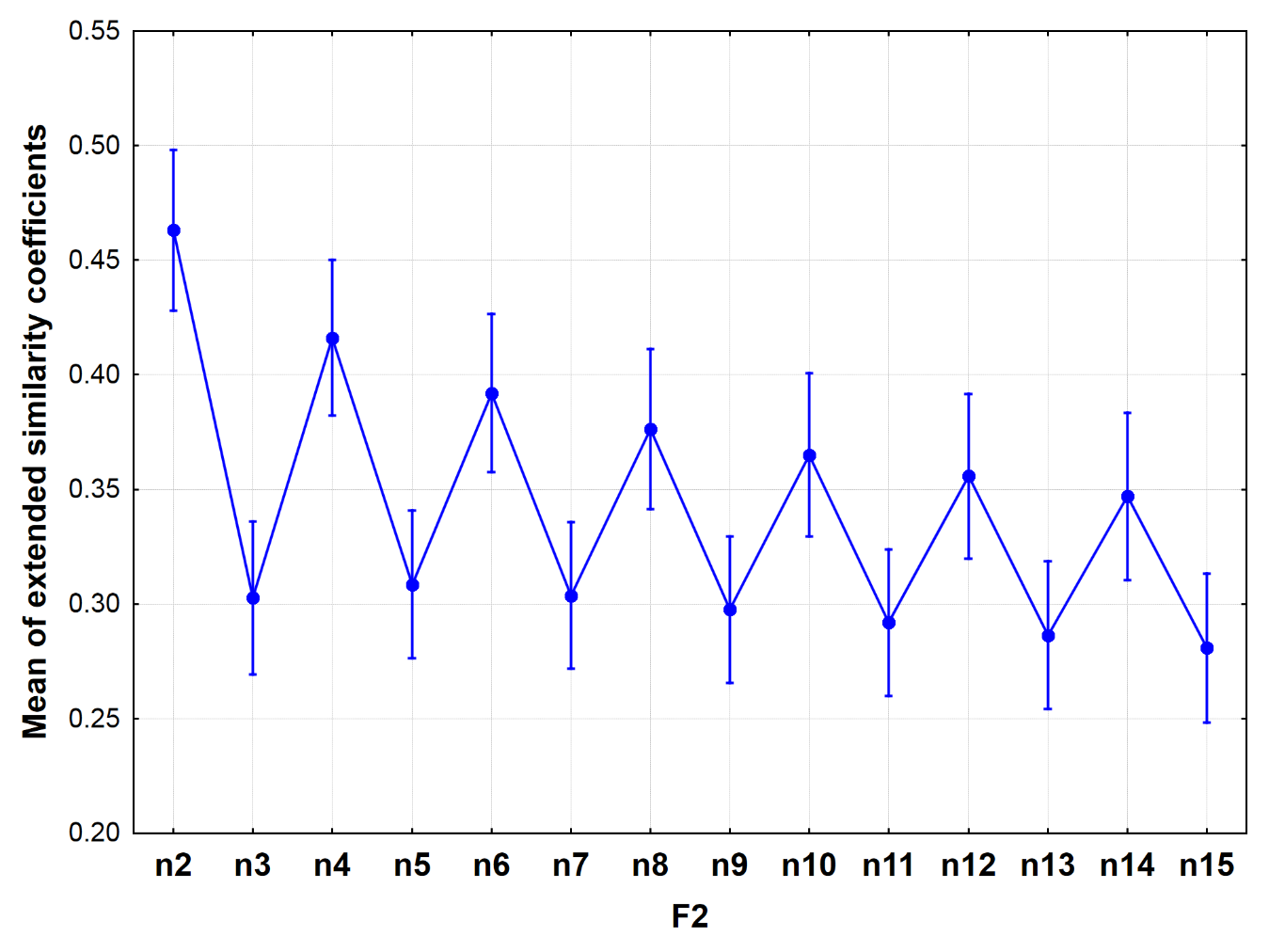


**Figure S20**. Means of extended similarity coefficients as a function of number of compared objects (*n*). Vertical bars denote 0.95 confidence intervals here and in all further ANOVA figures.


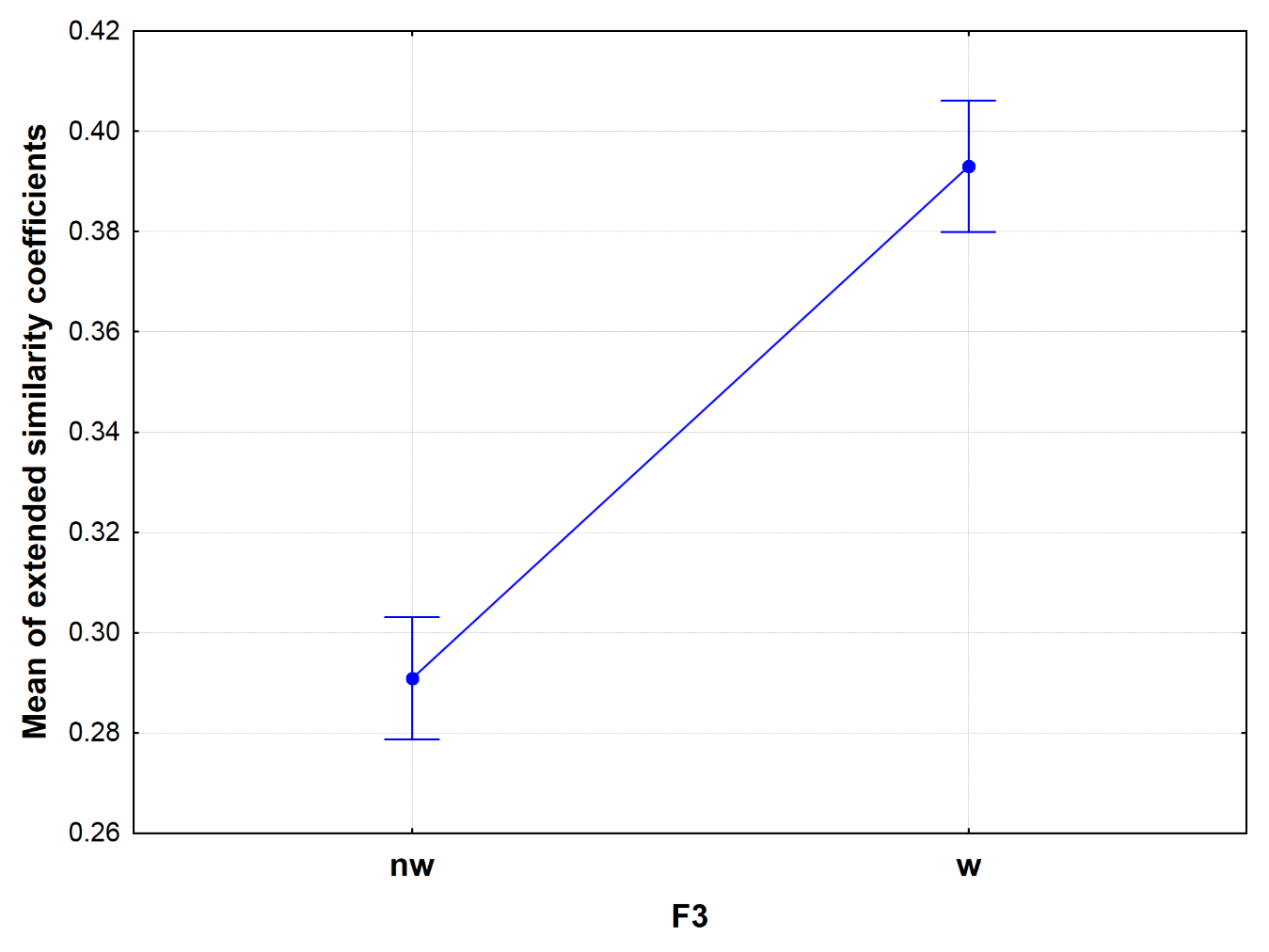


**Figure S21**. Means of the two kinds of extended similarity coefficients (w = weighted and nw = non-weighted).
